# Supplementary material for: Prior exposure to antiretroviral therapy among adult patients presenting for HIV treatment initiation or reinitiation in sub-Saharan Africa: a systematic review
Source: BMJ Open. 2023 Nov 19;13(11):e071283. doi: 10.1136/bmjopen-2022-071283 (PMC10660894; doi:10.1136/bmjopen-2022-071283)
Supplement: Supplementary data [file bmjopen-2022-071283supp001.pdf]

NIHR | National Institute for Health Research

PROSPERO
International prospective register of systematic reviews

To enable PROSPERO to focus on COVID-19 submissions, this registration record has undergone basic automated checks for eligibility and is published exactly as submitted. PROSPERO has never provided peer review, and usual checking by the PROSPERO team does not endorse content. Therefore, automatically published records should be treated as any other PROSPERO registration. Further detail is provided here.

Citation

Mariet Benade, Mhairi Maskew, Linda Sande, Nancy Scott, Allison Juntunen, Sydney Rosen. Prior exposure to antiretroviral therapy among adult patients presenting for HIV treatment initiation or re-initiation in sub-Saharan Africa: a systematic review. PROSPERO 2022 CRD42022324136 Available from: https://www.crd.york.ac.uk/prospero/display_record.php?ID=CRD42022324136

Review question

What proportions of adult patients presenting for ART initiation or re-initiation in public sector HIV treatment programs in Southern Africa are (a) ART naïve (new initiators) and (b) treatment experienced (re-initiators)?

Searches

We will search PubMed, Embase, and Web of Science and abstracts from the International AIDS Conference, International AIDS Society (IAS) Conference on HIV Science, and Conference on Retroviruses and Opportunistic Infections (CROI). We will manually search reference lists from sources identified in the search. Unpublished studies (e.g. preprints) will be included. Searches will be limited to English language publications.

The search period will be from January 1, 2018 to March 31, 2022. Inclusion in the review will be limited to sources for which a majority of individual-level data pertain to 2016 or later, as 2016 was the year when universal treatment access became common in sub-Saharan Africa. If sources report data from both before and after January 1, 2016, only results pertaining to the data from after that date will be used.

The search will be re-run prior to final analysis to identify very recent reports.

Types of study to be included

Studies that report initiation of ART for adult patients. These can be cohort, case control, cross-sectional, or interventional studies. White papers, commentaries, modeling studies, cost-effectiveness studies, and case studies will be excluded. Qualitative studies will be excluded unless there is specific data on treatment experience at ART initiation. Existing systematic reviews will be evaluated for additional, non-duplicate references. Published protocols with no data will be excluded, but if the study meets the inclusion criteria, we will attempt to find study results to evaluate for inclusion.

Condition or domain being studied

The successful scale-up of access to ART for HIV in sub-Saharan Africa has produced a growing population of patients who have interrupted or stopped treatment, and are now “re-initiating” therapy. While a diminishing number utilized ART temporarily for prevention of mother-to-child transmission prior to universal eligibility for ART, most are patients who stopped treatment, despite guidelines recommending lifelong use. Estimates of the proportion of patients presenting for ART re-initiation range from 10% to 50%.

Treatment re-initiators previously faced barriers to retention in care that they were unable to overcome. Achieving long-term retention in care targets may require that healthcare systems differentiate interventions and services for re-initiators from those offered to naïve initiators.

A first step in improving retention in care for ART re-initiators is to estimate how many individuals there are in any population of patients presenting for ART initiation. Few such estimates are available, largely because most healthcare data systems do not distinguish between naïve and re-initiators, allow tracking from one

healthcare facility to another or over long intervals of inactivity. To provide a baseline for considering this challenge, we'll search the recent literature for estimates of the proportion of patients who are re-initiators in sub-Saharan Africa.

Participants/population

Inclusion: Adults (?18 years of age) living with HIV presenting for initiation of any regimen of lifelong antiretroviral treatment in any sub-Saharan African country.
Exclusion: Individuals presenting for initiation of antiretroviral medications for any reason other than therapeutic (e.g. PrEP, PMTCT).

Intervention(s), exposure(s)

Initiation of ART is the intervention of interest. This will be an observational study of the characteristics of individuals initiating ART for HIV treatment (specifically, whether or not they have prior exposure to ART at the time of initiation).

Comparator(s)/control

Not applicable. Eligible studies need only report the proportions of patients who are treatment naïve and those who are treatment experienced.

Context

Initiation of ART in routine care settings in sub-Saharan Africa, including any type of facility or provider.

Main outcome(s)

Proportion of patients who are:
a. Treatment naïve at ART initiation, defined as a patient presenting for initiation of ART who has never previously taken antiretroviral therapy for treatment of HIV (new initiator); or
b. Treatment experienced at ART initiation, defined as a patient presenting for initiation of ART who has previously taken antiretroviral therapy for HIV treatment but has interrupted that therapy for a minimum of 3 months (re-initiator).

Measures of effect

Proportion, odds ratio

Additional outcome(s)

If data allow, we will also estimate the primary outcomes stratified by patient and/or facility characteristics, such as patient age and sex and facility country and setting.

Measures of effect

Proportion

Data extraction (selection and coding)

Study selection:

Rayyan will be used to support screening of studies and track inclusion or exclusion of studies. References will be managed in Mendeley, and duplicates will be removed. Two independent reviewers will be involved in each phase of review, including title and abstract screening, full-text screening, and data extraction. Title and abstract screening will include a pilot test to assess if the reviewers are able to achieve over 90% agreement on inclusion based on the eligibility criteria listed in this protocol. Disagreements will be resolved through discussion to achieve consensus. After full text screening, relevant non-duplicate citations from previous systematic reviews and other studies labelled for snowball in the initial search will be added for inclusion/exclusion consideration in a snowball phase.

Data extraction:

Data extraction will use a pre-determined template that captures all relevant variables. Data extraction will be done independently by two reviewers and discrepancies will be resolved by consensus. Data items to be extracted from studies include:

- Country
- District or locality
- Facility type
- Facility setting (urban, rural)
- Study year(s) or period(s) cohort observed
- Length of patient follow-up
- ART guidelines or protocol(s) in use during study period
- Study/cohort sample size
- Patient characteristics, including:
o Age distribution
o Gender distribution
o Clinical indicators of disease stage such as presenting CD4 count or WHO stage
o Other co-morbidities
- Study design (e.g., cohort, case control, cross-sectional, interventional)
- Descriptions of routine care, including whether the study was designed to influence retention
- Prior treatment exposure
o Proportion of adult patients ART treatment-naïve at initiation of ART
o Proportion of adult patients ART treatment-experienced at initiation of ART
o Basis for report of prior exposure (patient self-report, review of medical or laboratory records, tests of biological samples, pre-existing viral suppression, other)
- Authors' observations or comments on proportion of patients naïve or non-naïve, if any

Risk of bias (quality) assessment

Risk of bias and quality of individual studies that are included in this systematic review will be evaluated with the Joanna Briggs Institute critical appraisal checklist most relevant to the study type, as we expect that most included studies will be observational. The most important characteristics to be assessed will be recruitment biases (whether the study enrolled a representative sample of the specified population) and accuracy of naïve/non-naïve status reporting. Two reviewers will be involved in quality assessment, with disagreements resolved by a third reviewer on the study team.
We note that we expect that most studies meeting inclusion criteria for this review will report our outcomes (proportions naïve and non-naïve) as secondary outcomes or in their descriptions of their study cohorts, rather than as primary outcomes. (For example, a clinical trial with a primary outcome of viral suppression at 12 months after treatment initiation may report the proportion of participants who were naïve at initiation; our review would include the proportion naïve but not the primary outcome of viral suppression.)

Strategy for data synthesis

We will first present raw results from all included studies to demonstrate the breadth of outcomes, stratified by country. We will then estimate mean (95% confidence interval) and median (IQR) outcomes for each country and, to the extent that data allow, each population group or other stratification, such as setting. Pooled analysis of studies will only be conducted if ?3 studies are identified that report outcomes for the same populations (e.g., "adults initiating therapy at rural primary health clinics in Zambia").

Analysis of subgroups or subsets

If data allow, we plan to stratify our results by population (age, sex, condition, other characteristics) and facility characteristics (setting, level, size). We will not be able to specify all potential sub-group analyses until we determine which sub-groups are included in the publications reviewed.

Contact details for further information

Sydney Rosen

sbrosen@bu.edu

Supplemental material
BMJ Publishing Group Limited (BMJ) disclaims all liability and responsibility arising from any reliance placed on this supplemental material which has been supplied by the author(s)
BMJ Open

Organisational affiliation of the review

Boston University School of Public Health
https://www.bu.edu/sph/about/departments/global-health/

Review team members and their organisational affiliations

Dr Mariet Benade. Department of Global Health, Boston University School of Public Health, Boston, Massachusetts, USA
Dr Mhairi Maskew. Health Economics and Epidemiology Research Office, Faculty of Health Sciences, University of the Witwatersrand, Johannesburg, South Africa
Ms Linda Sande. Health Economics and Epidemiology Research Office, Faculty of Health Sciences, University of the Witwatersrand, Johannesburg, South Africa
Professor Nancy Scott. Department of Global Health, Boston University School of Public Health, Boston, Massachusetts, USA
Ms Allison Juntunen. Department of Global Health, Boston University School of Public Health, Boston, Massachusetts, USA
Professor Sydney Rosen. Department of Global Health, Boston University School of Public Health, Boston, Massachusetts, USA

Type and method of review

Epidemiologic, Systematic review

Anticipated or actual start date

10 April 2022

Anticipated completion date

10 July 2022

Funding sources/sponsors

Research reported in this publication was supported by the Bill & Melinda Gates Foundation Investment Number INV-031690, Retain6: Models of care for the first six months of HIV treatment.

Grant number(s)

State the funder, grant or award number and the date of award

Bill & Melinda Gates Foundation Investment Number INV-031690. October 2021.

Conflicts of interest

Language

English

Country

Malawi, South Africa, United States of America, Zambia

Stage of review

Review Ongoing

Subject index terms status

Subject indexing assigned by CRD

Subject index terms

MeSH headings have not been applied to this record

Date of registration in PROSPERO

08 May 2022

Date of first submission

NIHR | National Institute for Health Research

PROSPERO
International prospective register of systematic reviews

07 April 2022

Stage of review at time of this submission

Stage	Started	Completed
Preliminary searches	Yes	No
Piloting of the study selection process	No	No
Formal screening of search results against eligibility criteria	No	No
Data extraction	No	No
Risk of bias (quality) assessment	No	No
Data analysis	No	No

The record owner confirms that the information they have supplied for this submission is accurate and complete and they understand that deliberate provision of inaccurate information or omission of data may be construed as scientific misconduct.
The record owner confirms that they will update the status of the review when it is completed and will add publication details in due course.

Versions

08 May 2022
08 May 2022
