# Supplementary material for: Prior exposure to antiretroviral therapy among adult patients presenting for HIV treatment initiation or reinitiation in sub-Saharan Africa: a systematic review
Source: BMJ Open. 2023 Nov 19;13(11):e071283. doi: 10.1136/bmjopen-2022-071283 (PMC10660894; doi:10.1136/bmjopen-2022-071283)
Supplement: Supplementary data [file bmjopen-2022-071283supp004.pdf]

Additional table S1. Inclusion/exclusion criteria

Parameter	Inclusion criteria	Exclusion criteria
Population	Ages 18+ years; confirmed HIV positive status; presenting for initiation of any regimen of lifelong antiretroviral treatment	Paediatric and adolescent populations; currently only receiving ART for HIV prevention (PEP or PrEP)
Geographic region	Sub-Saharan Africa	None
Intervention	None, observational descriptive outcome	None
Study design	Reports primary, patient-level data from retrospective or prospective cohorts collected under any study design (trial, observational) with or without a comparison group; systematic reviews, meta-analyses	Case series or reports, purely qualitative studies, treatment guidelines, mathematical models, editorials, commentaries, study or trial protocols
Required descriptive data	Describes all of patients, location, timing of ART initiation, facility type, service delivery models and services provided to the public sector through government-managed public health infrastructure or through NGO/private programs or facilities that serve the uninsured sector	Insufficient description of the characteristics needed to describe the study population and outcome
Comparator	Not required; single arm evaluations are eligible	None
Outcomes	Reports proportion of patients initiating ART that are ART naïve and proportions of patients previously experienced on ART for any duration after initiation.	Insufficient detail provided to estimate of outcome
Timing	A majority of data collected for ART initiation on or after January 1, 2016	A majority of data accrued before January 1, 2016

Benade M, *et al. BMJ Open* 2023; 13:e071283. doi: 10.1136/bmjopen-2022-071283
