# Supplementary material for: Prior exposure to antiretroviral therapy among adult patients presenting for HIV treatment initiation or reinitiation in sub-Saharan Africa: a systematic review
Source: BMJ Open. 2023 Nov 19;13(11):e071283. doi: 10.1136/bmjopen-2022-071283 (PMC10660894; doi:10.1136/bmjopen-2022-071283)
Supplement: Supplementary data [file bmjopen-2022-071283supp005.pdf]

Additional table S2. Search strategy – conducted on 11 July 2023

**EMBASE**	
Population	('human immunodeficiency virus infection'/exp OR 'hiv infection')<br>**AND**
Intervention	('therapy'/exp OR ('anti human immunodeficiency virus agent'/exp OR 'antiretrovirus agent'/exp OR 'highly active antiretroviral therapy'/exp))<br>**AND**
Outcomes	('patient compliance'/exp OR 'treatment adherence' OR undisclosed OR 'retention'/exp)<br>**AND**
Context	(('africa south of the sahara'/exp OR 'africa south of the sahara' OR 'black africa' OR 'sub saharan africa' OR 'subsaharan africa') OR ('angola'/exp OR angola OR 'benin'/exp OR benin OR 'botswana'/exp OR botswana OR 'burkina faso'/exp OR 'burkina faso' OR 'burundi'/exp OR burundi OR 'cameroon'/exp OR cameroon OR 'cape verde'/exp OR 'cape verde' OR 'central african republic'/exp OR 'central african republic' OR 'chad'/exp OR chad OR 'comoros'/exp OR comoros OR 'congo'/exp OR congo OR brazzaville OR 'cote d ivoire' OR 'djibouti'/exp OR djibouti OR 'equatorial guinea'/exp OR 'equatorial guinea' OR 'eritrea'/exp OR eritrea OR 'ethiopia'/exp OR ethiopia OR 'gabon'/exp OR gabon OR 'gambia'/exp OR gambia OR 'ghana'/exp OR ghana OR 'guinea bissau'/exp OR 'guinea bissau' OR 'kenya'/exp OR kenya OR 'lesotho'/exp OR lesotho OR 'liberia'/exp OR liberia OR 'madagascar'/exp OR madagascar OR 'malawi'/exp OR malawi OR 'mali'/exp OR mali OR 'mauritania'/exp OR mauritania OR 'mauritius'/exp OR mauritius OR 'mozambique'/exp OR mozambique OR 'namibia'/exp OR namibia OR 'niger'/exp OR niger OR 'nigeria'/exp OR nigeria OR 'rwanda'/exp OR rwanda OR 'sao tome e principe' OR 'senegal'/exp OR senegal OR 'seychelles'/exp OR seychelles OR 'sierra leone'/exp OR 'sierra leone' OR 'somalia'/exp OR somalia OR 'south africa'/exp OR 'south africa' OR 'south sudan'/exp OR 'south sudan' OR 'sudan'/exp OR sudan OR 'swaziland'/exp OR swaziland OR 'tanzania'/exp OR tanzania OR 'togo'/exp OR togo OR 'uganda'/exp OR uganda OR 'western sahara'/exp OR 'western sahara' OR (western AND ('sahara'/exp OR sahara)) OR 'zaire'/exp OR zaire OR 'zambia'/exp OR zambia OR 'zimbabwe'/exp OR zimbabwe)) AND [2018-2023]/py
**PubMed**	
Population	("HIV Infections"[Mesh] OR HIV Infection)<br>**AND**
Intervention	((treatment OR "Anti-HIV Agents"[MESH] OR "Anti-Retroviral Agents"[MESH] OR "Antiretroviral Therapy, Highly Active"[MESH])<br>**AND**
Outcomes	(compliance OR "Treatment adherence" OR undisclosed OR retention)<br>**AND**
Context	("Africa South of the Sahara"[Mesh] OR Sub-Saharan Africa OR Subsaharan Africa OR Africa, Sub-Saharan OR Africa South of the Sahara OR Angola OR Benin OR Botswana OR "Burkina Faso" OR Burundi OR Cameroon OR "Cape Verde" OR "Central African Republic" OR Chad OR Comoros OR Congo OR Brazzaville OR "Cote d'Ivoire" OR Djibouti OR "Equatorial Guinea" OR Eritrea OR Ethiopia OR Gabon OR Gambia OR Ghana OR "Guinea Bissau" OR Kenya OR Lesotho OR Liberia OR Madagascar OR Malawi OR Mali OR Mauritania OR Mauritius OR Mozambique OR Namibia OR Niger OR Nigeria OR Rwanda OR "Sao Tome e Principe" OR Senegal OR Seychelles OR "Sierra Leone" OR Somalia OR "South

	Africa" OR "South Sudan" OR Sudan OR Swaziland OR Tanzania OR Togo OR Uganda OR Western Sahara OR Zaire OR Zambia OR Zimbabwe) AND (2018:2023[pdat])
**Web of Science Core Collection***	
Population	TS=("HIV Infections" OR HIV Infection)<br>**AND**
Intervention	TS=(treatment OR "Anti-HIV Agents" OR "Anti-Retroviral Agents" OR "Antiretroviral Therapy, Highly Active")<br>**AND**
Outcomes	TS= (compliance OR "Treatment adherence" OR undisclosed OR retention)<br>**AND**
Context	TS=("Africa South of the Sahara" OR Sub-Saharan Africa OR Subsaharan Africa OR Africa, Sub-Saharan OR Africa South of the Sahara OR Angola OR Benin OR Botswana OR "Burkina Faso" OR Burundi OR Cameroon OR "Cape Verde" OR "Central African Republic" OR Chad OR Comoros OR Congo OR Brazzaville OR "Cote d'Ivoire" OR Djibouti OR "Equatorial Guinea" OR Eritrea OR Ethiopia OR Gabon OR Gambia OR Ghana OR "Guinea Bissau" OR Kenya OR Lesotho OR Liberia OR Madagascar OR Malawi OR Mali OR Mauritania OR Mauritius OR Mozambique OR Namibia OR Niger OR Nigeria OR Rwanda OR "Sao Tome e Principe" OR Senegal OR Seychelles OR "Sierra Leone" OR Somalia OR "South Africa" OR "South Sudan" OR Sudan OR Swaziland OR Tanzania OR Togo OR Uganda OR Western Sahara OR Zaire OR Zambia OR Zimbabwe)
**Conference searches**	
Search terms	“initiation”, “naïve”, “re-initiate”, “newly”, “experience”
Web URLs for conferences searched	International AIDS Conference<br>2018 - https://www.aids2018.org<br>2020 - https://www.aids2020.org<br>2022 - https://www.aids2022.org<br><br>International AIDS Society (IAS) Conference on HIV Science<br>2019 - https://programme.ias2019.org<br>2021 - https://ias2021.org<br><br>Conference on Retroviruses and Opportunistic Infections (CROI)<br>2018 - https://www.croiconference.org/croi-2018/<br>2019 - https://www.croiconference.org/croi-2019/<br>2020 - https://www.croiconference.org/croi-2020/<br>2021 - https://www.croiconference.org/croi-2021/<br>2022 - https://www.croiconference.org/croi-2022/

* Comprised of the Science Citation Index Expanded (1965 to the present), the Social Sciences Citation Index (1965 to the present), the Arts & Humanities Citation Index (1975 to the present), the Conference Proceedings Citation Index (both versions, Science and Social Sciences & the Humanities from 1990 to the present), the Book Citation Index (both versions, Science and Social Sciences & the Humanities from 2005 to the present) and the Emerging Sources Citation Index (2018 to the present)

Note: Results limited to 1 January 2018 – 11 July 2023

Benade M, *et al. BMJ Open* 2023; 13:e071283. doi: 10.1136/bmjopen-2022-071283
