# Supplementary material for: Prior exposure to antiretroviral therapy among adult patients presenting for HIV treatment initiation or reinitiation in sub-Saharan Africa: a systematic review
Source: BMJ Open. 2023 Nov 19;13(11):e071283. doi: 10.1136/bmjopen-2022-071283 (PMC10660894; doi:10.1136/bmjopen-2022-071283)
Supplement: Supplementary data [file bmjopen-2022-071283supp006.pdf]

Additional table S3. Reasons for exclusion after full text review

Reason and number excluded	Explanation
Wrong time period (n=95)	All data or majority of data were gathered prior to 2016.
Wrong publication type (n=12)	Article was a protocol, review article or qualitative report without any quantitative descriptors.
Wrong population (n=129)	Population for main analysis included participants younger than 18yrs without stratification in a setting where adults are defined as older than 18yrs.<br>Population for main analysis excluded those with ART experience, unless other measures taken to confirm ART naivety. Population did not consists of participants presenting for ART initiation.
Not sufficient data for analysis (n=132)	No exclusion criteria met, but data reported was not sufficient for us to determine naïve vs non-naïve status.

Benade M, *et al. BMJ Open* 2023; 13:e071283. doi: 10.1136/bmjopen-2022-071283
