# Supplementary material for: Prior exposure to antiretroviral therapy among adult patients presenting for HIV treatment initiation or reinitiation in sub-Saharan Africa: a systematic review
Source: BMJ Open. 2023 Nov 19;13(11):e071283. doi: 10.1136/bmjopen-2022-071283 (PMC10660894; doi:10.1136/bmjopen-2022-071283)
Supplement: Supplementary data [file bmjopen-2022-071283supp003.pdf]

Supplemental file 3:
Systematic reviews screened for potential articles

1. Abdulrahman SA, Ganasegeran K, Rampal L, Martins OF. HIV Treatment Adherence - A Shared Burden for Patients, Health-Care Providers, and Other Stakeholders. AIDS Rev. 2019;21: 28–39. doi:10.24875/AIDSRev.19000037
2. Abebe Moges N, Olubukola A, Micheal O, Berhane Y. HIV patients retention and attrition in care and their determinants in Ethiopia: a systematic review and meta-analysis. BMC Infect Dis. 2020;20: 439. doi:10.1186/s12879-020-05168-3
3. Adugna Wubneh C, Dessalegn Mekonnen B, Wesenyeleh Delelegn M, Asmare Atalell K. Adherence to option B+ and its association with disclosure status and counseling among HIV-positive pregnant and lactating women in Ethiopia: systematic review and meta-analysis. Public Health. 2022;211: 105–113. doi:10.1016/J.PUHE.2022.07.016
4. Agutu CA, Ngetsa CJ, Price MA, Rinke de Wit TF, Omosa-Manyonyi G, Sanders EJ, et al. Systematic review of the performance and clinical utility of point of care HIV-1 RNA testing for diagnosis and care. PLoS One. 2019;14: e0218369. doi:10.1371/journal.pone.0218369
5. Ale BM, Amahowe F, Nganda MM, Danwang C, Wakaba NN, Almuwallad A, et al. Global burden of active smoking among people living with HIV on antiretroviral therapy: a systematic review and meta-analysis. Infect Dis Poverty. 2021;10: 12. doi:10.1186/s40249-021-00799-3
6. Belayneh Z, Mekuriaw B, Mehare T, Shumye S, Tsehay M. Magnitude and predictors of common mental disorder among people with HIV/AIDS in Ethiopia: a systematic review and meta-analysis. BMC Public Health. 2020;20: 689.
7. Brault MA, Spiegelman D, Abdool Karim SS, Vermund SH. Integrating and Interpreting Findings from the Latest Treatment as Prevention Trials. Curr HIV/AIDS Rep. 2020;17: 249–258. doi:10.1007/s11904-020-00492-4
8. Bulstra C, Hontelez J, Otto M, Stepanova A, Lamontagne E, Yakusik A, et al. Integrating HIV services and other health services: A systematic review and meta-analysis. Nosyk B, editor. PLoS Med. 2021;18: e1003836. doi:10.1371/journal.pmed.1003836
9. Casale M, Carlqvist A, Cluver L. Recent Interventions to Improve Retention in HIV Care and Adherence to Antiretroviral Treatment Among Adolescents and Youth: A Systematic Review. AIDS Patient Care STDS. 2019;33: 237–252.
10. Chammartin F, Zürcher K, Keiser O, Weigel R, Chu K, AN K, et al. Outcomes of Patients Lost to Follow-up in African Antiretroviral Therapy Programs: Individual Patient Data Meta-analysis. Clin Infect Dis an Off Publ Infect Dis Soc Am. 2018;67: 1643–1652. doi:10.1093/cid/ciy347
11. Chem ED, Van Hout MC, Hope V. Treatment outcomes and antiretroviral uptake in multidrug-resistant tuberculosis and HIV co-infected patients in Sub Saharan Africa: a systematic review and meta-analysis. BMC Infect Dis. 2019;19.
12. Chem E, Ferry A, Seeley J, HA W, Simms V. Health-related needs reported by adolescents living with HIV and receiving antiretroviral therapy in sub-Saharan Africa: a systematic literature review. J Int AIDS Soc. 2022;25: e25921. doi:10.1002/jia2.25921
13. Chimatira R, Ross A. A rapid review and synthesis of the effectiveness of programmes

initiating community-based antiretroviral therapy in sub-Saharan Africa. South Afr J HIV Med. 2020;21. doi:10.4102/SAJHIVMED.V21I1.1153

14. Cohn J, Ake J, Moorhouse M, Godfrey C. Sex Differences in the Treatment of HIV. Curr HIV/AIDS Rep. 2020;17: 373–384. doi:10.1007/s11904-020-00499-x
15. Damtie Y, Kefale B, Yalew M, Arefaynie M, Adane B, Muche A, et al. HIV risk behavior and associated factors among people living with HIV/AIDS in Ethiopia: A systematic review and meta-analysis. PLoS One. 2022;17: e0269304. doi:10.1371/journal.pone.0269304
16. Damulak PP, Ismail S, Abdul Manaf R, Mohd Said S, Agbaji O. Interventions to Improve Adherence to Antiretroviral Therapy (ART) in Sub-Saharan Africa: An Updated Systematic Review. Int J Env Res Public Heal. 2021;18. doi:10.3390/ijerph18052477
17. De Tomasi K, Mouala C. Good practices for retention in the circuit of prevention of Mother-Child Transmission of HIV in Sub-Saharan Africa: a systematic review of the literature. Med Sante Trop. 2019;29: 279–286. doi:10.1684/mst.2019.0911
18. Demena BA, Artavia-Mora L, Ouedraogo D, Thiombiano BA, Wagner N. A Systematic Review of Mobile Phone Interventions (SMS/IVR/Calls) to Improve Adherence and Retention to Antiretroviral Treatment in Low-and Middle-Income Countries. AIDS Patient Care STDS. 2020;34: 59–71. doi:10.1089/apc.2019.0181
19. Dessie G, Wagnew F, Mulugeta H, Amare D, Jara D, CT L, et al. The effect of disclosure on adherence to antiretroviral therapy among adults living with HIV in Ethiopia: a systematic review and meta-analysis. BMC Infect Dis. 2019;19: 528.
20. Diallo M, Adekpedjou R, Ahouada C, Ngangue P, Ly BA. Impact of pre-antiretroviral therapy cd4 counts on drug resistance and treatment failure: A systematic review. AIDS Rev. 2020;22: 78–92. doi:10.24875/AIDSRev.20000012
21. Dovel KL, Hariprasad S, Hubbard J, Cornell M, Phiri K, Choko A, et al. Strategies to improve antiretroviral therapy (ART) initiation and early engagement among men in sub-Saharan Africa: A scoping review of interventions in the era of universal treatment. Trop Med Int Heal. 2023;28: 454–465. doi:10.1111/tmi.13880
22. Duffy M, Madevu-Matson C, JE P, Zwick H, Sharer M, AM P. Systematic review: Development of a person-centered care framework within the context of HIV treatment settings in sub-Saharan Africa. Trop Med Int Health. 2022;27: 479–493. doi:10.1111/tmi.13746
23. Edessa D, Sisay M, Asefa F. Second-line HIV treatment failure in sub-Saharan Africa: A systematic review and meta-analysis. PLoS One. 2019;14: e0220159. doi:10.1371/journal.pone.0220159
24. Endalamaw A, Mekonnen M, Geremew D, Yehualashet FA, Tesera H, Habtewold TD. HIV/AIDS treatment failure and associated factors in Ethiopia: meta-analysis. BMC Public Health. 2020;20: 82. doi:10.1186/s12889-020-8160-8
25. Enslin D, Mallya P. Factors influencing treatment adherence in hypertension and HIV management in South Africa: A comparative literature review. South African Fam Pract. 2022;64. doi:10.4102/SAFP.V64I1.5434
26. Eshun-Wilson I, Awotiwon AA, Germann A, Amankwaa SA, Ford N, Schwartz S, et al. Effects of community-based antiretroviral therapy initiation models on HIV treatment outcomes: A systematic review and meta-analysis. PLoS Med. 2021;18: e1003646. doi:10.1371/journal.pmed.1003646

27. Finlay J, Lambert T, Krahn J, Meyer G, Singh AE, Caine V. Incentive-Based Human Immunodeficiency Virus Screening in Low- and Middle-Income Countries: A Systematic Review. Sex Transm Dis. 2022;49: 274–283. doi:10.1097/OLQ.0000000000001567
28. Fite RO. Association between adherence to Antiretroviral Therapy and place of residence among adult HIV infected patients in Ethiopia: A systematic review and meta-analysis. PLoS One. 2021;16: e0256948. doi:10.1371/journal.pone.0256948
29. Ford N, Migone C, Calmy A, Kerschberger B, Kanters S, Nsanzimana S, et al. Benefits and risks of rapid initiation of antiretroviral therapy. Aids. 2018;32: 17–23. doi:10.1097/qad.0000000000001671
30. Frijters EM, Hermans LE, Wensing AMJ, Devillé WLJM, Tempelman HA, De Wit JBF. Risk factors for loss to follow-up from antiretroviral therapy programmes in low-income and middle-income countries. AIDS. 2020;34: 1261–1288.
31. Fuseini H, Gyan BA, Kyei GB, Heimburger DC, Koethe JR. Undernutrition and HIV Infection in Sub-Saharan Africa: Health Outcomes and Therapeutic Interventions. Curr HIV/AIDS Rep. 2021;18: 87–97. doi:10.1007/s11904-021-00541-6
32. Geremew D, Melku M, Endalamaw A, Woldu B, Fasil A, Negash M, et al. Tuberculosis and its association with CD4(+) T cell count among adult HIV positive patients in Ethiopian settings: a systematic review and meta-analysis. BMC Infect Dis. 2020;20: 325.
33. Hagey JM, Li X, Barr-Walker J, Penner J, Kadima J, Oyaro P, et al. Differentiated HIV care in sub-Saharan Africa: a scoping review to inform antiretroviral therapy provision for stable HIV-infected individuals in Kenya. AIDS Care. 2018;30: 1477–1487. doi:10.1080/09540121.2018.1500995
34. Haghighat R, Steinert J, Cluver L. The effects of decentralising antiretroviral therapy care delivery on health outcomes for adolescents and young adults in low- and middle-income countries: a systematic review. Glob Health Action. 2019;12: 1668596. doi:10.1080/16549716.2019.1668596
35. Hargreaves J, Dalal S, Rice B, Anderegg N, Bhattacharjee P, Gafos M, et al. Repositioning Implementation Science in the HIV Response: Looking Ahead From AIDS 2018. J Acquir Immune Defic Syndr. 2019;82: S299–S304. doi:10.1097/QAI.0000000000002209
36. Hlongwa M, Mashamba-Thompson T, Makhunga S, Hlongwana K. Mapping evidence of intervention strategies to improving men’s uptake to HIV testing services in sub-Saharan Africa: A systematic scoping review. BMC Infect Dis. 2019;19: 496. doi:10.1186/s12879-019-4124-y
37. Ibiloye O, Masquillier C, Jwanle P, S VB, J van O, Lynen L, et al. Community-Based ART Service Delivery for Key Populations in Sub-Saharan Africa: Scoping Review of Outcomes Along the Continuum of HIV Care. AIDS Behav. 2022;26: 2314–2337. doi:10.1007/s10461-021-03568-3
38. Jardim C, Zamani R, Akrami M. Evaluating the Impact of the COVID-19 Pandemic on Accessing HIV Services in South Africa: A Systematic Review. Int J Environ Res Public Health. 2022;19. doi:10.3390/ijerph191911899
39. Jopling R, Nyamayaro P, Andersen LS, Kagee A, Haberer JE, Abas MA. A Cascade of Interventions to Promote Adherence to Antiretroviral Therapy in African Countries. Curr HIV/AIDS Rep. 2020;17: 529–546. doi:10.1007/s11904-020-00511-4
40. Kadia BM, Dimala CA, Fongwen NT, Smith AD. Barriers to and enablers of uptake of

antiretroviral therapy in integrated HIV and tuberculosis treatment programmes in sub-Saharan Africa: a systematic review and meta-analysis. AIDS Res Ther. 2021;18. doi:10.1186/s12981-021-00395-3

41. Kebede HK, Mwanri L, Ward P, Gesesew HA. Predictors of lost to follow up from antiretroviral therapy among adults in sub-Saharan Africa: a systematic review and meta-analysis. Infect Dis POVERTY. 2021;10. doi:10.1186/s40249-021-00822.
42. Knight L, FC M, Schatz E. Behavioral and cognitive interventions to improve treatment adherence and access to HIV care among older adults in sub-Saharan Africa: an updated systematic review. Syst Rev. 2018;7: 114.
43. Kusemererwa S, Akena D, Nakanjako D, Kigozi J, Nanyunja R, Nanfuka M, et al. Strategies for retention of heterosexual men in HIV care in sub-Saharan Africa: A systematic review. PLoS One. 2021;16: e0246471. doi:10.1371/journal.pone.0246471
44. Laurenzi CA, du Toit S, Ameyan W, Melendez-Torres GJ, Kara T, Brand A, et al. Psychosocial interventions for improving engagement in care and health and behavioural outcomes for adolescents and young people living with HIV: a systematic review and meta-analysis. J Int AIDS Soc. 2021;24.
45. Le Tourneau N, Germann A, Thompson RR, Ford N, Schwartz S, Beres L, et al. Evaluation of HIV treatment outcomes with reduced frequency of clinical encounters and antiretroviral treatment refills: A systematic review and meta-analysis. PLoS Med. 2022;19: e1003959. doi:10.1371/journal.pmed.1003959
46. Limbada M, Zijlstra G, Macleod D, Ayles H, Fidler S. A systematic review of the effectiveness of non- health facility based care delivery of antiretroviral therapy for people living with HIV in sub-Saharan Africa measured by viral suppression, mortality and retention on ART. BMC Public Health. 2021;21: 1110. doi:10.1186/s12889-021-11053-8
47. Long L, Kuchukhidze S, Pascoe S, BE N, MP F, Cele R, et al. Retention in care and viral suppression in differentiated service delivery models for HIV treatment delivery in sub-Saharan Africa: a rapid systematic review. J Int AIDS Soc. 2020;23: e25640. doi:10.1002/jia2.25640
48. Makhado L, Mongale MP. Factors influencing non-adherence to antiretroviral therapy in South Africa: A systematic review. HIV AIDS Rev. 2019;18: 239–246.
49. Mateo-Urdiales A, Johnson S, Smith R, Nachega JB, Eshun-Wilson I. Rapid initiation of antiretroviral therapy for people living with HIV. Cochrane Database Syst Rev. 2019;2019. doi:10.1002/14651858.CD012962.pub2
50. Menon S, Benova L, Mabeya H. Epilepsy management in pregnant HIV plus women in sub-Saharan Africa, clinical aspects to consider: a scoping review. BMC Med. 2020;18.
51. Mugglin C. Gueler A., Vanobberghen F., Rice B., Egger M. KD. Apples and oranges: Assessment of the care cascade in sub-Saharan Africa. 22. doi:10.1002/jia2.25327
52. Muhula S, Gachohi J, Kombe Y, Karanja S. Interventions to improve early retention of patients in antiretroviral therapy programmes in sub-Saharan Africa: A systematic review. PLoS One. 2022;17: e0263663. doi:10.1371/journal.pone.0263663
53. Mukumbang FC, Orth Z, Van Wyk B. What do the implementation outcome variables tell us about the scaling-up of the antiretroviral treatment adherence clubs in South Africa? A document review. Heal Res Policy Syst. 2019;17.
54. Musumari PM, Techasrivichien T, Srithanaviboonchai K, Wanyenze RK, Matovu JKB,

Poudyal H, et al. HIV epidemic in fishing communities in Uganda: A scoping review. PLoS One. 2021;16.
55. Necho M, Belete A, Tsehay M. Depressive symptoms and their determinants in patients who are on antiretroviral therapy in the case of a low-income country, Ethiopia: a systematic review and meta-analysis. Int J Ment Health Syst. 2021;15. doi:10.1186/s13033-020-00430-2
56. Nyato D, Kuringe E, Drake M, Casalini C, Nnko S, Shao A, et al. Participants' accrual and delivery of HIV prevention interventions among men who have sex with men in sub-Saharan Africa: a systematic review. BMC Public Health. 2018;18: 370. doi:10.1186/s12889-018-5303-2
57. Nyoni T, Sallah YH, Okumu M, Byansi W, Lipsey K, Small E. The effectiveness of treatment supporter interventions in antiretroviral treatment adherence in sub-Saharan Africa: a systematic review and meta-Analysis. AIDS Care Psychol Socio-Med Asp AIDS HIV. 2020;32: 214–227.
58. Okonji EF, Mukumbang FC, Orth Z, Vickerman-Delport SA, Van Wyk B. Psychosocial support interventions for improved adherence and retention in ART care for young people living with HIV (10-24,Ääyears): a scoping review. BMC Public Health. 2020;20: 1841. doi:10.1186/s12889-020-09717-y
59. Omonaiye O, Kusljic S, Nicholson P, Manias E. Medication adherence in pregnant women with human immunodeficiency virus receiving antiretroviral therapy in sub-Saharan Africa: a systematic review. BMC Public Health. 2018;18: 805. doi:10.1186/s12889-018-5651-y
60. Omonaiye O, Nicholson P, Kusljic S, Manias E. A meta-analysis of effectiveness of interventions to improve adherence in pregnant women receiving antiretroviral therapy in sub-Saharan Africa. Int J Infect Dis IJID Off Publ Int Soc Infect Dis. 2018;74: 71–82.
61. Owusu K.K. Ahmed Z. A-GR. Strategies to improve linkage to hiv care in urban areas of Sub-Saharan Africa: A systematic review. 11. doi:10.2147/HIV.S216093
62. Passchier RV, Abas MA, Ebuenyi ID, Pariante CM. Effectiveness of depression interventions for people living with HIV in Sub-Saharan Africa: A systematic review & meta-analysis of psychological & immunological outcomes. Brain Behav Immun. 2018;73: 261–273. doi:10.1016/j.bbi.2018.05.010
63. Pellowski JA, Price DM, Harrison AD, Tuthill EL, Myer L, Operario D, et al. A Systematic Review and Meta-analysis of Antiretroviral Therapy (ART) Adherence Interventions for Women Living with HIV. AIDS Behav. 2019;23: 1998–2013. doi:10.1007/s10461-018-2341-9
64. Penn A, Azman H, Horvath H, KD T, MD H, Rajan J, et al. Supportive interventions to improve retention on ART in people with HIV in low- and middle-income countries: A systematic review. PloS one. 2018. p. e0208814. doi:10.1371/journal.pone.0208814
65. Rapaport SF, Peer AD, Viswasam N, Hahn E, Ryan S, Turpin G, et al. Implementing HIV Prevention in Sub-Saharan Africa: A Systematic Review of Interventions Targeting Systems, Communities, and Individuals. AIDS Behav. doi:10.1007/s10461-022-03751-0
66. Raphael M, Elizabeth S, Fiona V, Maja W. "Linkage to care" among people living with HIV in the era of "universal test and treat" in a sub-Sahara African setting. SWISS Med Wkly.

2021;151. doi:10.4414/smw.2021.20535
67. Roy M, Bolton Moore C, Sikazwe I, Holmes CB. A Review of Differentiated Service Delivery for HIV Treatment: Effectiveness, Mechanisms, Targeting, and Scale. Current HIV/AIDS Reports. Current Medicine Group LLC 1; 2019. pp. 324–334. doi:10.1007/s11904-019-00454-5
68. Sabapathy K, Hensen B, Varsaneux O, Floyd S, Fidler S, Hayes R. The cascade of care following community-based detection of HIV in sub-Saharan Africa – A systematic review with 90-90-90 targets in sight. PLoS One. 2018;13: e0200737. doi:10.1371/JOURNAL.PONE.0200737
69. Takarinda KC, Wallenta J, Scheve A, Mody A, Apollo T, Harries AD, et al. Measuring Retention in Antiretroviral Therapy Programs—a Synthetic Review of Different Approaches for Field Use in Low- and Middle-Income Settings. Curr Trop Med Reports. 2018;5: 179–185. doi:10.1007/S40475-018-0153-7
70. Too EK, Abubakar A, Nasambu C, Koot HM, Cuijpers P, Newton CRJC, et al. Prevalence and factors associated with common mental disorders in young people living with HIV in sub-Saharan Africa: a systematic review. J Int AIDS Soc. 2021;24.
71. Vrazo A, Firth J, Amzel A, Sedillo R, Ryan J, BR P. Interventions to significantly improve service uptake and retention of HIV-positive pregnant women and HIV-exposed infants along the prevention of mother-to-child transmission continuum of care: systematic review. Trop Med Int Health. 2018;23: 136–148. doi:10.1111/tmi.13014
72. Walsh F, Khan S, Bärnighausen T, Hettema A, Lejeune C, Mazibuko S, et al. Getting to 90-90-90: Experiences from the MaxART Early Access to ART for All (EAAA) Trial in Eswatini. Curr HIV/AIDS Rep. 2020;17: 324–332. doi:10.1007/s11904-020-00501-6
73. Yah CS, Tambo E. Why is mother to child transmission (MTCT) of HIV a continual threat to new-borns in sub-Saharan Africa (SSA). J Infect Public Health. 2019;12: 213–223.
74. Yendewa GA, Poveda E, Yendewa SA, Sahr F, Quiñones-Mateu ME, Salata RA. HIV/AIDS in Sierra Leone: Characterizing the Hidden Epidemic. AIDS Rev. 2018;20: 104–113. doi:10.24875/AIDSRev.M18000022
75. Yonga AM, Kiss L, Onarheim KH. A systematic review of the effects of intimate partner violence on HIV-positive pregnant women in sub-Saharan Africa. BMC Public Health. 2022;22: 220.
76. Zanoni B, Archary M, Sibaya T, Ramos T, Donenberg G, Shahmanesh M, et al. Interventions addressing the adolescent HIV continuum of care in South Africa: a systematic review and modified Delphi analysis. BMJ Open. 2022;12: e057797. doi:10.1136/bmjopen-2021-057797
