# Supplementary material for: Prior exposure to antiretroviral therapy among adult patients presenting for HIV treatment initiation or reinitiation in sub-Saharan Africa: a systematic review
Source: BMJ Open. 2023 Nov 19;13(11):e071283. doi: 10.1136/bmjopen-2022-071283 (PMC10660894; doi:10.1136/bmjopen-2022-071283)
Supplement: Author's manuscript [file bmjopen-2022-071283.draft_revisions.pdf]

BMJ Open is committed to open peer review. As part of this commitment we make the peer review history of every article we publish publicly available.

When an article is published we post the peer reviewers' comments and the authors' responses online. We also post the versions of the paper that were used during peer review. These are the versions that the peer review comments apply to.

The versions of the paper that follow are the versions that were submitted during the peer review process. They are not the versions of record or the final published versions. They should not be cited or distributed as the published version of this manuscript.

BMJ Open is an open access journal and the full, final, typeset and author-corrected version of record of the manuscript is available on our site with no access controls, subscription charges or pay-per-view fees (http://bmjopen.bmj.com).

If you have any questions on BMJ Open's open peer review process please email info.bmjopen@bmj.com

BMJ Open

Prior exposure to antiretroviral therapy among adult patients presenting for HIV treatment initiation or re-initiation in sub- Saharan Africa: a systematic review

Journal:	*BMJ Open*
Manuscript ID	bmjopen-2022-071283
Article Type:	Original research
Date Submitted by the Author:	20-Dec-2022

Complete List of Authors:	Benade, Mariet; Boston University School of Public Health, Department of Global Health<br>Maskew, Mhairi; University of the Witwatersrand Johannesburg Faculty of Health Sciences, Health Economics and Epidemiology Research Office<br>Juntunen, Allison; Boston University School of Public Health, Department of Global Health<br>Rosen, Sydney; University of the Witwatersrand Johannesburg Faculty of Health Sciences, Health Economics and Epidemiology Research Office; Boston University School of Public Health, Department of Global Health

Keywords:	EPIDEMIOLOGY, HIV & AIDS < INFECTIOUS DISEASES, PUBLIC HEALTH, Health Services Accessibility

SCHOLARONE™
Manuscripts

I, the Submitting Author has the right to grant and does grant on behalf of all authors of the Work (as defined in the below author licence), an exclusive licence and/or a non-exclusive licence for contributions from authors who are: i) UK Crown employees; ii) where BMJ has agreed a CC-BY licence shall apply, and/or iii) in accordance with the terms applicable for US Federal Government officers or employees acting as part of their official duties; on a worldwide, perpetual, irrevocable, royalty-free basis to BMJ Publishing Group Ltd ("BMJ") its licensees and where the relevant Journal is co-owned by BMJ to the co-owners of the Journal, to publish the Work in this journal and any other BMJ products and to exploit all rights, as set out in our licence.

The Submitting Author accepts and understands that any supply made under these terms is made by BMJ to the Submitting Author unless you are acting as an employee on behalf of your employer or a postgraduate student of an affiliated institution which is paying any applicable article publishing charge ("APC") for Open Access articles. Where the Submitting Author wishes to make the Work available on an Open Access basis (and intends to pay the relevant APC), the terms of reuse of such Open Access shall be governed by a Creative Commons licence – details of these licences and which Creative Commons licence will apply to this Work are set out in our licence referred to above.

Other than as permitted in any relevant BMJ Author's Self Archiving Policies, I confirm this Work has not been accepted for publication elsewhere, is not being considered for publication elsewhere and does not duplicate material already published. I confirm all authors consent to publication of this Work and authorise the granting of this licence.

For peer review only

Prior exposure to antiretroviral therapy among adult patients presenting for HIV treatment initiation or re-initiation in sub- Saharan Africa: a systematic review

Mariet Benade[a], Mhairi Maskew[b], Allison Juntunen[a], Sydney Rosen[a,b§]

[a]Department of Global Health, Boston University School of Public Health, Boston, MA, USA
[b]Health Economics and Epidemiology Research Office, Department of Internal Medicine, School of Clinical Medicine, Faculty of Health Sciences, University of the Witwatersrand, Johannesburg, South Africa

[§]Author for correspondence: Sydney Rosen, 801 Massachusetts Ave Rm 390, Boston, MA 02118, USA, sbrosen@bu.edu

MB: mbenade@bu.edu
MM: mmaskew@heroza.org
AJ: juntunen@bu.edu
SR: sbrosen@bu.edu

Running head: Proportion of non-naïve ART initiators in Africa

Word count: 4105 (main), 300 (abstract)

For peer review only

Abstract

Objectives

As countries have scaled up access to antiretroviral therapy (ART) for HIV, patient attrition rates of up to 30% annually have created a large pool of individuals who initiate treatment with prior ART experience (non-naïve re-initiators). Little is known about the proportion of non-naïve re-initiators within the population presenting for treatment initiation.

Design

We conducted a rapid review of published reports with data on proportions of adult patients initiating ART who were treatment non-naïve in sub-Saharan Africa. We searched PubMed, EMBASE, Web of Science, and international conferences for studies reported after 01/01/2018 with data collected after January 2016, when universal HIV treatment access was adopted. We included clinical trials and observational studies and accepted self-report, laboratory discernment of antiretroviral metabolites, or viral suppression at initiation or in the medical record as evidence of prior exposure. We report results of each eligible study and identify gaps in the literature.

Results

Of 1,782 articles from our initial search, we found nine, describing ten cohorts, that contained sufficient information for the review, half from South Africa. The proportion of the study samples with evidence of prior ART use ranged from 5% (self-report only) to 53% (presence of ART metabolites in hair or blood sample among self-reported naïve patients). The vast majority of screened studies did not report naïve/non-naïve status, and among the few that did, the metrics used to determine and report non-naïve proportions were inconsistent and difficult to interpret.

Conclusions

The proportion of patients initiating HIV treatment who are truly ART-naïve is not well documented in the literature. From the studies identified, it seems likely that 20% to 50% of ART patients who present for ART are re-initiators. Standard reporting metrics and diligence in reporting are needed, as is research to understand the reluctance of patients to report prior ART exposure.

Registration: Prospero CRD42022324136

Keywords: Antiretroviral therapy, Treatment naïve, Africa, Systematic review, Initiation, Re-initiation

Strengths and limitations of this study

- Comprehensive review using multiple databases and conference abstract archives.
- Focused on recent data in the era since access to antiretroviral therapy for HIV became universal, increasing the potential for re-inititation of treatment.
- Utilized best practices to develop search string, with multiple reiterations based on initial findings.
- Recognized a wide range of reporting indicators for prior exposure to ARVs.
- Limited by the lack of standardized reporting of prior ARV exposure .

For peer review only - http://bmjopen.bmj.com/site/about/guidelines.xhtml

Introduction

The successful scale-up of access to antiretroviral therapy (ART) for HIV treatment in sub-Saharan Africa has produced a growing population of patients who have interrupted or stopped treatment sometime since they started, either permanently or temporarily. While very recent numbers on attrition from ART programs are scarce, retention in care rates for the region were reported to average 78% at 12 months after treatment initiation in a review published in 2015, suggesting that for a cohort of patients initiating in any given year, nearly a quarter have been lost from care one year later[1]. Many of these lost patients, however, proceed to "re-initiate" treatment in the months or years after dropping out of treatment programs[2]. Two estimates posit the extent of treatment re-initiation. The first, from the U.S. President's Emergency Plan for AIDS Relief (PEPFAR) reported that more than 580,000 patients returned to care after a treatment interruption in just the quarter from July to September 2020 in the countries that PEPFAR supports[3]. The second was from the Western Cape Province of South Africa, where among the subset of patients whose CD4 counts were less than 50 cells/mm^3, the proportion with prior treatment experience rose from 14% to 57% between 2008 and 2017[4].

Outside of indirect estimates such as those mentioned above, little is known about the actual proportions of non-naïve patients among all those presenting for ART initiation. Accurate data are difficult to obtain, largely because most HIV medical record systems neither distinguish between naïve and re-initiators nor allow tracking from one healthcare facility to another or over long intervals of inactivity. In most countries, a patient who originally initiated ART at one facility and then dropped out of care can easily present as a new patient at a nearby facility and be assumed to be ART-naïve. Self-reported information about a patient's naïve or non-naïve status may be unreliable, because patients who are known to have stopped treatment may be reprimanded, provided poorer service by healthcare facility staff, or required to participate in multiple adherence

For peer review only - http://bmjopen.bmj.com/site/about/guidelines.xhtml

training sessions, creating an incentive to present oneself as a new patient regardless of prior experience[5].

As the number of re-initiators continues to increase, understanding the proportion and characteristics of ART initiators who are not treatment-naïve is an important step in improving overall HIV treatment outcomes. By definition, treatment re-initiators previously faced barriers to retention in care that they were unable to overcome. Common barriers to retention include logistical challenges such as transport costs, psychosocial deterrents such as stigma, and personal preferences[6,7], and these barriers may become more prohibitive for patients who have already withdrawn from care once. Achieving long-term retention in care targets may thus require that healthcare systems differentiate interventions and services for re-initiators from those offered to naïve initiators. Re-initiators also comprise an increasing proportion of patients presenting with advanced HIV disease[4], who require additional care beyond simple ART initiation.

As national treatment programs mature, the proportion of ART initiators who are non-naïve will continue to grow, making the few available earlier estimates obsolete. To help fill the gap in empirical evidence on current proportions of naïve and non-naïve ART initiators, we conducted a comprehensive, rapid review of recently published or presented (2018 and later) peer-reviewed reports in sub-Saharan Africa that directly or indirectly presented data on re-initiation rates.

For peer review only

Methods

Following World Health Organization guidance for rapid reviews[8] we conducted a rapid systematic review of peer-reviewed publications and conference abstracts that reported on prior exposure to antiretroviral therapy among adult patients presenting for HIV treatment in sub-Saharan Africa. The

For peer review only - http://bmjopen.bmj.com/site/about/guidelines.xhtml

review was registered on the International Prospective Register of Systematic Reviews (PROSPERO CRD42022324136) (File S1).

Search strategy, study selection, and data extraction

For this review, our primary outcome was the proportion of adults presenting for ART initiation (initially or after interruption) in public sector HIV treatment programs in sub-Saharan Africa who are not ART naïve. To indicate prior ART use, we accepted self-reported questionnaire responses, rates of viral load suppression at ART initiation (suggesting previous ART use), medical record evidence (e.g. a prior viral load test), and biological measurements of the presence of antiretroviral metabolites in blood, hair, or urine specimens at or prior to treatment initiation. Inclusion and exclusion criteria for the review are shown in Table S1.

To identify potential sources, we developed a search string with the assistance of a medical librarian. The search syntax included variations of the terms HIV, treatment, antiretroviral therapy, retention, and adherence, limited to sub-Saharan Africa. During the course of the review, we made three revisions to the original protocol as submitted to PROSPERO, in order to maximize the potential for finding relevant sources. Specifically, we 1) included studies focusing on pregnant women starting ART in PMTCT programs; 2) added “undisclosed” and “retention” as search terms, as shown in S2 Table; and 3) included studies that required a minimum duration of ART for enrollment, as explained below. Search strings can be found in Table S2.

We searched the PubMed, Embase, and Web of Science databases and abstracts for the International AIDS Conference, International AIDS Society (IAS) Conference on HIV Science, and Conference on Retroviruses and Opportunistic Infections (CROI) with a search string developed to identify English-language publications which reported on HIV treatment initiation in sub-Saharan

For peer review only - http://bmjopen.bmj.com/site/about/guidelines.xhtml

Africa from 1 January 2018 until 31 March 2022. We further limited our search to articles from which the majority of the data were generated in 2016 or later, as this was when universal treatment access became common in the region. Follow-up searches extended the latest publication or conference presentation date of the review to September 15, 2022.

We included cohort, cross-sectional, case-control, and interventional studies that reported primary data on initiation of ART in the adult (≥18 years old) population, including for PMTCT. We included indexed pre-prints but excluded unpublished reports. We also excluded commentaries, modeling studies, and other sources that did not report primary data. While we excluded systematic reviews and meta-analyses, we manually searched existing systematic reviews for additional, non-duplicate references to be included. Where more than one publication reported on the same patient cohort, we chose the one that was either most recent or provided the most relevant data.

Because we were interested specifically in reports of the proportion of patients presenting for ART initiation or re-initiation in routine care who were naïve and non-naïve, we excluded studies that stated that prior ART experience was an exclusion criterion for the study, with one exception. If a study included only participants who self-reported as naïve but were then found to have evidence of previous ARV exposure, we included that proportion as a result, accepting that it explicitly omits patients who excluded themselves because of prior exposure and thus almost certainly reflects an underestimate of the true population prevalence of previous exposure. We also included studies that only enrolled patients who had achieved a specified duration of follow up on ART—whether one month or several years—despite the fact that these studies would have missed patients who were lost from care prior to reaching that specified duration.

All peer reviewed references identified using the respective search strings from PubMed, Embase and Web of Science were imported into Rayyan QCRI, where deduplication occurred. An initial,

For peer review only - http://bmjopen.bmj.com/site/about/guidelines.xhtml

independent, blinded review (reviewers were not aware of each other's decisions) of the titles and abstracts was conducted by three study team members (MB, AJ, SR) using Rayyan QCRI. A full text review was then conducted for all publications remaining after the initial review by two study team members (MB, AJ or MB, SR), with conflicts resolved through discussion. Reasons for excluding publications were recorded during the full text review. As a quality check, one author (SR) also checked a sample (10%) of the excluded sources against exclusion criteria. At each stage of the review process any conflicts between reviewers were assessed and resolved though consensus of three authors (MB, AJ, SR). The results of the search were documented in accordance with the PRISMA-P reporting checklist (File S2).

We created a data extraction tool to capture study and sample characteristics, proportions with previous ART exposure, and the type of indicator of previous exposure reported (e.g. self-report, laboratory results).

Outcomes and analysis

Our outcome of interest was the proportion of ART initiates who were treatment naïve at ART initiation, defined as a patient presenting for initiation of ART who has never previously taken antiretroviral therapy for treatment of HIV ("new initiator"), compared the proportion who were treatment-experienced at ART initiation, defined as a patient presenting for initiation of ART who had previously taken antiretroviral therapy for HIV treatment but had interrupted that therapy for a minimum of 3 months ("re-initiator"). We accepted each paper's source of information about participants' status: self-report, medical record review, viral suppression, or laboratory tests for ARV metabolites and report that source in our results.

For peer review only - http://bmjopen.bmj.com/site/about/guidelines.xhtml

To evaluate the data, we first report each paper's outcome, with descriptive information regarding the population and setting to which the results apply. As described in the Prospero protocol, we had intended to estimate pooled results for individual countries and populations and to stratify by patient and facility characteristics, but we ultimately identified too few eligible sources to allow for any pooled or stratified analysis.

Quality of the eligible studies was assessed using the Joanna Briggs Institute Critical Appraisal Checklists[9].

Patient and public involvement

There was no patient or public involvement in this paper.

Results

Sources identified

The results of the systematic search are shown in Figure 1. A total of 1,782 non-duplicate abstracts of peer reviewed journal articles and 9 abstracts from the selected conferences were screened. After the initial title and abstract review, 1,450 articles and abstracts were excluded, leaving 332 documents for full-text review. During the full review, an additional 322 were excluded. Reasons for exclusions are reported in Table S3. The primary reason for exclusion was lack of information on the naïve or non-naïve status of participants.

Figure 1. PRISMA flow chart

For peer review only

For peer review only - http://bmjopen.bmj.com/site/about/guidelines.xhtml

Nine peer-reviewed articles were retained in the final data set for the full review, including one that reported data from two countries and will be included in our analysis as two studies, creating a total of ten sets of results. The studies are described in Table 1.

Table 1. Studies included in the review

Source (alphabetical by country)	Country and location	Study design and source of data	Study population	Dates of presentation for ART initiation	Sample size	Sex (% female)	Age (median, IQR)
Lebelonyane 2020[10]	Botswana (national sample)	Subset of baseline data for intervention arm of cluster randomized trial of HIV prevention	Adults found to be HIV-positive during community HIV prevention campaign and linked to HIV care	June 2016-March 2018	800	55%	33 (26-41)
Buju 2022[11]	Democratic Republic of Congo (Bunia)	Observational prospective cohort of patients receiving dolutegravir	Adults presenting for ART initiation or ongoing ART treatment at all ART clinics in Busia	July 2019-July 2021 for those initiating during the study; earlier for those already on ART	177	69%	39 (12)*
Genet 2021 [12]	Ethiopia (Northwest Ethiopia)	Observational, cross sectional study of first line treatment failure	Adults† who had received at least 6 months of ART	May October 2017	430	58%	38 (12-67)**
Kunzweiler 2018 [13]	Kenya (Kisumu)	Observational prospective cohort	Adult men who have sex with men (MSM)	August 2015-September 2016	63	0%	27 (22-32)
Rosen 2019[14]	Kenya (Kericho, Kapsabet, and Kombewa counties)	Baseline data for intervention arm of clinical trial of same-day ART initiation	Adults presenting for ART initiation at 3 public sector hospitals	July 2017-April 2018	477	58%	36 (29-44)
Dorward 2020[15]	South Africa (KwaZulu Natal Province)	Baseline data for randomized controlled trial for point-of-care HIV viral load testing	Adults clinically stable on ART and due for 6-month viral load testing	August 2016-February 2017 (est)	390	60%	32 (27-38)
Maskew 2020[16]	South Africa (Gauteng Province)	Baseline data for intervention arm in clinical trial of same-day ART initiation	Adults presenting for ART initiation at 3 public sector clinics	March-September 2018	296	64%	35 (30-44)
Mavhandu-Ramarumo 2019[17]	South Africa	Baseline samples from	Adults presenting for ART initiation at	2017-2019	77	90%	35 (27-42)

For peer review only - http://bmjopen.bmj.com/site/about/guidelines.xhtml

Source (alphabetical by country)	Country and location	Study design and source of data	Study population	Dates of presentation for ART initiation	Sample size	Sex (% female)	Age (median, IQR)
	(Limpopo Province)	clinical trial of drug resistance	3 public sector clinics				
Rosen 2019 [14]	South Africa (Gauteng Province)	Baseline data for intervention arm of clinical trial of same-day ART initiation	Adults presenting for ART initiation at 3 public sector clinics	March-July 2017	600	63%	34 (29-41)
Sithole 2021[18]	South Africa (KwaZulu Natal Province)	Subset of baseline data from clinical trial of home-based ART initiation	Non-pregnant adults presenting for ART initiation at 2 public sector clinics, with CD4 >100 and no active TB	February 2018-November 2018	193	60%	Not reported

*Mean (standard deviation)
†Study included children under 18, but they comprised <7% of the study sample
**Mean (minimum-maximum)

The first five studies in Table 1 were conducted in Botswana, the Democratic Republic of Congo (DRC), Ethiopia and Kenya, and the remaining five all in South Africa. Seven of the ten reported baseline data from a clinical trial conducted for other purposes, while three were observational studies. All were very or somewhat small in size, enrolled adult patients presenting or having previously presented for routine ART initiation at public sector clinics, and collected most or all data prior to the disruption in service delivery caused by COVID-19 in early 2020[19]. Specific populations enrolled varied by study, though only one, Kunzweiler 2018, was limited to a non-general adult population.

Proportions of patients non-naïve

In Table 2, we report the proportion of patients in each study reported to be non-naïve when presenting for ART initiation.

Table 2. Proportions of cohorts reported to be non-naïve at ART initiation

For peer review only

Source	Source of data	Proportion non-naïve at initiation	Inclusion criteria related to prior ART exposure	Comments
Lebelonyane 2020*	Not stated; only indicated as participants who had “previous ART treatment”	54/800 (7%)	Sample included 16-17 year olds; minors may be less likely than adults to have had an opportunity for prior ART exposure.	Participants were identified through community-based testing and referred for ART; sample does not represent routine walk-in ART initiation population.
Buju 2022	Viral load suppressed at ART initiation	93/177 (52%)	None.	Enrolled both initiators patients and patients already on ART; results presented are only for initiators.
Genet 2021	Self-report	90/430 (21%)	Sample included 12-17 year olds; minors may be less likely than adults to have had an opportunity for prior ART exposure. Participants required to have completed 6 months on ART; patients lost to follow up before reaching 6 months were excluded.	Enrolled patients younger than 18. Those on second line ART were excluded; patients eligible for second line treatment may be more likely to be re-initiators.
Kunzweiler 2018	Viral load suppressed at ART initiation	19/63 (30%) (13 of 19 with suppressed viral load at ART initiation self-reported being ART-naïve)	Excluded 9 participants who were lost to follow up before reaching 6 months on ART.	Also excluded 3 patients who did not have viral load test results.
Rosen 2019	Self-report	18/240 (8%)	Included self-reported re-initiators who had interrupted ART for ≥ 3 months.	Patients who self-reported that they had previously initiated ART but had stopped for ≥3 months were eligible; those who self-reported that they had interrupted for < 3 months were excluded.
Dorward 2020	Self-report	18/390 (5%)	Participants required to have completed 6 months on ART; patients lost to follow up before reaching 6 months were excluded.	
Maskew 2020	Self-report	33/296 (11%)	Included self-reported re-initiators who had interrupted ART for ≥ 3 months	Patients who self-reported that they had previously initiated ART but had stopped for ≥3 months were eligible; those who self-reported that they had interrupted for < 3 months were excluded.
Mavhandu-Ramarumo 2019	Laboratory assay of blood and/or hair sample for presence of TDF, EFV, and/or FTC metabolites	41/77 (53%)	All participants self-reported as ART naïve	Stated enrollment criteria included naivete; those who had been on ART previously may have self-screened out of this study.
Rosen 2019	Self-report	7/298 (2%)	Included self-reported re-initiators who had interrupted ART for ≥ 3 months	Patients who self-reported that they had previously initiated ART but had stopped for ≥3 months were eligible; those who self-

For peer review only - http://bmjopen.bmj.com/site/about/guidelines.xhtml

Source	Source of data	Proportion non-naïve at initiation	Inclusion criteria related to prior ART exposure	Comments
				reported that they had interrupted for < 3 months were excluded.
Sithole 2021†	Undetectable viral load at ART initiation	62/193 (32%) (Other outcomes reported: 42/193 (22%) had medical record evidence of prior ART use; 37/193 (19%) had detectable ARV metabolites in blood.)	All participants self-reported as ART naïve	Stated enrollment criteria included naivete; those who had been on ART previously may have self-screened out of this study. Participants were identified through community-based testing; sample does not represent routine walk-in ART initiation population. Primary DO-ART trial excluded 588 participants out of 2,479 who were virally suppressed at time of initiation and 66 who were found to already be on ART[20]

TDF, Tenofovir; EFV, Efavirenz; FTC, Emtricitabine
*A separate publication by the same author team, reporting data that overlapped with those reported in this study, stated that "22% of advanced HIV patients had previously been on ART." This sample was limited to those with AHD, however.
†The parent study on which this study was based.

The proportion of patients presenting for ART initiation who were reported to be non-naïve ranged from 2 to 53%. It is important to note that the results shown in Table 2 are not strictly comparable to one another, however, due to the different data sources, populations, and relevant exclusions listed in the right-hand column of the table. In Maskew 2021 and Rosen 2020, non-naïve patients were allowed to enroll as long as they had been off ART for at least three months. Nevertheless, these studies, which are the only ones that relied entirely on participant self-report, reported the lowest proportion of non-naïve participants. Two other studies in Table 2, Mavhandu-Ramarumo 2019 and Sithole 2021, explicitly excluded self-reported non-naïve patients; all patients enrolled in these studies claimed not to have been on ART previously. The relatively high proportions of non-naïve patients in these studies are thus still minimum estimates of the true proportion of ART initiators with prior treatment exposure at the study clinics, as anyone who admitted to prior use of ARVs will have been excluded from study enrolment. Similarly, Kunzweiler 2018 and Dorward 2020 excluded patients lost to follow up before 6 months after ART initiation, and thus may have underestimated the proportion re-initiating if non-naïve patients are more likely than naïve patients to drop out of care. Study participants were also drawn from diverse populations of individuals with HIV, including those identified in a community campaign (Lebelonyane 2020), in a household survey as part of a

For peer review only

randomized trial (Sithole 2021), or as routine, walk-in presenters at clinics (Buju 2022, Maskew 2020, Rosen 2019).

Because all the eligible studies were small and/or had primary outcomes other than the proportion naive, little stratification of results by facility or patient characteristics was reported. Mavhandu-Ramarumo 2019 reported that 7 of the 8 (88%) males in the sample population had evidence of prior ART exposure, compared to only 49% of the 34 females. In contrast, Sithole 2021 found that women (37%) were more likely to have evidence of undisclosed ART use than men (25%). Other characteristics that were associated with undisclosed ART use by undetectable viral load were younger age (35% in 18-29 year olds, 30% in 30-49 year olds, and 23% among those >50 years) and living with a partner who was HIV positive (44% compared to 37%; adj OR 1.94 (95% CI 0.95-3.96)). Kunzweiler 2018's results apply specifically to MSM. Other studies did not provide information for stratification by facility or patient characteristics.

Quality of evidence

Each of the studies included in the review either presented baseline enrollment data from a randomized controlled trial or observational data. Outcomes after ART initiation were not relevant for our review, which looked only at the status of patients at baseline (ART initiation). For Maskew 2020 and Rosen 2019, data on self-reported naïve status were limited to the intervention arm. The setting, inclusion criteria, and baseline characteristics for these studies were clearly defined. Mavhandu-Ramarumo 2019, Lebelonyane 2020, Dorward 2020, and Sithole 2021 also sufficiently defined inclusion criteria, study setting, and baseline characteristics.

We assessed Kunzweiler 2018, Genet 2021, and Buju 2022 as observational studies. Kunzweiler 2018 utilised snowball sampling, as is frequently done among key populations at high risk of stigma. All

For peer review only - http://bmjopen.bmj.com/site/about/guidelines.xhtml

three papers described the study setting and sample population of interest clearly, including age, sex and clinical characteristics of HIV presentation.

As is indicated in Table 2 and discussed further below, each of the studies included in this review used a different indicator of prior ART exposure (non-naivete), and most had limitations as to their accuracy and/or the representativeness of their populations.

Discussion

We systematically reviewed peer-reviewed evidence on the proportions of patients presenting for treatment initiation in sub-Saharan Africa who are or are not ART-naïve. The proportions non-naïve in the sources we found ranged from a low of 2% using self-report only to a high of 53% based on a laboratory analysis of ARV metabolites in blood and hair samples of patients who self-reported to be naïve.

Perhaps the most striking finding of this review is the sheer lack of published evidence to answer our research question. Despite a comprehensive search of the literature published between 2018 and 2022, and including data since 2016, we identified only nine sources and ten cohorts that reported this information, and most included it in only in passing. Half the studies were conducted in South Africa and were relatively small in size; those from other countries provided very little detail. Based on the published and presented research alone, it is fair to say that very little is known about the true proportion of ART initiators who are not treatment-naïve in South Africa, and almost nothing is known about the rest of the region or about specific sub-populations or risk groups. While it is possible that more information is available to program managers who have access to routinely collected medical record data, nothing in the literature suggests that such information is being generated on a large scale or, more important, utilized for program improvement.

For peer review only - http://bmjopen.bmj.com/site/about/guidelines.xhtml

During our search, we made a concerted effort to find additional eligible sources, in the hope that there would be more data to review and analyze. This included adding additional search terms, including data on pregnant and post-partum women, and reviewing reference lists from relevant systematic reviews. We reviewed an unusually large number of full-text manuscripts in the hope that they would include proportions naïve and non-naïve in their cohort descriptions (typically "Table 1") even though there was no indication of this in the abstract.

Unfortunately, most of the sources that originally appeared promising were found to be ineligible, for various reasons. Most simply did not report on baseline naïve/non-naïve status at ART initiation, using any indicator. Some intentionally excluded non-naïve patients prior to enrolment and then reported all participants as naïve without further investigation. For studies that explicitly screened out non-naïve participants, we considered calculating proportions non-naive based on reported numbers of potential participants included and excluded, but we realized that many studies only applied the non-naïve criterion after screening potential participants out for other reasons. We thus could not safely rely on numbers screened out due to non-naïve status for our numerator and had to exclude those papers. Even some of the papers we did include either provided only a passing reference about prior ART exposure, making us uncertain that we interpreted them correctly (e.g. Lebelonyane 2020), or explicitly excluded patients lost to follow up in the early treatment period (e.g. Kunzweiler 2018 and Dorward 2020).

The small number of eligible papers we did find offer some useful information. They used several different indicators for identifying non-naïve patients, and the indicators produced results that are consistent with their expected accuracy. Maskew 2020 and Rosen 2019, which relied solely on self-report, and Lebelonyane 2020, for with the source of data is unknown, found the lowest proportions non-naïve. Sithole 2021, which excluded a priori anyone self-reporting prior utilization, reported that

For peer review only

32% of patients had evidence of prior use based on being virally suppressed. Buju 2022 and Kunzweiler 2018 reported that 52% and 30%, respectively, of patients presenting for initiation already had suppressed viral loads, suggesting prior ARV use. Mavhandu-Ramarumo 2019, which also excluded a priori anyone admitting prior utilization, using the most rigorous methodology with both blood and hair samples, estimated 53% of patients had prior ART exposure. The large observed difference between males and females and the small number of males in this study, however, suggest caution in applying the results to male patients.

Based on results of the studies that used laboratory tests, we assume that those that relied on self-report—e.g. Maskew 2020, Rosen 2019, and Dorward 2020—underestimated the true proportion of participants who were non-naïve at initiation. While study inclusion criteria may have biased the samples in Sithole 2021 and Mavhandu-Ramarumo 2019, these, together with Buju 2022, Kunzweiler 2018, and Genet 2021, suggest that it is reasonable (and conservative) to conclude that between 20% and 50% of ART patients—and likely at least 30%--who present for ART are re-initiators. This proportion can be expected to increase with each passing year, as the number of truly naïve HIV-positive individuals declines. If this is so, then re-initiators comprise an important sub-population whose needs are likely to differ from those of naïve initiators and to whom service delivery should be tailored.

As is evident from the discussion above, this review had several limitations. First, while we believe that our search of the peer-reviewed, published literature and abstracts was thorough, the lack of standard terminology for describing prior ART exposure hampered the creation of precise search strings, and it is possible that some sources were missed. Second, we found information from only five of sub-Saharan Africa's 46 countries, and five of our ten observations were from a single country, South Africa. Since each country in sub-Saharan Africa has a different experience with attrition from ART and approach to re-initiation, results may not be generalizable. Third, as

For peer review only - http://bmjopen.bmj.com/site/about/guidelines.xhtml

explained in the introduction, even such data as are available tend to be incomplete, due to the limitations of self-reporting and of existing medical record systems.

Fourth, the wide range of results identified may reflect study methodologies, but it may also indicate substantial geographic diversity in outcomes that we cannot address with the data available. We speculate that additional relevant data are collected by program managers, ministries of health, and others but are either not analyzed to answer our research question or simply not published and therefore not accessible. Fifth, the small number of eligible sources, small sample sizes, and heterogeneity of research methods made it impossible to aggregate the results or produce meaningful summary statistics, beyond the range discussed above. Sixth, recall bias may be present in the studies that asked participants after six months or more on ART to self-report their naïve/non-naïve status at the time they initiated treatment. Finally, the fact that six of ten cohorts included were from clinical trials that provided patient compensation may have biased enrollments, though we cannot know how this may have affected patient enrolment or self-report of prior ART usage and the subsequent direction of the bias.

In addition to the sheer dearth of information available to answer our question, the search reported here revealed three important research priorities. First, there is a need for a standard terminology to describe patients with prior ART exposure and prior ART initiation experience. Even the binary terms "naïve" and "non-naïve" can be unclear if patients have previously used antiretroviral medications for PrEP or prevention of vertical transmission. Terms such as "ART experienced" and "ART exposed" are often substituted for non-naïve, without specification of what they refer to. Prior ART use may be "disclosed" or "self-reported." Similarly, the duration of treatment interruption that leads to "re-initiation" is rarely specified, and "re-initiation" and "re-engagement" are used interchangeably. We can assume that patient returning to care after an interruption of less than one month is not likely to be regarded as a re-initiator, and a patient returning after an interruption of more than one year will

For peer review only

likely be defined as a re-initiator. But what of patients with interruptions of 6 or 8 months? A common terminology for describing the phenomenon addressed here would be of great assistance in understanding its magnitude.

Second, in view of the potentially very high proportion of re-initiators among "new" ART patients, it is critical that researchers begin to report proportions naïve and non-naïve as a standard variable when describing patient cohorts, even if data come solely from self-report. We cannot determine from the literature whether many studies do collect this information but omit it from their reports or if it has simply not been collected. We identified several papers that came close to indicating a proportion non-naïve but did not explain their findings clearly enough to include in this review, suggesting that many studies do indeed have access to the relevant information. The study mentioned in our introduction from South Africa's Western Cape Province, for example, provided detailed information about patients with advanced HIV disease and suggested that more than a third of all patients with very low CD4 cell counts had previously been on ART but were now off, but we could not calculate the overall proportion of non-naïve initiators from the data reported [4]. In earlier years, when the proportion of non-naïve patients was low because treatment programs were still rapidly expanding, the question of prior ART experience may not have been a priority. In view of the results of the few studies available, it is clearly a priority now.

Finally, the phenomenon of large numbers of patients who decline to reveal prior ART use, even when asked directly, is concerning. For studies that intend to limit participation to naïve patients, stated exclusion of those who are non-naïve may encourage non-disclosure, to avoid being denied study enrolment on this basis. Even so, it appears likely that many patients opt to lie about prior exposure. Other research suggests that they have good reason for doing so, as clinics may refuse to re-initiate those who admit to prior default and/or may provide poorer service to them[21–23].

For peer review only - http://bmjopen.bmj.com/site/about/guidelines.xhtml

Creating a clinic and community atmosphere that promotes honesty about prior exposure should also be a priority.

In conclusion, while we recognize that simply knowing the proportion of non-naïve patients in a given population will not in itself improve the quality of service delivery, measuring the size of the problem is a critical step in creating momentum to development and implement interventions targeted specifically at re-initiators. Since these are patients who have already demonstrated that they face obstacles to remaining in care, identifying and targeting them for appropriate services is a vital step in improving the outcomes of treatment programs.

Supplemental files

S1 File. Search protocol (Prospero)

S2 File. PRISMA checklists for publications and abstracts

S1 Table. Inclusion/exclusion criteria for publications and abstracts

S2 Table. Search strategy

S3 Table. Reasons for exclusion after full-text review

Ethics approval and consent to participate

Not applicable; this is a systematic review of published information and has no human participants.

Availability of data and materials

All data used in this paper are included in the manuscript and publicly available in the cited sources.

Competing interests

The authors declare that they have no competing interests.

Funding

Funding for the study was provided the Bill & Melinda Gates Foundation through INV-031690 to Boston University. The funder had no role in study design, data collection and analysis, decision to publish, or preparation of the manuscript.

For peer review only

For peer review only - http://bmjopen.bmj.com/site/about/guidelines.xhtml

Author contributions

MM, SR, and MB conceived of and designed the study. MB, SR, and AJ identified and reviewed sources and extracted data. MB and SR analyzed the data and drafted the manuscript. All authors reviewed and edited the manuscript.

Acknowledgements

None

References

1. Fox MP, Rosen S. Retention of adult patients on antiretroviral therapy in low- and middle-income countries: systematic review and meta-analysis 2008–2013. J Acquir Immune Defic Syndr 2015;69: 98. doi:10.1097/QAI.0000000000000553
2. Ehrenkranz PD, Rosen S, Boulle A, Eaton J, Ford N, Fox M, et al. The revolving door of HIV care : Revising the service delivery cascade to achieve the UNAIDS 95-95-95 goals. PLoS Med 2021;18: e1003651. doi:10.1371/journal.pmed.1003651
3. Godfrey K. Supporting reengagement: What's new in DSD for HIV treatment: from WHO recommendations to reality. IAS 2021, July 18-21, 2021. Available from: https://differentiatedservicedelivery.org/Portals/0/adam/Content/TlGQJAsI5UqL5SHACJkSPg/File/IAS2021_treatmentcontinuity_IAS%20inputs%2030June.pdf. Accessed 19 Oct 2022.
4. Osler M, Hilderbrand K, Goemaere E, Ford N, Smith M, Meintjes G, et al. The continuing burden of advanced HIV disease over 10 years of increasing antiretroviral therapy coverage in South Africa. Clin Infect Dis 2018;66: S118–S125. doi:10.1093/cid/cix1140
5. Bisnauth MA, Davies N, Monareng S, Buthelezi F, Struthers H, McIntyre J, et al. Why do patients interrupt and return to antiretroviral therapy? Retention in HIV care from the patient's perspective in Johannesburg, South Africa. PLoS One 2021;16: 1–15. doi:10.1371/journal.pone.0256540
6. Mody A, Sikombe K, Beres LK, Simbeza S, Mukamba N, Eshun-Wilson I, et al. Profiles of HIV care disruptions among adult patients lost to follow-up in Zambia. J Acq Immun Defic Syndr 2021; 86: 62–72. doi:10.1097/qai.0000000000002530
7. Sikazwe I, Eshun-Wilson I, Sikombe K, Beres LK, Somwe P, Mody A, et al. Patient-reported reasons for stopping care or switching clinics in Zambia: A multisite, regionally representative estimate using a multistage sampling-based approach in Zambia. Clin Infect Dis 2021;73: e2294–e2302. doi:10.1093/cid/ciaa1501
8. Stevens A, Garritty C, Hersi M, Moher D. Developing PRISMA-RR, a reporting guideline for rapid reviews of primary studies (Protocol). Available from: http://www.equator-network.org/wp-content/uploads/2018/02/PRISMA-RR-protocol.pdf. Accessed 19 Oct 2022.
9. Joanna Briggs Institute. Critical Appraisal Tools. Available from : https://jbi.global/critical-appraisal-tools. Accessed 6 Sep 2022.
10. Lebelonyane R, Bachanas P, Block L, Ussery F, Abrams W, Roland M, et al. Rapid antiretroviral therapy initiation in the Botswana Combination Prevention Project: a quasi-experimental before and after study. Lancet HIV 2020;7: e545–e553. doi:10.1016/S2352-3018(20)30187-9
11. Buju RT, Akilimali PZ, Tran N-T, Kamangu EN, Mesia GK, Kayembe JMN, et al. Determinants of survival of HIV patients receiving dolutegravir: a prospective cohort study in conflict-affected Bunia, Democratic Republic of Congo. Int J Environ Res Public Health 2022;19. doi:10.3390/ijerph191610220
12. Genet A, Mekonnen Z, Yizengaw E, Mekonnen D. First line antiretroviral treatment failure and associated factors among people living with HIV in northwest Ethiopia. Afr Health Sci 2021;21: 263–272. Available: https://pubmed.ncbi.nlm.nih.gov/34394306/

For peer review only

For peer review only - http://bmjopen.bmj.com/site/about/guidelines.xhtml

13. Kunzweiler CP, Bailey RC, Mehta SD, Okall DO, Obondi E, Djomand G, et al. Factors associated with viral suppression among HIV-positive Kenyan gay and bisexual men who have sex with men. AIDS Care 2018;30: S76–S88. doi:10.1080/09540121.2018.1510109
14. Rosen S, Maskew M, Larson BA, Brennan AT, Tsikhutsu I, Fox MP, et al. Simplified clinical algorithm for identifying patients eligible for same-day HIV treatment initiation (SLATE): Results from an individually randomized trial in South Africa and Kenya. PLoS Med 2019;16: e1002912. doi:10.1371/journal.pmed.1002912
15. Dorward J, PK D, Osman F, Sookrajh Y, Pillay M, Moodley P, et al. Short communication: early antiretroviral therapy is associated with better viral suppression and less HIV drug resistance after implementation of universal treatment in South Africa. AIDS Res Hum Retroviruses 2020;36: 297–299. Available: https://pubmed.ncbi.nlm.nih.gov/31663368/
16. Maskew M, Brennan AT, Fox MP, Vezi L, Venter WDF, Ehrenkranz P, et al. A clinical algorithm for same-day HIV treatment initiation in settings with high TB symptom prevalence in South Africa: The SLATE II individually randomized clinical trial. PLoS Med 17(8): e1003226. https://doi.org/10.1371/journal.pmed.1003226
17. Mavhandu-Ramarumo LG, Tambe LAM, Matume ND, Katerere D, Bessong PO. Undisclosed exposure to antiretrovirals prior to treatment initiation: An exploratory analysis. South Afr J HIV Med 2021;22: 1–10. doi:10.4102/sajhivmed.v22i1.1200
18. Sithole N, Gunda R, Koole O, Krows M, Schaafsma T, Moshabela M, et al. Undisclosed antiretroviral therapy use at primary health care clinics in rural KwaZulu Natal South Africa: A DO-ART trial sub-study. AIDS Behav 2021. doi:10.1007/s10461-021-03319-4
19. Benade M, Long L, Rosen S, Tucker J. Reduction in initiations of HIV treatment in South Africa during the COVID pandemic. BMC Health Serv Res 2022;22: 428. doi:10.1186/s12913-022-07714-y
20. Barnabas R, AA S, H van R, Asiimwe S, Pillay D, NC W, et al. Community-based antiretroviral therapy versus standard clinic-based services for HIV in South Africa and Uganda (DO ART): a randomised trial. Lancet Glob Health 2020;8: e1305–e1315. doi:10.1016/S2214-109X(20)30313-2
21. Bisnauth MA, Davies N, Monareng S, Buthelezi F, Struthers H, McIntyre J, et al. Why do patients interrupt and return to antiretroviral therapy? Retention in HIV care from the patient's perspective in Johannesburg, South Africa. PLoS ONE 2021; 16:e0256540. doi:10.1371/journal.pone.0256540
22. Rees K, Bisnauth M, O'Connor C, Ramvhulela T, Vali N. Understanding patients reinitiating antiretroviral therapy in two South African districts. South Afr J HIV Med 2022;23. doi:10.4102/sajhivmed.v23i1.1380
23. Bisnauth MA, Davies N, Monareng S, Struthers H, McIntyre JA, Rees K. Exploring healthcare workers' experiences of managing patients returning to HIV care in Johannesburg, South Africa. Glob Health Action 2022;15. doi:10.1080/16549716.2021.2012019.

For peer review only

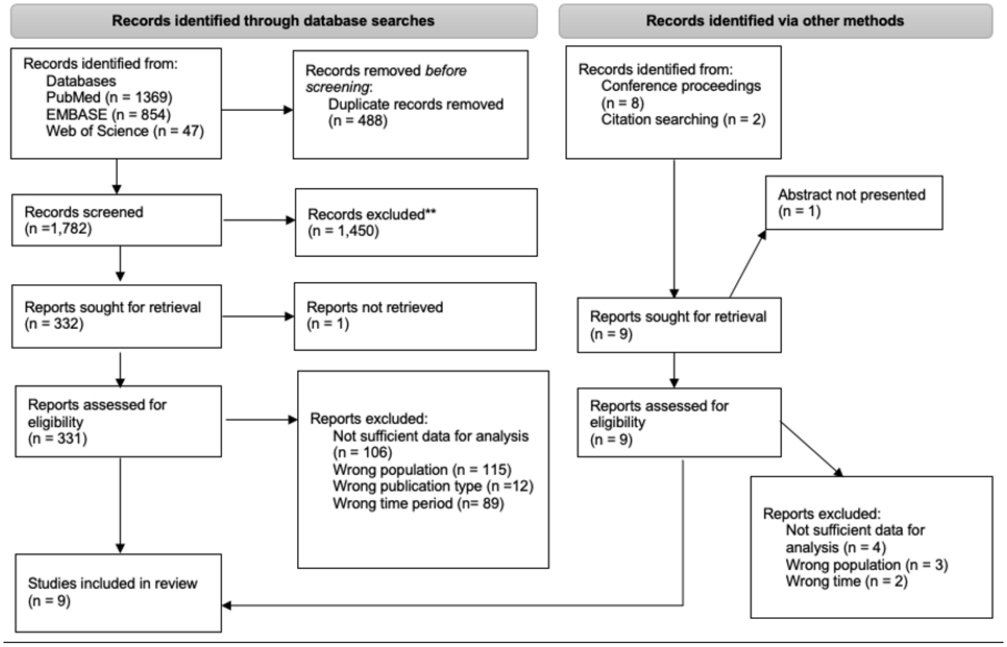

PRISMA flow chart

159x103mm (144 x 144 DPI)

NIHR | National Institute for Health Research

PROSPERO
International prospective register of systematic reviews

To enable PROSPERO to focus on COVID-19 submissions, this registration record has undergone basic automated checks for eligibility and is published exactly as submitted. PROSPERO has never provided peer review, and usual checking by the PROSPERO team does not endorse content. Therefore, automatically published records should be treated as any other PROSPERO registration. Further detail is provided here.

Citation

Mariet Benade, Mhairi Maskew, Linda Sande, Nancy Scott, Allison Juntunen, Sydney Rosen. Prior exposure to antiretroviral therapy among adult patients presenting for HIV treatment initiation or re-initiation in sub-Saharan Africa: a systematic review. PROSPERO 2022 CRD42022324136 Available from: https://www.crd.york.ac.uk/prospero/display_record.php?ID=CRD42022324136

Review question

What proportions of adult patients presenting for ART initiation or re-initiation in public sector HIV treatment programs in Southern Africa are (a) ART naïve (new initiators) and (b) treatment experienced (re-initiators)?

Searches

We will search PubMed, Embase, and Web of Science and abstracts from the International AIDS Conference, International AIDS Society (IAS) Conference on HIV Science, and Conference on Retroviruses and Opportunistic Infections (CROI). We will manually search reference lists from sources identified in the search. Unpublished studies (e.g. preprints) will be included. Searches will be limited to English language publications.

The search period will be from January 1, 2018 to March 31, 2022. Inclusion in the review will be limited to sources for which a majority of individual-level data pertain to 2016 or later, as 2016 was the year when universal treatment access became common in sub-Saharan Africa. If sources report data from both before and after January 1, 2016, only results pertaining to the data from after that date will be used.

The search will be re-run prior to final analysis to identify very recent reports.

Types of study to be included

Studies that report initiation of ART for adult patients. These can be cohort, case control, cross-sectional, or interventional studies. White papers, commentaries, modeling studies, cost-effectiveness studies, and case studies will be excluded. Qualitative studies will be excluded unless there is specific data on treatment experience at ART initiation. Existing systematic reviews will be evaluated for additional, non-duplicate references. Published protocols with no data will be excluded, but if the study meets the inclusion criteria, we will attempt to find study results to evaluate for inclusion.

Condition or domain being studied

The successful scale-up of access to ART for HIV in sub-Saharan Africa has produced a growing population of patients who have interrupted or stopped treatment, and are now “re-initiating” therapy. While a diminishing number utilized ART temporarily for prevention of mother-to-child transmission prior to universal eligibility for ART, most are patients who stopped treatment, despite guidelines recommending lifelong use. Estimates of the proportion of patients presenting for ART re-initiation range from 10% to 50%.

Treatment re-initiators previously faced barriers to retention in care that they were unable to overcome. Achieving long-term retention in care targets may require that healthcare systems differentiate interventions and services for re-initiators from those offered to naïve initiators.

A first step in improving retention in care for ART re-initiators is to estimate how many individuals there are in any population of patients presenting for ART initiation. Few such estimates are available, largely because most healthcare data systems do not distinguish between naïve and re-initiators, allow tracking from one

healthcare facility to another or over long intervals of inactivity. To provide a baseline for considering this challenge, we'll search the recent literature for estimates of the proportion of patients who are re-initiators in sub-Saharan Africa.

Participants/population

Inclusion: Adults (?18 years of age) living with HIV presenting for initiation of any regimen of lifelong antiretroviral treatment in any sub-Saharan African country.
Exclusion: Individuals presenting for initiation of antiretroviral medications for any reason other than therapeutic (e.g. PrEP, PMTCT).

Intervention(s), exposure(s)

Initiation of ART is the intervention of interest. This will be an observational study of the characteristics of individuals initiating ART for HIV treatment (specifically, whether or not they have prior exposure to ART at the time of initiation).

Comparator(s)/control

Not applicable. Eligible studies need only report the proportions of patients who are treatment naïve and those who are treatment experienced.

Context

Initiation of ART in routine care settings in sub-Saharan Africa, including any type of facility or provider.

Main outcome(s)

Proportion of patients who are:
a. Treatment naïve at ART initiation, defined as a patient presenting for initiation of ART who has never previously taken antiretroviral therapy for treatment of HIV (new initiator); or
b. Treatment experienced at ART initiation, defined as a patient presenting for initiation of ART who has previously taken antiretroviral therapy for HIV treatment but has interrupted that therapy for a minimum of 3 months (re-initiator).

Measures of effect

Proportion, odds ratio

Additional outcome(s)

If data allow, we will also estimate the primary outcomes stratified by patient and/or facility characteristics, such as patient age and sex and facility country and setting.

Measures of effect

Proportion

Data extraction (selection and coding)

Study selection:

Rayyan will be used to support screening of studies and track inclusion or exclusion of studies. References will be managed in Mendeley, and duplicates will be removed. Two independent reviewers will be involved in each phase of review, including title and abstract screening, full-text screening, and data extraction. Title and abstract screening will include a pilot test to assess if the reviewers are able to achieve over 90% agreement on inclusion based on the eligibility criteria listed in this protocol. Disagreements will be resolved through discussion to achieve consensus. After full text screening, relevant non-duplicate citations from previous systematic reviews and other studies labelled for snowball in the initial search will be added for inclusion/exclusion consideration in a snowball phase.

For peer review only

Data extraction:

Data extraction will use a pre-determined template that captures all relevant variables. Data extraction will be done independently by two reviewers and discrepancies will be resolved by consensus. Data items to be extracted from studies include:

- Country
- District or locality
- Facility type
- Facility setting (urban, rural)
- Study year(s) or period(s) cohort observed
- Length of patient follow-up
- ART guidelines or protocol(s) in use during study period
- Study/cohort sample size
- Patient characteristics, including:
o Age distribution
o Gender distribution
o Clinical indicators of disease stage such as presenting CD4 count or WHO stage
o Other co-morbidities
- Study design (e.g., cohort, case control, cross-sectional, interventional)
- Descriptions of routine care, including whether the study was designed to influence retention
- Prior treatment exposure
o Proportion of adult patients ART treatment-naïve at initiation of ART
o Proportion of adult patients ART treatment-experienced at initiation of ART
o Basis for report of prior exposure (patient self-report, review of medical or laboratory records, tests of biological samples, pre-existing viral suppression, other)
- Authors' observations or comments on proportion of patients naïve or non-naïve, if any

Risk of bias (quality) assessment

Risk of bias and quality of individual studies that are included in this systematic review will be evaluated with the Joanna Briggs Institute critical appraisal checklist most relevant to the study type, as we expect that most included studies will be observational. The most important characteristics to be assessed will be recruitment biases (whether the study enrolled a representative sample of the specified population) and accuracy of naïve/non-naïve status reporting. Two reviewers will be involved in quality assessment, with disagreements resolved by a third reviewer on the study team.
We note that we expect that most studies meeting inclusion criteria for this review will report our outcomes (proportions naïve and non-naïve) as secondary outcomes or in their descriptions of their study cohorts, rather than as primary outcomes. (For example, a clinical trial with a primary outcome of viral suppression at 12 months after treatment initiation may report the proportion of participants who were naïve at initiation; our review would include the proportion naïve but not the primary outcome of viral suppression.)

Strategy for data synthesis

We will first present raw results from all included studies to demonstrate the breadth of outcomes, stratified by country. We will then estimate mean (95% confidence interval) and median (IQR) outcomes for each country and, to the extent that data allow, each population group or other stratification, such as setting. Pooled analysis of studies will only be conducted if ?3 studies are identified that report outcomes for the same populations (e.g., "adults initiating therapy at rural primary health clinics in Zambia").

Analysis of subgroups or subsets

If data allow, we plan to stratify our results by population (age, sex, condition, other characteristics) and facility characteristics (setting, level, size). We will not be able to specify all potential sub-group analyses until we determine which sub-groups are included in the publications reviewed.

Contact details for further information

Sydney Rosen

sbrosen@bu.edu

For peer review only

Organisational affiliation of the review

Boston University School of Public Health
https://www.bu.edu/sph/about/departments/global-health/

Review team members and their organisational affiliations

Dr Mariet Benade. Department of Global Health, Boston University School of Public Health, Boston, Massachusetts, USA
Dr Mhairi Maskew. Health Economics and Epidemiology Research Office, Faculty of Health Sciences, University of the Witwatersrand, Johannesburg, South Africa
Ms Linda Sande. Health Economics and Epidemiology Research Office, Faculty of Health Sciences, University of the Witwatersrand, Johannesburg, South Africa
Professor Nancy Scott. Department of Global Health, Boston University School of Public Health, Boston, Massachusetts, USA
Ms Allison Juntunen. Department of Global Health, Boston University School of Public Health, Boston, Massachusetts, USA
Professor Sydney Rosen. Department of Global Health, Boston University School of Public Health, Boston, Massachusetts, USA

Type and method of review

Epidemiologic, Systematic review

Anticipated or actual start date

10 April 2022

Anticipated completion date

10 July 2022

Funding sources/sponsors

Research reported in this publication was supported by the Bill & Melinda Gates Foundation Investment Number INV-031690, Retain6: Models of care for the first six months of HIV treatment.

Grant number(s)

State the funder, grant or award number and the date of award

Bill & Melinda Gates Foundation Investment Number INV-031690. October 2021.

Conflicts of interest

Language

English

Country

Malawi, South Africa, United States of America, Zambia

Stage of review

Review Ongoing

Subject index terms status

Subject indexing assigned by CRD

Subject index terms

MeSH headings have not been applied to this record

Date of registration in PROSPERO

08 May 2022

Date of first submission

For peer review only

07 April 2022

Stage of review at time of this submission

Stage	Started	Completed
Preliminary searches	Yes	No
Piloting of the study selection process	No	No
Formal screening of search results against eligibility criteria	No	No
Data extraction	No	No
Risk of bias (quality) assessment	No	No
Data analysis	No	No

The record owner confirms that the information they have supplied for this submission is accurate and complete and they understand that deliberate provision of inaccurate information or omission of data may be construed as scientific misconduct.
The record owner confirms that they will update the status of the review when it is completed and will add publication details in due course.

Versions

08 May 2022
08 May 2022

For peer review only

File S2. PRISMA checklists for manuscripts and abstracts

Topic	No.	Item	Location where item is reported
**TITLE**			
**Title**	1	Identify the report as a systematic review.	Title
**ABSTRACT**			
**Abstract**	2	See the PRISMA 2020 for Abstracts checklist	
**INTRODUCTION**			
**Rationale**	3	Describe the rationale for the review in the context of existing knowledge.	Page 3
**Objectives**	4	Provide an explicit statement of the objective(s) or question(s) the review addresses.	Page 3
**METHODS**			
**Eligibility criteria**	5	Specify the inclusion and exclusion criteria for the review and how studies were grouped for the syntheses.	Page 4
**Information sources**	6	Specify all databases, registers, websites, organisations, reference lists and other sources searched or consulted to identify studies. Specify the date when each source was last searched or consulted.	Page 4
**Search strategy**	7	Present the full search strategies for all databases, registers and websites, including any filters and limits used.	Page 4, Supplementary
**Selection process**	8	Specify the methods used to decide whether a study met the inclusion criteria of the review, including how many reviewers screened each record and each report retrieved, whether they worked independently, and if applicable, details of automation tools used in the process.	Page 4-5
**Data collection process**	9	Specify the methods used to collect data from reports, including how many reviewers collected data from each report, whether they worked independently, any processes for obtaining or confirming data from study investigators, and if applicable, details of automation tools used in the process.	Page 5
**Data items**	10a	List and define all outcomes for which data were sought. Specify whether all results that were compatible with each outcome domain in each study were sought (e.g. for all measures, time points, analyses), and if not, the methods used to decide which results to collect.	Page 5
	10b	List and define all other variables for which data were sought (e.g. participant and intervention characteristics, funding sources). Describe any assumptions made about any missing or unclear information.	Page 5
**Study risk of bias assessment**	11	Specify the methods used to assess risk of bias in the included studies, including details of the tool(s) used, how many reviewers assessed each study and whether they worked independently, and if applicable, details of automation tools used in the process.	Page 5

For peer review only

For peer review only - http://bmjopen.bmj.com/site/about/guidelines.xhtml

Topic	No.	Item	Location where item is reported
**Effect measures**	12	Specify for each outcome the effect measure(s) (e.g. risk ratio, mean difference) used in the synthesis or presentation of results.	Page 6
**Synthesis methods**	13a	Describe the processes used to decide which studies were eligible for each synthesis (e.g. tabulating the study intervention characteristics and comparing against the planned groups for each synthesis (item 5)).	N/A
	13b	Describe any methods required to prepare the data for presentation or synthesis, such as handling of missing summary statistics, or data conversions.	N/A
	13c	Describe any methods used to tabulate or visually display results of individual studies and syntheses.	Page 6
	13d	Describe any methods used to synthesize results and provide a rationale for the choice(s). If meta-analysis was performed, describe the model(s), method(s) to identify the presence and extent of statistical heterogeneity, and software package(s) used.	N/A
	13e	Describe any methods used to explore possible causes of heterogeneity among study results (e.g. subgroup analysis, meta-regression).	Page 5
	13f	Describe any sensitivity analyses conducted to assess robustness of the synthesized results.	N/A
**Reporting bias assessment**	14	Describe any methods used to assess risk of bias due to missing results in a synthesis (arising from reporting biases).	Page 5
**Certainty assessment**	15	Describe any methods used to assess certainty (or confidence) in the body of evidence for an outcome.	N/A
**RESULTS**			
**Study selection**	16a	Describe the results of the search and selection process, from the number of records identified in the search to the number of studies included in the review, ideally using a flow diagram.	Page 5
	16b	Cite studies that might appear to meet the inclusion criteria, but which were excluded, and explain why they were excluded.	Page 5
**Study characteristics**	17	Cite each included study and present its characteristics.	Page 6-7
**Risk of bias in studies**	18	Present assessments of risk of bias for each included study.	Page 8
**Results of individual studies**	19	For all outcomes, present, for each study: (a) summary statistics for each group (where appropriate) and (b) an effect estimate and its precision (e.g. confidence/credible interval), ideally using structured tables or plots.	Page 7
**Results of syntheses**	20a	For each synthesis, briefly summarise the characteristics and risk of bias among contributing studies.	N/A

For peer review only

Topic	No.	Item	Location where item is reported
	20b	Present results of all statistical syntheses conducted. If meta-analysis was done, present for each the summary estimate and its precision (e.g. confidence/credible interval) and measures of statistical heterogeneity. If comparing groups, describe the direction of the effect.	N/A
	20c	Present results of all investigations of possible causes of heterogeneity among study results.	Page 7
	20d	Present results of all sensitivity analyses conducted to assess the robustness of the synthesized results.	N/A
**Reporting biases**	21	Present assessments of risk of bias due to missing results (arising from reporting biases) for each synthesis assessed.	Page 8
**Certainty of evidence**	22	Present assessments of certainty (or confidence) in the body of evidence for each outcome assessed.	N/A
**DISCUSSION**			
**Discussion**	23a	Provide a general interpretation of the results in the context of other evidence.	Page 8
	23b	Discuss any limitations of the evidence included in the review.	Page 9
	23c	Discuss any limitations of the review processes used.	Page 9
	23d	Discuss implications of the results for practice, policy, and future research.	Page 9-10
**OTHER INFORMATION**			
**Registration and protocol**	24a	Provide registration information for the review, including register name and registration number, or state that the review was not registered.	Page 3
	24b	Indicate where the review protocol can be accessed, or state that a protocol was not prepared.	Page 3
	24c	Describe and explain any amendments to information provided at registration or in the protocol.	Page 4
**Support**	25	Describe sources of financial or non-financial support for the review, and the role of the funders or sponsors in the review.	Page 10
**Competing interests**	26	Declare any competing interests of review authors.	Page 10
**Availability of data, code and other materials**	27	Report which of the following are publicly available and where they can be found: template data collection forms; data extracted from included studies; data used for all analyses; analytic code; any other materials used in the review.	Page 10

For peer review only

ABSTRACT	No.	Item	Reported?
**TITLE**			
**Title**	1	Identify the report as a systematic review.	Yes
**BACKGROUND**			
**Objectives**	2	Provide an explicit statement of the main objective(s) or question(s) the review addresses.	Yes
**METHODS**			
**Eligibility criteria**	3	Specify the inclusion and exclusion criteria for the review.	Yes
**Information sources**	4	Specify the information sources (e.g. databases, registers) used to identify studies and the date when each was last searched.	Yes
**Risk of bias**	5	Specify the methods used to assess risk of bias in the included studies.	Yes
**Synthesis of results**	6	Specify the methods used to present and synthesize results.	Yes
**RESULTS**			
**Included studies**	7	Give the total number of included studies and participants and summarise relevant characteristics of studies.	Yes
**Synthesis of results**	8	Present results for main outcomes, preferably indicating the number of included studies and participants for each. If meta-analysis was done, report the summary estimate and confidence/credible interval. If comparing groups, indicate the direction of the effect (i.e. which group is favoured).	Yes
**DISCUSSION**			
**Limitations of evidence**	9	Provide a brief summary of the limitations of the evidence included in the review (e.g. study risk of bias, inconsistency and imprecision).	Yes
**Interpretation**	10	Provide a general interpretation of the results and important implications.	Yes
**OTHER**			
**Funding**	11	Specify the primary source of funding for the review.	Yes
**Registration**	12	Provide the register name and registration number.	Yes

For peer review only - http://bmjopen.bmj.com/site/about/guidelines.xhtml

For peer review only

Additional table S1. Inclusion/exclusion criteria

Parameter	Inclusion criteria	Exclusion criteria
Population	Ages 18+ years; confirmed HIV positive status; presenting for initiation of any regimen of lifelong antiretroviral treatment	Paediatric and adolescent populations aged <18 years; currently only receiving ART for HIV prevention (PEP or PrEP)
Geographic region	Sub-Saharan Africa	None
Intervention	None, observational descriptive outcome	None
Study design	Reports primary, patient-level data from retrospective or prospective cohorts collected under any study design (trial, observational) with or without a comparison group; systematic reviews, meta-analyses	Case series or reports, purely qualitative studies, treatment guidelines, mathematical models, editorials, commentaries, study or trial protocols
Required descriptive data	Describes all of patients, location, timing of ART initiation, facility type, service delivery models and services provided to the public sector through government-managed public health infrastructure or through NGO/private programs or facilities that serve the uninsured sector	Insufficient description of the characteristics needed to describe the study population and outcome
Comparator	Not required; single arm evaluations are eligible	None
Outcomes	Reports proportion of patients initiating ART that are ART naïve and proportions of patients previously experienced on ART for any duration after initiation.	Insufficient detail provided to estimate of outcome
Timing	A majority of data collected for ART initiation on or after January 1, 2016	A majority of data accrued before January 1, 2016

For peer review only

For peer review only - http://bmjopen.bmj.com/site/about/guidelines.xhtml

Additional table S2. Search strategy

**PubMed**	
Population	("HIV Infections"[Mesh] OR HIV Infection OR Infection)<br>**AND**
Intervention	(treatment OR "Anti-HIV Agents"[MESH] OR "Anti-Retroviral Agents"[MESH] OR "Antiretroviral Therapy, Highly Active"[MESH])<br>**AND**
Outcomes	(compliance OR "Treatment adherence" OR undisclosed OR retention)<br>**AND**
Context	("Africa South of the Sahara"[Mesh] OR Sub-Saharan Africa OR Subsaharan Africa OR Africa, Sub-Saharan))
**Web of Science and EMBASE**	
Population	("HIV Infections" OR HIV Infection)<br>**AND**
Intervention	(treatment OR "Anti-HIV Agents" OR "Anti-Retroviral Agents" OR "Antiretroviral Therapy, Highly Active")<br>**AND**
Outcomes	(compliance OR "Treatment adherence" OR undisclosed OR retention)<br>**AND**
Context	("Africa South of the Sahara" OR Sub-Saharan Africa OR Subsaharan Africa OR Africa, Sub-Saharan)

Note: Results limited to 1 January 2018 – 15 September 2022

For peer review only

Additional table S4. Reasons for exclusion after full text review

Reason and number excluded	Explanation
Wrong time period (n=89)	All data or majority of data were gathered prior to 2016.
Wrong publication type (n=12)	Article was a protocol, review article or qualitative report without any quantitative descriptors.
Wrong population (n=115)	Population for main analysis included participants younger than 18yrs without stratification.<br>Population for main analysis excluded those with ART experience or required a minimum follow-up period after treatment initiation.
Not sufficient data for analysis (n=106)	No exclusion criteria met, but data reported was not sufficient for us to determine naïve vs non-naïve status.

For peer review only

BMJ Open

Prior exposure to antiretroviral therapy among adult patients presenting for HIV treatment initiation or re-initiation in sub- Saharan Africa: a systematic review

Journal:	*BMJ Open*
Manuscript ID	bmjopen-2022-071283.R1
Article Type:	Original research
Date Submitted by the Author:	09-Aug-2023
Complete List of Authors:	Benade, Mariet; Boston University School of Public Health, Department of Global Health; University of the Witwatersrand Johannesburg Faculty of Health Sciences, Health Economics and Epidemiology Research Office<br>Maskew, Mhairi; University of the Witwatersrand Johannesburg Faculty of Health Sciences, Health Economics and Epidemiology Research Office<br>Juntunen, Allison; Boston University School of Public Health, Department of Global Health<br>Rosen, Sydney; University of the Witwatersrand Johannesburg Faculty of Health Sciences, Health Economics and Epidemiology Research Office; Boston University School of Public Health, Department of Global Health
**Primary Subject Heading**:	HIV/AIDS
Secondary Subject Heading:	Global health, Health services research, Public health
Keywords:	EPIDEMIOLOGY, HIV & AIDS < INFECTIOUS DISEASES, PUBLIC HEALTH, Health Services Accessibility

SCHOLARONE™
Manuscripts

I, the Submitting Author has the right to grant and does grant on behalf of all authors of the Work (as defined in the below author licence), an exclusive licence and/or a non-exclusive licence for contributions from authors who are: i) UK Crown employees; ii) where BMJ has agreed a CC-BY licence shall apply, and/or iii) in accordance with the terms applicable for US Federal Government officers or employees acting as part of their official duties; on a worldwide, perpetual, irrevocable, royalty-free basis to BMJ Publishing Group Ltd ("BMJ") its licensees and where the relevant Journal is co-owned by BMJ to the co-owners of the Journal, to publish the Work in this journal and any other BMJ products and to exploit all rights, as set out in our licence.

The Submitting Author accepts and understands that any supply made under these terms is made by BMJ to the Submitting Author unless you are acting as an employee on behalf of your employer or a postgraduate student of an affiliated institution which is paying any applicable article publishing charge ("APC") for Open Access articles. Where the Submitting Author wishes to make the Work available on an Open Access basis (and intends to pay the relevant APC), the terms of reuse of such Open Access shall be governed by a Creative Commons licence – details of these licences and which Creative Commons licence will apply to this Work are set out in our licence referred to above.

Other than as permitted in any relevant BMJ Author's Self Archiving Policies, I confirm this Work has not been accepted for publication elsewhere, is not being considered for publication elsewhere and does not duplicate material already published. I confirm all authors consent to publication of this Work and authorise the granting of this licence.

For peer review only

Prior exposure to antiretroviral therapy among adult patients presenting for HIV treatment initiation or re-initiation in sub- Saharan Africa: a systematic review

Mariet Benade[a,b], Mhairi Maskew[b], Allison Juntunen[a], Sydney Rosen[a,b§]

[a]Department of Global Health, Boston University School of Public Health, Boston, MA, USA
[b]Health Economics and Epidemiology Research Office, Department of Internal Medicine, School of Clinical Medicine, Faculty of Health Sciences, University of the Witwatersrand, Johannesburg, South Africa

[§]Author for correspondence: Sydney Rosen, 801 Massachusetts Ave Rm 390, Boston, MA 02118, USA, sbrosen@bu.edu

MB: mbenade@bu.edu
MM: mmaskew@heroza.org
AJ: juntunen@bu.edu
SR: sbrosen@bu.edu

Running head: Proportion of non-naïve ART initiators in Africa

Word count: 4711 (main), 381 (abstract)

For peer review only

Abstract

Objectives

As countries have scaled up access to antiretroviral therapy (ART) for HIV, attrition rates of up to 30% annually have created a large pool of individuals who initiate treatment with prior ART experience. Little is known about the proportion of non-naïve re-initiators within the population presenting for treatment initiation.

Design

Systematic review of published articles and abstracts reporting proportions of non-naïve adult patients initiating ART in sub-Saharan Africa.

Data sources

PubMed, Embase Elsevier, Web of Science Core Collection, International AIDS Society conferences, CROI conferences.

Eligibility criteria

Clinical trials and observational studies; reporting on adults in sub-Saharan Africa who initiated lifelong ART; published between 1 January 2018 and 11 July 2023 and with data collected after January 2016. Self-report, laboratory discernment of antiretroviral metabolites, or viral suppression at initiation or in the medical record were accepted as evidence of prior exposure.

Data extraction and synthesis

We captured study and sample characteristics, proportions with previous ART exposure, and the indicator of previous exposure reported. We report results of each eligible study, estimate the risk of bias, and identify gaps in the literature.

Results

Of 2,740 articles, eleven, describing twelve cohorts contained sufficient information for the review. Proportions of initiators with evidence of prior ART use ranged from 5% (self-report only) to 53% (presence of ART metabolites in hair or blood sample). The vast majority of screened studies did not report naïve/non-naïve status. Metrics used to determine and report non-naïve proportions were inconsistent and difficult to interpret.

Conclusions

The proportion of patients initiating HIV treatment who are truly ART-naïve is not well documented. It is likely that 20% to 50% of ART patients who present for ART are re-initiators. Standard reporting metrics and diligence in reporting are needed, as is research to understand the reluctance of patients to report prior ART exposure.

Study registration number: Prospero CRD42022324136

Keywords: Antiretroviral therapy, Treatment naïve, Africa, Systematic review, Initiation, Re-initiation

For peer review only

Strengths and limitations of this study

- Comprehensive review using multiple databases and conference abstract archives.
- Focused on recent data in the era since access to antiretroviral therapy for HIV became universal, increasing the potential for re-initiation of treatment.
- Utilized best practices to develop search string, with multiple reiterations based on initial findings.
- Recognized a wide range of reporting indicators for prior exposure to antiretrovirals medications.
- Limited by the lack of standardized reporting of prior ARV exposure .

For peer review only

For peer review only - http://bmjopen.bmj.com/site/about/guidelines.xhtml

Introduction

The successful scale-up of access to antiretroviral therapy (ART) for HIV treatment in sub-Saharan Africa has produced a growing population of patients who have interrupted or stopped treatment sometime since they started, either permanently or temporarily. While very recent numbers on attrition from ART programs are scarce, retention in care rates for the region were reported to average 78% at 12 months after treatment initiation in a review published in 2015, suggesting that for a cohort of patients initiating in any given year, nearly a quarter have been lost from care one year later[1]. Many of these lost patients, however, proceed to "re-initiate" treatment in the months or years after dropping out of treatment programs[2]. Two estimates posit the extent of treatment re-initiation. The first, from the U.S. President's Emergency Plan for AIDS Relief (PEPFAR) reported that more than 580,000 patients returned to care after a treatment interruption in just the quarter from July to September 2020 in the countries that PEPFAR supports[3]. The second was from the Western Cape Province of South Africa, where among the subset of patients whose CD4 counts were less than 50 cells/mm^3, the proportion with prior treatment experience rose from 14% to 57% between 2008 and 2017[4].

Outside of indirect estimates such as those mentioned above, little is known about the actual proportions of non-naïve patients among all those presenting for ART initiation. Accurate data are difficult to obtain, largely because most HIV medical record systems neither distinguish between naïve and re-initiators nor allow tracking from one healthcare facility to another or over long intervals of inactivity. In most countries, a patient who originally initiated ART at one facility and then dropped out of care can easily present as a new patient at a nearby facility and be assumed to be ART-naïve. Self-reported information about a patient's naïve or non-naïve status may be unreliable, because patients who are known to have stopped treatment may be reprimanded, provided poorer service by healthcare facility staff, or required to participate in multiple adherence

For peer review only

training sessions, creating an incentive to present oneself as a new patient regardless of prior experience[5].

As the number of re-initiators continues to increase, understanding the proportion and characteristics of ART initiators who are not treatment-naïve is an important step in improving overall HIV treatment outcomes. By definition, most treatment re-initiators previously faced barriers to retention in care that they were unable to overcome in time or sufficiently to sustain continuity of treatment. Common barriers to retention include logistical challenges such as transport costs, psychosocial deterrents such as stigma, and personal preferences[6,7], and these barriers may become more prohibitive for patients who have already withdrawn from care once. Achieving long-term retention in care targets may thus require that healthcare systems differentiate interventions and services for re-initiators from those offered to naïve initiators. Re-initiators also comprise an increasing proportion of patients presenting with advanced HIV disease[4], who require additional care beyond simple ART initiation.

As national treatment programs mature, the proportion of ART initiators who are non-naïve will continue to grow, making the few available earlier estimates obsolete. To help fill the gap in empirical evidence on current proportions of naïve and non-naïve ART initiators, we conducted a comprehensive, rapid review of recently published or presented (2018 and later) peer-reviewed reports in sub-Saharan Africa that directly or indirectly presented data on re-initiation rates.

Methods

Using the Preferred Reporting Items for Systematic Reviews and Meta-Analyses (PRISMA) 2020 guidelines[8], we conducted a systematic review of peer-reviewed publications and published conference abstracts that reported on prior exposure to antiretroviral therapy among adult patients presenting for HIV treatment in sub-Saharan Africa. The review was registered on the International

For peer review only

Prospective Register of Systematic Reviews (PROSPERO CRD42022324136) (Supplementary File S1).

Search strategy, study selection, and data extraction

For this review, our primary outcome was the proportion of adults presenting for ART initiation (initially or after interruption) in public sector HIV treatment programs in sub-Saharan Africa who are not ART naïve. To indicate prior ART use, we accepted self-reported questionnaire responses, rates of viral load suppression at ART initiation (suggesting previous ART use), medical record evidence (e.g. a prior viral load test), and biological measurements of the presence of antiretroviral metabolites in blood, hair, or urine specimens at or prior to treatment initiation. Inclusion and exclusion criteria for the review are shown in Supplementary Table S1.

To identify potential sources, we developed a search string with the assistance of a medical librarian. The search syntax included variations of the terms HIV, treatment, antiretroviral therapy, retention, and adherence, limited to sub-Saharan Africa. During the course of the review, we made three revisions to the original protocol as submitted to PROSPERO, in order to maximize the potential for finding relevant sources. Specifically, we 1) included studies focusing on pregnant women starting ART in PMTCT programs; 2) added “undisclosed” and “retention” as search terms, as shown in Supplementary Table S2; and 3) included studies that required a minimum duration of ART for enrollment, as explained below. Search strings can be found in Supplementary Table S2.

We searched the PubMed, Embase (via Elsevier), and Web of Science Core Collection databases and published abstracts for the International AIDS Conference, International AIDS Society (IAS) Conference on HIV Science, and Conference on Retroviruses and Opportunistic Infections (CROI) with a search string developed to identify English-language publications which reported on HIV

For peer review only

For peer review only - http://bmjopen.bmj.com/site/about/guidelines.xhtml

treatment initiation in sub-Saharan Africa from 1 January 2018 until 31 March 2022. We further limited our search to articles from which the majority of the data were generated in 2016 or later, as this was when universal treatment access became common in the region and previous use became more likely. To capture the era of universal treatment access and allow time for guideline adoption, we considered studies published after 1 January 2018. Follow-up searches conducted on 11 July 2023 extended the latest publication or conference presentation date of the review to and updated our search phrases to include country names of countries in sub-Saharan Africa, using a pre-built search strategy created by the Canadian Health Libraries Association [9,10]. Searching for abstracts using Google Advanced search and limiting results to each conference's domain did not yield results, as such we scanned the abstract booklets for abstracts that reported on populations that were initiating ART treatment and searched conference archives with the key words "initiation," "naïve," "re-initiate," "newly," and "experience."

We included cohort, cross-sectional, case-control, and interventional studies that reported primary data on initiation of ART in the adult (≥18 years old) population, including for PMTCT. We included indexed pre-prints but excluded unpublished reports. We also excluded commentaries, modeling studies, and other sources that did not report primary data. While we excluded systematic reviews and meta-analyses, we manually searched existing systematic reviews for additional, non-duplicate references to be included. Where more than one publication reported on the same patient cohort, we chose the one that was either most recent or provided the most relevant data.

Because we were interested specifically in reports of the proportion of patients presenting for ART initiation or re-initiation in routine care who were naïve and non-naïve, we excluded studies that stated that prior ART experience was an exclusion criterion for the study, with two exceptions. If a study included only participants who self-reported as naïve but were then found to have evidence of previous antiretroviral (ARV) exposure, we included that proportion as a result, accepting that it

review only

explicitly omits patients who excluded themselves because of prior exposure and thus almost certainly reflects an underestimate of the true population prevalence of previous exposure. We also included studies that only enrolled patients who had achieved a specified duration of follow up on ART—whether one month or several years—despite the fact that these studies would have missed patients who were lost from care prior to reaching that specified duration.

All peer reviewed references identified using the respective search strings from PubMed, Embase and Web of Science were imported into Rayyan QCRI, where deduplication occurred. An initial, independent, blinded review (reviewers were not aware of each other's decisions) of the titles and abstracts was conducted by three study team members (MB, AJ, SR) using Rayyan QCRI. A full text review was then conducted for all publications remaining after the initial review by two study team members (MB, AJ or MB, SR), with conflicts resolved through discussion. Reasons for excluding publications were recorded during the full text review. As a quality check, one author (SR) also checked a sample (10%) of the excluded sources against exclusion criteria. At each stage of the review process any conflicts between reviewers were assessed and resolved though consensus of three authors (MB, AJ, SR). The results of the search were documented in accordance with the PRISMA-S reporting checklist (Supplementary File S2).

We created a data extraction tool to capture study and sample characteristics, proportions with previous ART exposure, and the type of indicator of previous exposure reported (e.g. self-report, laboratory results).

Outcomes and analysis

Our outcome of interest was the proportion of ART initiates who were treatment naïve at ART initiation, defined as a patient presenting for initiation of ART who has never previously taken

For peer review only

antiretroviral therapy for treatment of HIV ("new initiator"), compared the proportion who were treatment-experienced at ART initiation, defined as a patient presenting for initiation of ART who had previously taken antiretroviral therapy for HIV treatment but had interrupted that therapy for a minimum of 3 months ("re-initiator"). We accepted each paper's source of information about participants' status: self-report, medical record review, viral suppression, or laboratory tests for ARV metabolites and report that source in our results.

To evaluate the data, we first report each paper's outcome, with descriptive information regarding the population and setting to which the results apply. As described in the Prospero protocol, we had intended to estimate pooled results for individual countries and populations and to stratify by patient and facility characteristics, but we ultimately identified too few eligible sources to allow for any pooled or stratified analysis.

Quality of the eligible studies was assessed using the Joanna Briggs Institute Critical Appraisal Checklists[11].

Patient and public involvement

None.

Results

Sources identified

The results of the systematic search are shown in Figure 1. A total of 2,740 non-duplicate abstracts of peer reviewed journal articles and 9 abstracts from the selected conferences were screened. Systematic reviews that were screened are listed in Supplementary File S3. After the initial title and abstract review, 2,361 articles and abstracts were excluded, leaving 379 documents for full-text

For peer review only

For peer review only - http://bmjopen.bmj.com/site/about/guidelines.xhtml

review. During the full-text review, an additional 368 were excluded. Reasons for exclusions are reported in Supplementary Table S3. The primary reason for exclusion was lack of information on the naïve or non-naïve status of participants.

Figure 1. PRISMA flow chart

Eleven peer-reviewed articles were retained in the final data set for the full review, including one that reported data from two countries and will be included in our analysis as two studies, creating a total of twelve sets of results. The studies are described in Table 1.

Table 1. Studies included in the review

Source (alphabetical by country)	Country and location	Study design and source of data	Study population	Dates of presentation for ART initiation	Sample size	Sex (% female)	Age (median, IQR)
Lebelonyane 2020[12]	Botswana (national sample)	Subset of baseline data for intervention arm of cluster randomized trial of HIV prevention	Adults found to be HIV-positive during community HIV prevention campaign and linked to HIV care	June 2016-March 2018	800	55%	33 (26-41)
Buju 2022[13]	Democratic Republic of Congo (Bunia)	Observational prospective cohort of patients receiving dolutegravir	Adults presenting for ART initiation or ongoing ART treatment at all ART clinics in Busia	July 2019-July 2021 for those initiating during the study; earlier for those already on ART	177	69%	39 (12)*
Genet 2021 [14]	Ethiopia (Northwest Ethiopia)	Observational, cross sectional study of first line treatment failure	Adults[†] who had received at least 6 months of ART	May October 2017	430	58%	38 (12-67)**
Kunzweiler 2018 [15]	Kenya (Kisumu)	Observational prospective cohort	Adult men who have sex with men (MSM)	August 2015-September 2016	63	0%	27 (22-32)
Rosen 2019[16]	Kenya (Kericho, Kapsabet, and Kombewa counties)	Baseline data for intervention arm of clinical trial of same-day ART initiation	Adults presenting for ART initiation at 3 public sector hospitals	July 2017-April 2018	477	58%	36 (29-44)
Barnabas 2022 [17]	South Africa (KwaZulu Natal Province)	Baseline data from clinical trial of fee for ART home delivery	Adults living in study community and presenting for ART initiation	October 2019 – January 2020	6	46%[#]	36 (31-43)[#]

For peer review only

Source (alphabetical by country)	Country and location	Study design and source of data	Study population	Dates of presentation for ART initiation	Sample size	Sex (% female)	Age (median, IQR)
Dorward 2020[18]	South Africa (KwaZulu Natal Province)	Baseline data for randomized controlled trial for point-of-care HIV viral load testing	Adults clinically stable on ART and due for 6-month viral load testing	August 2016-February 2017 (est)	390	60%	32 (27-38)
Maskew 2020[19]	South Africa (Gauteng Province)	Baseline data for intervention arm in clinical trial of same-day ART initiation	Adults presenting for ART initiation at 3 public sector clinics	March-September 2018	296	64%	35 (30-44)
Mavhandu-Ramarumo 2019[20]	South Africa (Limpopo Province)	Baseline samples from clinical trial of drug resistance	Adults presenting for ART initiation at 3 public sector clinics	2017-2019	77	90%	35 (27-42)
Rosen 2019 [16]	South Africa (Gauteng Province)	Baseline data for intervention arm of clinical trial of same-day ART initiation	Adults presenting for ART initiation at 3 public sector clinics	March-July 2017	600	63%	34 (29-41)
Sithole 2021[21]	South Africa (KwaZulu Natal Province)	Subset of baseline data from clinical trial of home-based ART initiation	Non-pregnant adults presenting for ART initiation at 2 public sector clinics, with CD4 >100 and no active TB	February 2018-November 2018	193	60%	Not reported
Pry 2023[22]	Zambia (Lusaka)	Subset from implementation trial that utilized viral load testing at baseline	Adults diagnosed and initiating ART at 2 government facilities	May 2021 – March 2022	248	63%	30 (25-37)

*Mean (standard deviation)
†Study included children under 18, but they comprised <7% of the study sample
**Mean (minimum-maximum)
#Study enrolled larger sample of patients already on ART—only 6 were reported as initiating or re-initiating. Characteristics reported are for entire study population, not solely for subsample of 6 who initiated treatment.

Six of the studies in Table 1 were conducted in South Africa and one per country in Botswana, the Democratic Republic of Congo (DRC), Ethiopia, Kenya, and Zambia. Nine of the twelve reported baseline data from a clinical trial conducted for other purposes, while three were observational studies. All were very or somewhat small in size, enrolled adult patients presenting or having previously presented for routine ART initiation at public sector clinics, and, with the exception of Pry 2023, collected most or all data prior to the disruption in service delivery caused by COVID-19 in

For peer review only

early 2020[23]. Specific populations enrolled varied by study, though only one, Kunzweiler 2018, was limited to a non-general adult population.

Proportions of patients non-naïve

In Table 2, we report the proportion of patients in each study reported to be non-naïve when presenting for ART initiation.

Table 2. Proportions of cohorts reported to be non-naïve at ART initiation

Source	Source of data	Proportion non-naïve at initiation	Inclusion criteria related to prior ART exposure	Comments
Lebelonyane 2020*	Not stated; only indicated as participants who had “previous ART treatment”	54/800 (7%)	Sample included 16-17 year olds; minors may be less likely than adults to have had an opportunity for prior ART exposure**.	Participants were identified through community-based testing and referred for ART; sample does not represent routine walk-in ART initiation population.
Buju 2022	Viral load suppressed at ART initiation	93/177 (52%)	None.	Enrolled both initiators and patients already on ART; results presented are only for initiators.
Genet 2021	Self-report	90/430 (21%)	Sample included 12-17 year olds†; minors may be less likely than adults to have had an opportunity for prior ART exposure. Participants required to have completed 6 months on ART; patients lost to follow up before reaching 6 months were excluded.	Enrolled patients younger than 18. Those on second line ART were excluded; patients eligible for second line treatment may be more likely to be re-initiators.
Kunzweiler 2018	Viral load suppressed at ART initiation	19/63 (30%) (13 of 19 with suppressed viral load at ART initiation self-reported being ART-naïve)	Excluded 9 participants who were lost to follow up before reaching 6 months on ART.	Also excluded 3 patients who did not have viral load test results.
Rosen 2019	Self-report	18/240 (8%)	Included self-reported re-initiators who had interrupted ART for ≥ 3 months.	Patients who self-reported that they had previously initiated ART but had stopped for ≥3 months were eligible; those who self-reported that they had interrupted for < 3 months were excluded.
Barnabas 2022	Self-report and no existing record of ART at the study clinic	3/6 (50%)	Participants required to consent to a trial of payment for home ART; only clinically stable patients enrolled.	Enrolled both initiators and patients already on ART; results presented are only for initiators.

For peer review only - http://bmjopen.bmj.com/site/about/guidelines.xhtml

Source	Source of data	Proportion non-naïve at initiation	Inclusion criteria related to prior ART exposure	Comments
Dorward 2020	Self-report	18/390 (5%)	Participants required to have completed 6 months on ART; patients lost to follow up before reaching 6 months were excluded.	
Maskew 2020	Self-report	33/296 (11%)	Included self-reported re-initiators who had interrupted ART for ≥ 3 months	Patients who self-reported that they had previously initiated ART but had stopped for ≥3 months were eligible; those who self-reported that they had interrupted for < 3 months were excluded.
Mavhandu-Ramarumo 2019	Laboratory assay of blood and/or hair sample for presence of TDF, EFV, and/or FTC metabolites	41/77 (53%)	All participants self-reported as ART naïve	Stated enrollment criteria included naivete; those who had been on ART previously may have self-screened out of this study.
Rosen 2019	Self-report	7/298 (2%)	Included self-reported re-initiators who had interrupted ART for ≥ 3 months	Patients who self-reported that they had previously initiated ART but had stopped for ≥3 months were eligible; those who self-reported that they had interrupted for < 3 months were excluded.
Sithole 2021††	Undetectable viral load at ART initiation	62/193 (32%) (Other outcomes reported: 42/193 (22%) had medical record evidence of prior ART use; 37/193 (19%) had detectable ARV metabolites in blood.)	All participants self-reported as ART naïve	Stated enrollment criteria included naivete; those who had been on ART previously may have self-screened out of this study. Participants were identified through community-based testing; sample does not represent routine walk-in ART initiation population. Primary DO-ART trial excluded 588 participants out of 2,479 who were virally suppressed at time of initiation and 66 who were found to already be on ART[24]
Pry 2023	Undetectable viral load at ART initiation	66/248 (27%) (Other outcomes reported: among 57 participants with suppressed VL who completed survey on prior ART use, 14% reported previous ART treatment)	None	Survey on silent transfers (self-reported prior ART use) was conducted at a follow-up visit after it was determined that participants had a suppressed viral load at initiation.

TDF, Tenofovir; EFV, Efavirenz; FTC, Emtricitabine
*A separate publication by the same author team, reporting data that overlapped with those reported in this study, stated that “22% of advanced HIV patients had previously been on ART.” [25]
**16-17 year olds receive treatment as adults in this setting.
†Minors under age 18 comprised <7% of the study sample.
††The parent study on which this study was based.

The proportion of patients presenting for ART initiation who were reported to be non-naïve ranged from 2 to 53%. It is important to note that the results shown in Table 2 are not strictly comparable to

For peer review only

For peer review only - http://bmjopen.bmj.com/site/about/guidelines.xhtml

286 one another, however, due to the different data sources, populations, and relevant exclusions listed in the right-hand column of the table. In Maskew 2021 and Rosen 2020, non-naïve patients were allowed to enroll as long as they had been off ART for at least three months. Nevertheless, these studies, which are the only ones that relied entirely on participant self-report, reported the lowest proportion of non-naïve participants. Barnabas 2023, which used both self-report and the facility's medical records, reported a much higher proportion with prior ART experience (50%) in the study's very small sample of initiators; rates varied between 33% and 66% previous exposure in the control and intervention arms, but that difference comprised only one individual in absolute terms. Two other studies in Table 2, Mavhandu-Ramarumo 2019 and Sithole 2021, explicitly excluded self-reported non-naïve patients; all patients enrolled in these studies claimed not to have been on ART previously. The relatively high proportions of non-naïve patients in these studies are thus still minimum estimates of the true proportion of ART initiators with prior treatment exposure at the study clinics, as anyone who admitted to prior use of ARVs will have been excluded from study enrolment. Similarly, Kunzweiler 2018 and Dorward 2020 excluded patients lost to follow up before 6 months after ART initiation, and thus may have underestimated the proportion re-initiating if non-naïve patients are more likely than naïve patients to drop out of care. Study participants were also drawn from diverse populations of individuals with HIV, including those identified in a community campaign (Lebelonyane 2020), in a household survey as part of a randomized trial (Sithole 2021), or as routine, walk-in presenters at clinics (Buju 2022, Maskew 2020, Rosen 2019).

Because all the eligible studies were small and/or had primary outcomes other than the proportion naive, little stratification of results by facility or patient characteristics was included. Mavhandu-Ramarumo 2019 reported that 7 of the 8 (88%) males in the sample population had evidence of prior ART exposure, compared to only 49% of the 34 females. In contrast, Sithole 2021 found that women (37%) were more likely to have evidence of undisclosed ART use than men (25%). Other characteristics that were associated with undisclosed ART use by undetectable viral load were

For peer review only - http://bmjopen.bmj.com/site/about/guidelines.xhtml

younger age (35% in 18-29 year olds, 30% in 30-49 year olds, and 23% among those >50 years) and living with a partner who was HIV positive (44% compared to 37%; adj OR 1.94 (95% CI 0.95-3.96)). Kunzweiler 2018's results apply specifically to MSM. Pry 2023 found that women ≥40 years had the highest probability of being non-naïve (42% 95%CI: 39.3-44.3%), while men aged 18-24 years had a baseline probability of just 12% (95% CI: 4.6-19.0%). Age was associated with higher rates of viral suppression, even when adjusting for sex, marital status, education, or facility of initiation. Being married and being female were both also associated with significantly increased adjusted prevalence ratios. Other studies did not provide information for stratification by facility or patient characteristics.

Quality of evidence

Each of the studies included in the review either presented baseline enrollment data from a randomized controlled trial or observational data. Outcomes after ART initiation were not relevant for our review, which looked only at the status of patients at baseline (ART initiation). For Maskew 2020 and Rosen 2019, data on self-reported naïve status were limited to the intervention arm. The setting, inclusion criteria, and baseline characteristics for these studies were clearly defined. Mavhandu-Ramarumo 2019, Lebelonyane 2020, Dorward 2020, Barnabas 2022, and Sithole 2021 also sufficiently defined inclusion criteria, study setting, and baseline characteristics.

We assessed Kunzweiler 2018, Genet 2021, Buju 2022, and Pry 2023 as observational studies. Kunzweiler 2018 utilised snowball sampling, as is frequently done among key populations at high risk of stigma. All four papers described the study setting and sample population of interest clearly, including age, sex and clinical characteristics of HIV presentation.

For peer review only

For peer review only - http://bmjopen.bmj.com/site/about/guidelines.xhtml

As is indicated in Table 2 and discussed further below, each of the studies included in this review used a different indicator of prior ART exposure (non-naivete), and most had limitations as to their accuracy and/or the representativeness of their populations.

Discussion

We systematically reviewed peer-reviewed evidence on the proportions of patients presenting for treatment initiation in sub-Saharan Africa who are or are not ART-naïve. The proportions non-naïve in the sources we found ranged from a low of 2% using self-report only to a high of 53% based on a laboratory analysis of ARV metabolites in blood and hair samples of patients who self-reported to be naïve.

Perhaps the most striking finding of this review is the sheer lack of published evidence to answer our research question. Despite a comprehensive search of the literature published between 2018 and 2022, and including data since 2016, we identified only eleven sources and twelve cohorts that reported this information, and most included it in only in passing. Half the studies were conducted in South Africa and were relatively small in size; most of those from other countries provided very little detail. Based on the published and presented research alone, it is fair to say that very little is known about the true proportion of ART initiators who are not treatment-naïve in South Africa, and almost nothing is known about the rest of the region or about specific sub-populations or risk groups. While it is possible that more information is available to program managers who have access to routinely collected medical record data, nothing in the literature suggests that such information is being generated on a large scale or, more important, utilized for program improvement.

During our search, we made a concerted effort to find additional eligible sources, in the hope that there would be more data to review and analyze. This included adding additional search terms,

For peer review only

For peer review only - http://bmjopen.bmj.com/site/about/guidelines.xhtml

including data on pregnant and post-partum women, and reviewing reference lists from relevant systematic reviews. We reviewed an unusually large number of full-text manuscripts in the hope that they would include proportions naïve and non-naïve in their cohort descriptions (typically "Table 1") even though there was no indication of this in the abstract.

Unfortunately, most of the sources that originally appeared promising were found to be ineligible, for various reasons. Most simply did not report on baseline naïve/non-naïve status at ART initiation, using any indicator. Some intentionally excluded non-naïve patients prior to enrolment and then reported all participants as naïve without further investigation. For studies that explicitly screened out non-naïve participants, we considered calculating proportions non-naive based on reported numbers of potential participants included and excluded, but we realized that many studies only applied the non-naïve criterion after screening potential participants out for other reasons. We thus could not safely rely on numbers screened out due to non-naïve status for our numerator and had to exclude those papers. Even some of the papers we did include either provided only a passing reference about prior ART exposure, making us uncertain that we interpreted them correctly (e.g. Lebelonyane 2020), or explicitly excluded patients lost to follow up in the early treatment period (e.g. Kunzweiler 2018 and Dorward 2020). Finally, several studies were excluded because we could not interpret the data reported or had doubts about the accuracy of prior exposure data based on the data sources used, even beyond the limitations discussed below.

For peer review only

The small number of eligible papers we did find offer some useful information. They used several different indicators for identifying non-naïve patients, and the indicators produced results that are consistent with their expected accuracy. Maskew 2020 and Rosen 2019, which relied solely on self-report, and Lebelonyane 2020, for with the source of data is unknown, found the lowest proportions non-naïve. Sithole 2021, which excluded a priori anyone self-reporting prior utilization, reported that 32% of patients had evidence of prior use based on being virally suppressed. Buju 2022 and

For peer review only - http://bmjopen.bmj.com/site/about/guidelines.xhtml

Kunzweiler 2018 reported that 52% and 30%, respectively, of patients presenting for initiation already had suppressed viral loads, suggesting prior ARV use. Mavhandu-Ramarumo 2019, which also excluded a priori anyone admitting prior utilization, using the most rigorous methodology with both blood and hair samples, estimated 53% of patients had prior ART exposure. The large observed difference between males and females and the small number of males in this study, however, suggest caution in applying the results to male patients. The expectation of viral rebound within 4 weeks of treatment interruption[26] suggests that viral suppression underestimates the true proportion non-naïve, as suppression as an indicator only captures recent treatment interrupters. Similarly, metabolite tests capture a maximum of about 90 days' prior exposure; anyone who interrupted treatment more than three months prior to study enrollment would be missed in the count of non-naïve initiators [27].

Based on results of the studies that used laboratory tests, we assume that those that relied on self-report alone—e.g. Maskew 2020, Rosen 2019, and Dorward 2020—underestimated the true proportion of participants who were non-naïve at initiation. Barnabas 2023, which combined self-report and same-facility record review, is an outlier among the studies using self-report, but the very small sample size of initiators (n=6) suggests caution in drawing conclusions from it. While study inclusion criteria may have biased the samples in Sithole 2021 and Mavhandu-Ramarumo 2019, these, together with Buju 2022, Kunzweiler 2018, Genet 2021, and Pry 2023 suggest that it is reasonable (and conservative) to conclude that between 20% and 50% of ART patients—and likely at least 30%--who present for ART are re-initiators. This proportion can be expected to increase with each passing year, as the number of truly naïve HIV-positive individuals declines. If this is so, then re-initiators comprise an important sub-population whose needs are likely to differ from those of naïve initiators and to whom service delivery should be tailored. This is especially important as recent evidence shows that mortality is significantly higher among PLHIV who interrupt and then reinitiate treatment, especially if the interruption occurred within 6 months of ART initiation.[28]

For peer review only

As is evident from the discussion above, this review had several limitations. First, while we believe that our search of the peer-reviewed, published literature and abstracts was thorough, the lack of standard terminology for describing prior ART exposure hampered the creation of precise search strings, and it is possible that some sources were missed. Second, we found information from only six of sub-Saharan Africa's 46 countries, and six of our twelve observations were from a single country, South Africa. Since each country in sub-Saharan Africa has a different experience with attrition from ART and approach to re-initiation, results may not be generalizable. Third, as explained in the introduction, even such data as are available tend to be incomplete, due to the limitations of self-reporting and of existing medical record systems.

Fourth, the wide range of results identified may reflect study methodologies, but it may also indicate substantial geographic diversity in outcomes that we cannot address with the data available. We speculate that additional relevant data are collected by program managers, ministries of health, and others but are either not analyzed to answer our research question or simply not published and therefore not accessible. Fifth, the small number of eligible sources, small sample sizes, and heterogeneity of research methods made it impossible to aggregate the results or produce meaningful summary statistics, beyond the range discussed above. Sixth, recall bias may be present in the studies that asked participants after six months or more on ART to self-report their naïve/non-naïve status at the time they initiated treatment. Seventh, none of the papers included reported the duration of the interval between a participant's prior ART experience and re-initiation. Some apparent re-initiators may in fact reflect unrecorded ("silent") transfers from one facility to another, without a medication interruption in between. This phenomenon may help explain the high proportion of "initiating" patients who already have viral suppression in Buju (2022), for example.

For peer review only - http://bmjopen.bmj.com/site/about/guidelines.xhtml

Finally, the fact that nine of 12 cohorts included were from clinical trials that provided patient compensation may have biased enrollments, though we cannot know how this may have affected patient enrolment or self-report of prior ART usage and the subsequent direction of the bias.

In addition to the sheer dearth of information available to answer our question, the search reported here revealed three important research priorities. First, there is a need for a standard terminology to describe patients with prior ART exposure and prior ART initiation experience. Even the binary terms “naïve” and “non-naïve” can be unclear if patients have previously used antiretroviral medications for PrEP or prevention of vertical transmission. Terms such as “ART experienced” and “ART exposed” are often substituted for non-naïve, without specification of what they refer to. Prior ART use may be “disclosed” or “self-reported.” Similarly, the duration of treatment interruption that leads to “re-initiation” is rarely specified, and “re-initiation” and “re-engagement” are used interchangeably. We can assume that patient returning to care after an interruption of less than one month is not likely to be regarded as a re-initiator, and a patient returning after an interruption of more than one year will likely be defined as a re-initiator. But what of patients with interruptions of 6 or 8 months? A common terminology for describing the phenomenon addressed here would be of great assistance in understanding its magnitude.

Second, in view of the potentially very high proportion of re-initiators among “new” ART patients, it is critical that researchers begin to report proportions naïve and non-naïve as a standard variable when describing patient cohorts, even if data come solely from self-report. We cannot determine from the literature whether many studies do collect this information but omit it from their reports or if it has simply not been collected. We identified several papers that came close to indicating a proportion non-naïve but did not explain their findings clearly enough to include in this review, suggesting that many studies do indeed have access to the relevant information. The study mentioned in our introduction from South Africa’s Western Cape Province, for example, provided

For peer review only

detailed information about patients with advanced HIV disease and suggested that more than a third of all patients with very low CD4 cell counts had previously been on ART but were now off, but we could not calculate the overall proportion of non-naïve initiators from the data reported [4]. In earlier years, when the proportion of non-naïve patients was low because treatment programs were still rapidly expanding, the question of prior ART experience may not have been a priority. In view of the results of the few studies available, it is clearly a priority now, for several reasons. First, retention in care will remain a challenge if those who disengaged from care previously remain at higher risk of disengaging from care again[29], unless the obstacles to continuity in care have been identified and addressed. Second, if service delivery needs do differ between ART naïve and ART experienced clients, treatment outcomes may also be affected by the large number of non-naive ART clients accessing services. Third, to the extent that resistance to first-line antiretroviral medications remains a concern, non-naïve patients may face higher risks of poorer responses to these medications after re-initiation.

Finally, the phenomenon of large numbers of patients who decline to reveal prior ART use, even when asked directly, is concerning. For studies that intend to limit participation to naïve patients, stated exclusion of those who are non-naïve may encourage non-disclosure, to avoid being denied study enrolment on this basis. Even so, it appears likely that many patients opt to lie about prior exposure. Other research suggests that they have good reason for doing so, as clinics may refuse to re-initiate those who admit to prior default and/or may provide poorer service to them[30–32]. Creating a clinic and community atmosphere that promotes honesty about prior exposure should also be a priority.

In conclusion, while we recognize that simply knowing the proportion of non-naïve patients in a given population will not in itself improve the quality of service delivery, measuring the size of the problem is a critical step in creating momentum to development and implement interventions

For peer review only

For peer review only - http://bmjopen.bmj.com/site/about/guidelines.xhtml

targeted specifically at re-initiators. Since these are patients who have already demonstrated that they face obstacles to remaining in care, identifying and targeting them for appropriate services is a vital step in improving the outcomes of treatment programs.

Supplemental files

Supplementary file S1. Search protocol (Prospero)

Supplementary file S2. PRISMA checklists for publications and abstracts

Supplementary file S3. Systematic reviews screened for potential articles

Supplementary Table S1. Inclusion/exclusion criteria for publications and abstracts

Supplementary Table S2. Search strategy

Supplementary Table S3. Reasons for exclusion after full-text review

Ethics approval and consent to participate

Not applicable; this is a systematic review of published information and has no human participants.

Availability of data and materials

All data used in this paper are included in the manuscript and publicly available in the cited sources.

Competing interests

The authors declare that they have no competing interests.

Funding

Funding for the study was provided the Bill & Melinda Gates Foundation through INV-031690 to Boston University. The funder had no role in study design, data collection and analysis, decision to publish, or preparation of the manuscript.

Author contributions

MM, SR, and MB conceived of and designed the study. MB, SR, and AJ identified and reviewed sources and extracted data. MB and SR analyzed the data and drafted the manuscript. All authors reviewed and edited the manuscript.

Acknowledgements

None

For peer review only

References

1. Fox MP, Rosen S. Retention of adult patients on antiretroviral therapy in low- and middle-income countries: systematic review and meta-analysis 2008–2013. J Acquir Immune Defic Syndr 2015;69: 98. doi:10.1097/QAI.0000000000000553
2. Ehrenkranz PD, Rosen S, Boulle A, Eaton J, Ford N, Fox M, et al. The revolving door of HIV care : Revising the service delivery cascade to achieve the UNAIDS 95-95-95 goals. PLoS Med 2021;18: e1003651. doi:10.1371/journal.pmed.1003651
3. Godfrey K. Supporting reengagement: What's new in DSD for HIV treatment: from WHO recommendations to reality. IAS 2021, July 18-21, 2021. Available from: https://differentiatedservicedelivery.org/Portals/0/adam/Content/TlGQJAsI5UqL5SHACJkSPg/File/IAS2021_treatmentcontinuity_IAS%20inputs%2030June.pdf. Accessed 19 Oct 2022.
4. Osler M, Hilderbrand K, Goemaere E, Ford N, Smith M, Meintjes G, et al. The continuing burden of advanced HIV disease over 10 years of increasing antiretroviral therapy coverage in South Africa. Clin Infect Dis 2018;66: S118–S125. doi:10.1093/cid/cix1140
5. Bisnauth MA, Davies N, Monareng S, Buthelezi F, Struthers H, McIntyre J, et al. Why do patients interrupt and return to antiretroviral therapy? Retention in HIV care from the patient's perspective in Johannesburg, South Africa. PLoS One 2021;16: 1–15. doi:10.1371/journal.pone.0256540
6. Mody A, Sikombe K, Beres LK, Simbeza S, Mukamba N, Eshun-Wilson I, et al. Profiles of HIV care disruptions among adult patients lost to follow-up in Zambia. J Acq Immun Defic Syndr 2021; 86: 62–72. doi:10.1097/qai.0000000000002530
7. Sikazwe I, Eshun-Wilson I, Sikombe K, Beres LK, Somwe P, Mody A, et al. Patient-reported reasons for stopping care or switching clinics in Zambia: A multisite, regionally representative estimate using a multistage sampling-based approach in Zambia. Clin Infect Dis 2021;73: e2294–e2302. doi:10.1093/cid/ciaa1501
8. Page MJ, McKenzie JE, Bossuyt PM, Boutron I, Hoffmann TC, Mulrow CD, et al. The PRISMA 2020 statement: An updated guideline for reporting systematic reviews. BMJ 2021;372.
9. Afrique subsaharienne [Biblio3S] [Internet]. Collaborative Directory of Documentary Resources of Quebec Health and Social Services Libraries. 2023. Available from: https://extranet.santecom.qc.ca/wiki/!biblio3s/doku.php?id=concepts:afrique-subsaharienne. Accessed 7 August 2023.
10. ISSG Search Filters Resource [Internet]. 2023. Available from: https://sites.google.com/a/york.ac.uk/issg-search-filters-resource/home. Accessed 7 August 2023.
11. Joanna Briggs Institute. Critical Appraisal Tools. Available from : https://jbi.global/critical-appraisal-tools. Accessed 6 Sep 2022.
12. Lebelonyane R, Bachanas P, Block L, Ussery F, Abrams W, Roland M, et al. Rapid antiretroviral therapy initiation in the Botswana Combination Prevention Project: a quasi-experimental before and after study. Lancet HIV 2020;7: e545–e553. doi:10.1016/S2352-3018(20)30187-9
13. Buju RT, Akilimali PZ, Tran N-T, Kamangu EN, Mesia GK, Kayembe JMN, et al. Determinants of survival of HIV patients receiving dolutegravir: a prospective cohort study in conflict-affected Bunia, Democratic Republic of Congo. Int J Environ Res Public Health 2022;19. doi:10.3390/ijerph191610220
14. Genet A, Mekonnen Z, Yizengaw E, Mekonnen D. First line antiretroviral treatment failure and associated factors among people living with HIV in northwest Ethiopia. Afr Health Sci 2021;21: 263–272. Available: https://pubmed.ncbi.nlm.nih.gov/34394306/
15. Kunzweiler CP, Bailey RC, Mehta SD, Okall DO, Obondi E, Djomand G, et al. Factors associated with viral suppression among HIV-positive Kenyan gay and bisexual men who have sex with men. AIDS Care 2018;30: S76–S88. doi:10.1080/09540121.2018.1510109

For peer review only - http://bmjopen.bmj.com/site/about/guidelines.xhtml

16. Rosen S, Maskew M, Larson BA, Brennan AT, Tsikhutsu I, Fox MP, et al. Simplified clinical algorithm for identifying patients eligible for same-day HIV treatment initiation (SLATE): Results from an individually randomized trial in South Africa and Kenya. PLoS Med 2019;16: e1002912. doi:10.1371/journal.pmed.1002912
17. Barnabas RV, Szpiro AA, Ntinga X, Mugambi ML, van Rooyen H, Bruce A, et al. Fee for home delivery and monitoring of antiretroviral therapy for HIV infection compared with standard clinic-based services in South Africa: a randomised controlled trial. Lancet HIV 2022;9(12):e848–56.
18. Dorward J, PK D, Osman F, Sookrajh Y, Pillay M, Moodley P, et al. Short communication: early antiretroviral therapy is associated with better viral suppression and less HIV drug resistance after implementation of universal treatment in South Africa. AIDS Res Hum Retroviruses 2020;36: 297–299. Available: https://pubmed.ncbi.nlm.nih.gov/31663368/
19. Maskew M, Brennan AT, Fox MP, Vezi L, Venter WDF, Ehrenkranz P, et al. A clinical algorithm for same-day HIV treatment initiation in settings with high TB symptom prevalence in South Africa: The SLATE II individually randomized clinical trial. PLoS Med 17(8): e1003226. https://doi.org/10.1371/journal.pmed.1003226
20. Mavhandu-Ramarumo LG, Tambe LAM, Matume ND, Katerere D, Bessong PO. Undisclosed exposure to antiretrovirals prior to treatment initiation: An exploratory analysis. South Afr J HIV Med 2021;22: 1–10. doi:10.4102/sajhivmed.v22i1.1200
21. Sithole N, Gunda R, Koole O, Krows M, Schaafsma T, Moshabela M, et al. Undisclosed antiretroviral therapy use at primary health care clinics in rural KwaZulu Natal South Africa: A DO-ART trial sub-study. AIDS Behav 2021. doi:10.1007/s10461-021-03319-4
22. Pry JM, Mwila C, Kapesa H, Mulabe M, Frimpong C, Moono M, et al. Estimating potential silent transfer using baseline viral load measures among people presenting as new to HIV care in Lusaka, Zambia: A cross-sectional study BMJ Open. 2023;13(5):1–8.
23. Benade M, Long L, Rosen S, Tucker J. Reduction in initiations of HIV treatment in South Africa during the COVID pandemic. BMC Health Serv Res 2022;22: 428. doi:10.1186/s12913-022-07714-y
24. Barnabas R, AA S, H van R, Asiimwe S, Pillay D, NC W, et al. Community-based antiretroviral therapy versus standard clinic-based services for HIV in South Africa and Uganda (DO ART): a randomised trial. Lancet Glob Health 2020;8: e1305–e1315. doi:10.1016/S2214-109X(20)30313-2
25. Lebelonyane R, Mills LA, Mogorosi C, Ussery F, Marukutira T, Theu J, et al. Advanced HIV disease in the Botswana combination prevention project: prevalence, risk factors, and outcomes. AIDS 2020;34:2223–30.
26. Mdluli T, Li Y, Pinyakorn S, Reeves DB, Cardozo-Ojeda EF, Yates A, et al. Acute HIV-1 infection viremia associate with rebound upon treatment interruption. Med. 2022;3(9):622-635.e3.
27. Anderson PL, Liu AY, Castillo-Mancilla JR, Gardner EM, Seifert SM, Mchugh C, et al. Intracellular Tenofovir-Diphosphate and Emtricitabine-Triphosphate in Dried Blood Spots following Directly Observed Therapy. Antimicrob Agents Chemother 2018;62:1–13.
28. Moolla H, Davies M, Davies C, Euvrard J, Prozeskry H, Fox M, et al. The effect of unplanned care interruptions on the mortality of adults resuming antiretroviral therapy in South Africa: a survival analysis. 12th International AIDS Society Conference on HIV Science, Brisbane, abstract OAC0104, 2023.
29. Inzaule SC, Kroeze S, Kityo CM, Siwale M, Akanmu S, Wellington M, et al. Long-term HIV treatment outcomes and associated factors in sub-Saharan Africa: multicountry longitudinal cohort analysis. AIDS 2022; 36:1437–47.
30. Bisnauth MA, Davies N, Monareng S, Buthelezi F, Struthers H, McIntyre J, et al. Why do patients interrupt and return to antiretroviral therapy? Retention in HIV care from the patient's perspective in Johannesburg, South Africa. PLoS ONE 2021; 16:e0256540. doi:10.1371/journal.pone.0256540

For peer review only - http://bmjopen.bmj.com/site/about/guidelines.xhtml

31. Rees K, Bisnauth M, O'Connor C, Ramvhulela T, Vali N. Understanding patients reinitiating antiretroviral therapy in two South African districts. South Afr J HIV Med 2022;23. doi:10.4102/sajhivmed.v23i1.1380
32. Bisnauth MA, Davies N, Monareng S, Struthers H, McIntyre JA, Rees K. Exploring healthcare workers' experiences of managing patients returning to HIV care in Johannesburg, South Africa. Glob Health Action 2022;15. doi:10.1080/16549716.2021.2012019.

For peer review only

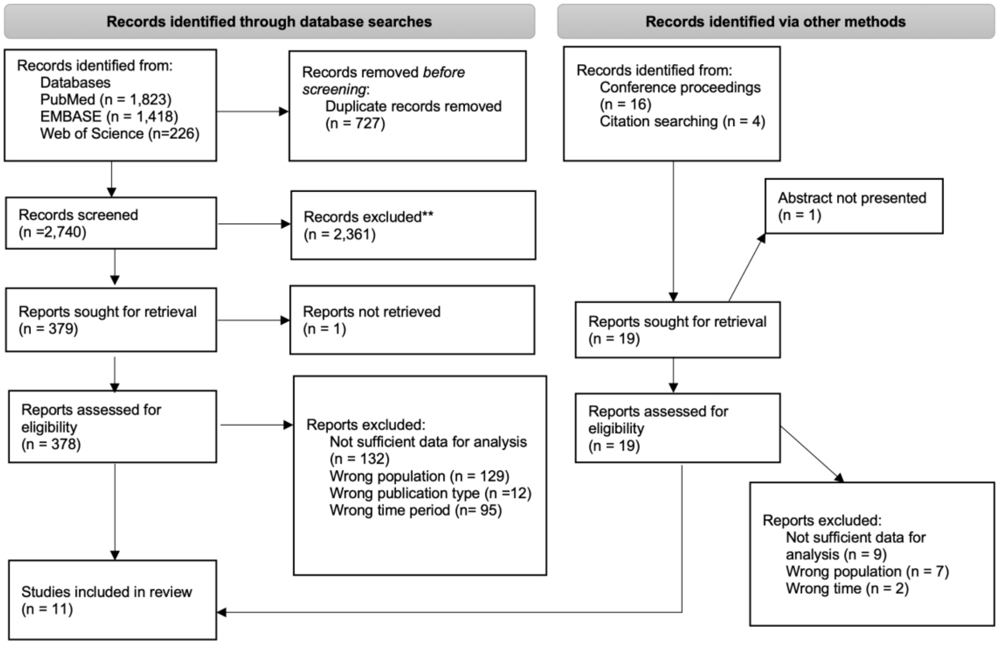

Prisma flow chart

893x573mm (72 x 72 DPI)

NIHR | National Institute for Health Research

PROSPERO
International prospective register of systematic reviews

To enable PROSPERO to focus on COVID-19 submissions, this registration record has undergone basic automated checks for eligibility and is published exactly as submitted. PROSPERO has never provided peer review, and usual checking by the PROSPERO team does not endorse content. Therefore, automatically published records should be treated as any other PROSPERO registration. Further detail is provided here.

Citation

Mariet Benade, Mhairi Maskew, Linda Sande, Nancy Scott, Allison Juntunen, Sydney Rosen. Prior exposure to antiretroviral therapy among adult patients presenting for HIV treatment initiation or re-initiation in sub-Saharan Africa: a systematic review. PROSPERO 2022 CRD42022324136 Available from: https://www.crd.york.ac.uk/prospero/display_record.php?ID=CRD42022324136

Review question

What proportions of adult patients presenting for ART initiation or re-initiation in public sector HIV treatment programs in Southern Africa are (a) ART naïve (new initiators) and (b) treatment experienced (re-initiators)?

Searches

We will search PubMed, Embase, and Web of Science and abstracts from the International AIDS Conference, International AIDS Society (IAS) Conference on HIV Science, and Conference on Retroviruses and Opportunistic Infections (CROI). We will manually search reference lists from sources identified in the search. Unpublished studies (e.g. preprints) will be included. Searches will be limited to English language publications.

The search period will be from January 1, 2018 to March 31, 2022. Inclusion in the review will be limited to sources for which a majority of individual-level data pertain to 2016 or later, as 2016 was the year when universal treatment access became common in sub-Saharan Africa. If sources report data from both before and after January 1, 2016, only results pertaining to the data from after that date will be used.

The search will be re-run prior to final analysis to identify very recent reports.

Types of study to be included

Studies that report initiation of ART for adult patients. These can be cohort, case control, cross-sectional, or interventional studies. White papers, commentaries, modeling studies, cost-effectiveness studies, and case studies will be excluded. Qualitative studies will be excluded unless there is specific data on treatment experience at ART initiation. Existing systematic reviews will be evaluated for additional, non-duplicate references. Published protocols with no data will be excluded, but if the study meets the inclusion criteria, we will attempt to find study results to evaluate for inclusion.

Condition or domain being studied

The successful scale-up of access to ART for HIV in sub-Saharan Africa has produced a growing population of patients who have interrupted or stopped treatment, and are now “re-initiating” therapy. While a diminishing number utilized ART temporarily for prevention of mother-to-child transmission prior to universal eligibility for ART, most are patients who stopped treatment, despite guidelines recommending lifelong use. Estimates of the proportion of patients presenting for ART re-initiation range from 10% to 50%.

Treatment re-initiators previously faced barriers to retention in care that they were unable to overcome. Achieving long-term retention in care targets may require that healthcare systems differentiate interventions and services for re-initiators from those offered to naïve initiators.

A first step in improving retention in care for ART re-initiators is to estimate how many individuals there are in any population of patients presenting for ART initiation. Few such estimates are available, largely because most healthcare data systems do not distinguish between naïve and re-initiators, allow tracking from one

For peer review only - http://bmjopen.bmj.com/site/about/guidelines.xhtml

healthcare facility to another or over long intervals of inactivity. To provide a baseline for considering this challenge, we'll search the recent literature for estimates of the proportion of patients who are re-initiators in sub-Saharan Africa.

Participants/population

Inclusion: Adults (?18 years of age) living with HIV presenting for initiation of any regimen of lifelong antiretroviral treatment in any sub-Saharan African country.
Exclusion: Individuals presenting for initiation of antiretroviral medications for any reason other than therapeutic (e.g. PrEP, PMTCT).

Intervention(s), exposure(s)

Initiation of ART is the intervention of interest. This will be an observational study of the characteristics of individuals initiating ART for HIV treatment (specifically, whether or not they have prior exposure to ART at the time of initiation).

Comparator(s)/control

Not applicable. Eligible studies need only report the proportions of patients who are treatment naïve and those who are treatment experienced.

Context

Initiation of ART in routine care settings in sub-Saharan Africa, including any type of facility or provider.

Main outcome(s)

Proportion of patients who are:
a. Treatment naïve at ART initiation, defined as a patient presenting for initiation of ART who has never previously taken antiretroviral therapy for treatment of HIV (new initiator); or
b. Treatment experienced at ART initiation, defined as a patient presenting for initiation of ART who has previously taken antiretroviral therapy for HIV treatment but has interrupted that therapy for a minimum of 3 months (re-initiator).

Measures of effect

Proportion, odds ratio

Additional outcome(s)

If data allow, we will also estimate the primary outcomes stratified by patient and/or facility characteristics, such as patient age and sex and facility country and setting.

Measures of effect

Proportion

Data extraction (selection and coding)

Study selection:

Rayyan will be used to support screening of studies and track inclusion or exclusion of studies. References will be managed in Mendeley, and duplicates will be removed. Two independent reviewers will be involved in each phase of review, including title and abstract screening, full-text screening, and data extraction. Title and abstract screening will include a pilot test to assess if the reviewers are able to achieve over 90% agreement on inclusion based on the eligibility criteria listed in this protocol. Disagreements will be resolved through discussion to achieve consensus. After full text screening, relevant non-duplicate citations from previous systematic reviews and other studies labelled for snowball in the initial search will be added for inclusion/exclusion consideration in a snowball phase.

For peer review only

Data extraction:

Data extraction will use a pre-determined template that captures all relevant variables. Data extraction will be done independently by two reviewers and discrepancies will be resolved by consensus. Data items to be extracted from studies include:

- Country
- District or locality
- Facility type
- Facility setting (urban, rural)
- Study year(s) or period(s) cohort observed
- Length of patient follow-up
- ART guidelines or protocol(s) in use during study period
- Study/cohort sample size
- Patient characteristics, including:
o Age distribution
o Gender distribution
o Clinical indicators of disease stage such as presenting CD4 count or WHO stage
o Other co-morbidities
- Study design (e.g., cohort, case control, cross-sectional, interventional)
- Descriptions of routine care, including whether the study was designed to influence retention
- Prior treatment exposure
o Proportion of adult patients ART treatment-naïve at initiation of ART
o Proportion of adult patients ART treatment-experienced at initiation of ART
o Basis for report of prior exposure (patient self-report, review of medical or laboratory records, tests of biological samples, pre-existing viral suppression, other)
- Authors' observations or comments on proportion of patients naïve or non-naïve, if any

Risk of bias (quality) assessment

Risk of bias and quality of individual studies that are included in this systematic review will be evaluated with the Joanna Briggs Institute critical appraisal checklist most relevant to the study type, as we expect that most included studies will be observational. The most important characteristics to be assessed will be recruitment biases (whether the study enrolled a representative sample of the specified population) and accuracy of naïve/non-naïve status reporting. Two reviewers will be involved in quality assessment, with disagreements resolved by a third reviewer on the study team.
We note that we expect that most studies meeting inclusion criteria for this review will report our outcomes (proportions naïve and non-naïve) as secondary outcomes or in their descriptions of their study cohorts, rather than as primary outcomes. (For example, a clinical trial with a primary outcome of viral suppression at 12 months after treatment initiation may report the proportion of participants who were naïve at initiation; our review would include the proportion naïve but not the primary outcome of viral suppression.)

Strategy for data synthesis

We will first present raw results from all included studies to demonstrate the breadth of outcomes, stratified by country. We will then estimate mean (95% confidence interval) and median (IQR) outcomes for each country and, to the extent that data allow, each population group or other stratification, such as setting. Pooled analysis of studies will only be conducted if ?3 studies are identified that report outcomes for the same populations (e.g., "adults initiating therapy at rural primary health clinics in Zambia").

Analysis of subgroups or subsets

If data allow, we plan to stratify our results by population (age, sex, condition, other characteristics) and facility characteristics (setting, level, size). We will not be able to specify all potential sub-group analyses until we determine which sub-groups are included in the publications reviewed.

Contact details for further information

Sydney Rosen

sbrosen@bu.edu

For peer review only

Organisational affiliation of the review

Boston University School of Public Health
https://www.bu.edu/sph/about/departments/global-health/

Review team members and their organisational affiliations

Dr Mariet Benade. Department of Global Health, Boston University School of Public Health, Boston, Massachusetts, USA
Dr Mhairi Maskew. Health Economics and Epidemiology Research Office, Faculty of Health Sciences, University of the Witwatersrand, Johannesburg, South Africa
Ms Linda Sande. Health Economics and Epidemiology Research Office, Faculty of Health Sciences, University of the Witwatersrand, Johannesburg, South Africa
Professor Nancy Scott. Department of Global Health, Boston University School of Public Health, Boston, Massachusetts, USA
Ms Allison Juntunen. Department of Global Health, Boston University School of Public Health, Boston, Massachusetts, USA
Professor Sydney Rosen. Department of Global Health, Boston University School of Public Health, Boston, Massachusetts, USA

Type and method of review

Epidemiologic, Systematic review

Anticipated or actual start date

10 April 2022

Anticipated completion date

10 July 2022

Funding sources/sponsors

Research reported in this publication was supported by the Bill & Melinda Gates Foundation Investment Number INV-031690, Retain6: Models of care for the first six months of HIV treatment.

Grant number(s)

State the funder, grant or award number and the date of award

Bill & Melinda Gates Foundation Investment Number INV-031690. October 2021.

Conflicts of interest

Language

English

Country

Malawi, South Africa, United States of America, Zambia

Stage of review

Review Ongoing

Subject index terms status

Subject indexing assigned by CRD

Subject index terms

MeSH headings have not been applied to this record

Date of registration in PROSPERO

08 May 2022

Date of first submission

For peer review only

07 April 2022

Stage of review at time of this submission

Stage	Started	Completed
Preliminary searches	Yes	No
Piloting of the study selection process	No	No
Formal screening of search results against eligibility criteria	No	No
Data extraction	No	No
Risk of bias (quality) assessment	No	No
Data analysis	No	No

The record owner confirms that the information they have supplied for this submission is accurate and complete and they understand that deliberate provision of inaccurate information or omission of data may be construed as scientific misconduct.
The record owner confirms that they will update the status of the review when it is completed and will add publication details in due course.

Versions

08 May 2022
08 May 2022

For peer review only

File S2. PRISMA checklists for manuscripts and abstracts

Topic	No.	Item	Location where item is reported
**TITLE**			
**Title**	1	Identify the report as a systematic review.	Title
**ABSTRACT**			
**Abstract**	2	See the PRISMA 2020 for Abstracts checklist	
**INTRODUCTION**			
**Rationale**	3	Describe the rationale for the review in the context of existing knowledge.	Page 5 (final paragraph)
**Objectives**	4	Provide an explicit statement of the objective(s) or question(s) the review addresses.	Page 5 (final paragraph)
**METHODS**			
**Eligibility criteria**	5	Specify the inclusion and exclusion criteria for the review and how studies were grouped for the syntheses.	Page 6 (Search strategy, study selection and data extraction)
**Information sources**	6	Specify all databases, registers, websites, organisations, reference lists and other sources searched or consulted to identify studies. Specify the date when each source was last searched or consulted.	Page 6 (Search strategy, study selection and data extraction)
**Search strategy**	7	Present the full search strategies for all databases, registers and websites, including any filters and limits used.	Page 4, Supplementary
**Selection process**	8	Specify the methods used to decide whether a study met the inclusion criteria of the review, including how many reviewers screened each record and each report retrieved, whether they worked independently, and if applicable, details of automation tools used in the process.	Page 7-8(Search strategy, study selection and data extraction)
**Data collection process**	9	Specify the methods used to collect data from reports, including how many reviewers collected data from each report, whether they worked independently, any processes for obtaining or confirming data from study investigators, and if applicable, details of automation tools used in the process.	Page 8 (Search strategy, study selection and data extraction)
**Data items**	10a	List and define all outcomes for which data were sought. Specify whether all results that were compatible with each outcome domain in each study were sought (e.g. for all measures, time points, analyses), and if not, the methods used to decide which results to collect.	Page 8-9 (Outcomes and analysis)
	10b	List and define all other variables for which data were sought (e.g. participant and intervention characteristics, funding sources). Describe any assumptions made about any missing or unclear information.	Page 8-9 (Outcomes and analysis

For peer review only - http://bmjopen.bmj.com/site/about/guidelines.xhtml

Topic	No.	Item	Location where item is reported
**Study risk of bias assessment**	11	Specify the methods used to assess risk of bias in the included studies, including details of the tool(s) used, how many reviewers assessed each study and whether they worked independently, and if applicable, details of automation tools used in the process.	Page 9 (Outcomes and analysis)
**Effect measures**	12	Specify for each outcome the effect measure(s) (e.g. risk ratio, mean difference) used in the synthesis or presentation of results.	Page 9 (Outcomes and analysis)
**Synthesis methods**	13a	Describe the processes used to decide which studies were eligible for each synthesis (e.g. tabulating the study intervention characteristics and comparing against the planned groups for each synthesis (item 5)).	N/A
	13b	Describe any methods required to prepare the data for presentation or synthesis, such as handling of missing summary statistics, or data conversions.	N/A
	13c	Describe any methods used to tabulate or visually display results of individual studies and syntheses.	Page 9 (Outcomes and analysis)
	13d	Describe any methods used to synthesize results and provide a rationale for the choice(s). If meta-analysis was performed, describe the model(s), method(s) to identify the presence and extent of statistical heterogeneity, and software package(s) used.	N/A
	13e	Describe any methods used to explore possible causes of heterogeneity among study results (e.g. subgroup analysis, meta-regression).	Page 9 (Outcomes and analysis)
	13f	Describe any sensitivity analyses conducted to assess robustness of the synthesized results.	N/A
**Reporting bias assessment**	14	Describe any methods used to assess risk of bias due to missing results in a synthesis (arising from reporting biases).	Page 9 (Outcomes and analysis)
**Certainty assessment**	15	Describe any methods used to assess certainty (or confidence) in the body of evidence for an outcome.	N/A
**RESULTS**			
**Study selection**	16a	Describe the results of the search and selection process, from the number of records identified in the search to the number of studies included in the review, ideally using a flow diagram.	Page 9-10 (Results: Sources identified)
	16b	Cite studies that might appear to meet the inclusion criteria, but which were excluded, and explain why they were excluded.	Page 17 (Discussion)
**Study characteristics**	17	Cite each included study and present its characteristics.	Page 11-12 (Sources identified, Table 1)
**Risk of bias in studies**	18	Present assessments of risk of bias for each included study.	Page 16 (Quality of evidence)

For peer review only - http://bmjopen.bmj.com/site/about/guidelines.xhtml
For peer review only

Topic	No.	Item	Location where item is reported
**Results of individual studies**	19	For all outcomes, present, for each study: (a) summary statistics for each group (where appropriate) and (b) an effect estimate and its precision (e.g. confidence/credible interval), ideally using structured tables or plots.	Page 13-14 (Table 2)
**Results of syntheses**	20a	For each synthesis, briefly summarise the characteristics and risk of bias among contributing studies.	N/A
	20b	Present results of all statistical syntheses conducted. If meta-analysis was done, present for each the summary estimate and its precision (e.g. confidence/credible interval) and measures of statistical heterogeneity. If comparing groups, describe the direction of the effect.	N/A
	20c	Present results of all investigations of possible causes of heterogeneity among study results.	Page 13 (Proportions of patients non-naïve)
	20d	Present results of all sensitivity analyses conducted to assess the robustness of the synthesized results.	N/A
**Reporting biases**	21	Present assessments of risk of bias due to missing results (arising from reporting biases) for each synthesis assessed.	Page 16 (Quality of evidence)
**Certainty of evidence**	22	Present assessments of certainty (or confidence) in the body of evidence for each outcome assessed.	N/A
**DISCUSSION**			
**Discussion**	23a	Provide a general interpretation of the results in the context of other evidence.	Page 16 (Discussion)
	23b	Discuss any limitations of the evidence included in the review.	Page 19
	23c	Discuss any limitations of the review processes used.	Page 19
	23d	Discuss implications of the results for practice, policy, and future research.	Page 20-22
**OTHER INFORMATION**			
**Registration and protocol**	24a	Provide registration information for the review, including register name and registration number, or state that the review was not registered.	Page 2 (Abstract)
	24b	Indicate where the review protocol can be accessed, or state that a protocol was not prepared.	Page 2 (Abstract)
	24c	Describe and explain any amendments to information provided at registration or in the protocol.	Page 6 (Methods, File S1)
**Support**	25	Describe sources of financial or non-financial support for the review, and the role of the funders or sponsors in the review.	Page 23 (Funding)
**Competing interests**	26	Declare any competing interests of review authors.	Page 23 (Competing interests)

For peer review only

Topic	No.	Item	Location where item is reported
**Availability of data, code and other materials**	27	Report which of the following are publicly available and where they can be found: template data collection forms; data extracted from included studies; data used for all analyses; analytic code; any other materials used in the review.	Page 23 (Availability of data and materials)

For peer review only

For peer review only - http://bmjopen.bmj.com/site/about/guidelines.xhtml

ABSTRACT	No.	Item	Reported?
**TITLE**			
**Title**	1	Identify the report as a systematic review.	Yes
**BACKGROUND**			
**Objectives**	2	Provide an explicit statement of the main objective(s) or question(s) the review addresses.	Yes
**METHODS**			
**Eligibility criteria**	3	Specify the inclusion and exclusion criteria for the review.	Yes
**Information sources**	4	Specify the information sources (e.g. databases, registers) used to identify studies and the date when each was last searched.	Yes
**Risk of bias**	5	Specify the methods used to assess risk of bias in the included studies.	Yes
**Synthesis of results**	6	Specify the methods used to present and synthesize results.	Yes
**RESULTS**			
**Included studies**	7	Give the total number of included studies and participants and summarise relevant characteristics of studies.	Yes
**Synthesis of results**	8	Present results for main outcomes, preferably indicating the number of included studies and participants for each. If meta-analysis was done, report the summary estimate and confidence/credible interval. If comparing groups, indicate the direction of the effect (i.e. which group is favoured).	Yes
**DISCUSSION**			
**Limitations of evidence**	9	Provide a brief summary of the limitations of the evidence included in the review (e.g. study risk of bias, inconsistency and imprecision).	Yes
**Interpretation**	10	Provide a general interpretation of the results and important implications.	Yes
**OTHER**			
**Funding**	11	Specify the primary source of funding for the review.	Yes
**Registration**	12	Provide the register name and registration number.	Yes

For peer review only

For peer review only - http://bmjopen.bmj.com/site/about/guidelines.xhtml

Supplemental file 3:
Systematic reviews screened for potential articles

1. Abdulrahman SA, Ganasegeran K, Rampal L, Martins OF. HIV Treatment Adherence - A Shared Burden for Patients, Health-Care Providers, and Other Stakeholders. AIDS Rev. 2019;21: 28–39. doi:10.24875/AIDSRev.19000037
2. Abebe Moges N, Olubukola A, Micheal O, Berhane Y. HIV patients retention and attrition in care and their determinants in Ethiopia: a systematic review and meta-analysis. BMC Infect Dis. 2020;20: 439. doi:10.1186/s12879-020-05168-3
3. Adugna Wubneh C, Dessalegn Mekonnen B, Wesenyeleh Delelegn M, Asmare Atalell K. Adherence to option B+ and its association with disclosure status and counseling among HIV-positive pregnant and lactating women in Ethiopia: systematic review and meta-analysis. Public Health. 2022;211: 105–113. doi:10.1016/J.PUHE.2022.07.016
4. Agutu CA, Ngetsa CJ, Price MA, Rinke de Wit TF, Omosa-Manyonyi G, Sanders EJ, et al. Systematic review of the performance and clinical utility of point of care HIV-1 RNA testing for diagnosis and care. PLoS One. 2019;14: e0218369. doi:10.1371/journal.pone.0218369
5. Ale BM, Amahowe F, Nganda MM, Danwang C, Wakaba NN, Almuwallad A, et al. Global burden of active smoking among people living with HIV on antiretroviral therapy: a systematic review and meta-analysis. Infect Dis Poverty. 2021;10: 12. doi:10.1186/s40249-021-00799-3
6. Belayneh Z, Mekuriaw B, Mehare T, Shumye S, Tsehay M. Magnitude and predictors of common mental disorder among people with HIV/AIDS in Ethiopia: a systematic review and meta-analysis. BMC Public Health. 2020;20: 689.
7. Brault MA, Spiegelman D, Abdool Karim SS, Vermund SH. Integrating and Interpreting Findings from the Latest Treatment as Prevention Trials. Curr HIV/AIDS Rep. 2020;17: 249–258. doi:10.1007/s11904-020-00492-4
8. Bulstra C, Hontelez J, Otto M, Stepanova A, Lamontagne E, Yakusik A, et al. Integrating HIV services and other health services: A systematic review and meta-analysis. Nosyk B, editor. PLoS Med. 2021;18: e1003836. doi:10.1371/journal.pmed.1003836
9. Casale M, Carlqvist A, Cluver L. Recent Interventions to Improve Retention in HIV Care and Adherence to Antiretroviral Treatment Among Adolescents and Youth: A Systematic Review. AIDS Patient Care STDS. 2019;33: 237–252.
10. Chammartin F, Zürcher K, Keiser O, Weigel R, Chu K, AN K, et al. Outcomes of Patients Lost to Follow-up in African Antiretroviral Therapy Programs: Individual Patient Data Meta-analysis. Clin Infect Dis an Off Publ Infect Dis Soc Am. 2018;67: 1643–1652. doi:10.1093/cid/ciy347
11. Chem ED, Van Hout MC, Hope V. Treatment outcomes and antiretroviral uptake in multidrug-resistant tuberculosis and HIV co-infected patients in Sub Saharan Africa: a systematic review and meta-analysis. BMC Infect Dis. 2019;19.
12. Chem E, Ferry A, Seeley J, HA W, Simms V. Health-related needs reported by adolescents living with HIV and receiving antiretroviral therapy in sub-Saharan Africa: a systematic literature review. J Int AIDS Soc. 2022;25: e25921. doi:10.1002/jia2.25921
13. Chimatira R, Ross A. A rapid review and synthesis of the effectiveness of programmes

For peer review only

initiating community-based antiretroviral therapy in sub-Saharan Africa. South Afr J HIV Med. 2020;21. doi:10.4102/SAJHIVMED.V21I1.1153
14. Cohn J, Ake J, Moorhouse M, Godfrey C. Sex Differences in the Treatment of HIV. Curr HIV/AIDS Rep. 2020;17: 373–384. doi:10.1007/s11904-020-00499-x
15. Damtie Y, Kefale B, Yalew M, Arefaynie M, Adane B, Muche A, et al. HIV risk behavior and associated factors among people living with HIV/AIDS in Ethiopia: A systematic review and meta-analysis. PLoS One. 2022;17: e0269304. doi:10.1371/journal.pone.0269304
16. Damulak PP, Ismail S, Abdul Manaf R, Mohd Said S, Agbaji O. Interventions to Improve Adherence to Antiretroviral Therapy (ART) in Sub-Saharan Africa: An Updated Systematic Review. Int J Env Res Public Heal. 2021;18. doi:10.3390/ijerph18052477
17. De Tomasi K, Mouala C. Good practices for retention in the circuit of prevention of Mother-Child Transmission of HIV in Sub-Saharan Africa: a systematic review of the literature. Med Sante Trop. 2019;29: 279–286. doi:10.1684/mst.2019.0911
18. Demena BA, Artavia-Mora L, Ouedraogo D, Thiombiano BA, Wagner N. A Systematic Review of Mobile Phone Interventions (SMS/IVR/Calls) to Improve Adherence and Retention to Antiretroviral Treatment in Low-and Middle-Income Countries. AIDS Patient Care STDS. 2020;34: 59–71. doi:10.1089/apc.2019.0181
19. Dessie G, Wagnew F, Mulugeta H, Amare D, Jara D, CT L, et al. The effect of disclosure on adherence to antiretroviral therapy among adults living with HIV in Ethiopia: a systematic review and meta-analysis. BMC Infect Dis. 2019;19: 528.
20. Diallo M, Adekpedjou R, Ahouada C, Ngangue P, Ly BA. Impact of pre-antiretroviral therapy cd4 counts on drug resistance and treatment failure: A systematic review. AIDS Rev. 2020;22: 78–92. doi:10.24875/AIDSRev.20000012
21. Dovel KL, Hariprasad S, Hubbard J, Cornell M, Phiri K, Choko A, et al. Strategies to improve antiretroviral therapy (ART) initiation and early engagement among men in sub-Saharan Africa: A scoping review of interventions in the era of universal treatment. Trop Med Int Heal. 2023;28: 454–465. doi:10.1111/tmi.13880
22. Duffy M, Madevu-Matson C, JE P, Zwick H, Sharer M, AM P. Systematic review: Development of a person-centered care framework within the context of HIV treatment settings in sub-Saharan Africa. Trop Med Int Health. 2022;27: 479–493. doi:10.1111/tmi.13746
23. Edessa D, Sisay M, Asefa F. Second-line HIV treatment failure in sub-Saharan Africa: A systematic review and meta-analysis. PLoS One. 2019;14: e0220159. doi:10.1371/journal.pone.0220159
24. Endalamaw A, Mekonnen M, Geremew D, Yehualashet FA, Tesera H, Habtewold TD. HIV/AIDS treatment failure and associated factors in Ethiopia: meta-analysis. BMC Public Health. 2020;20: 82. doi:10.1186/s12889-020-8160-8
25. Enslin D, Mallya P. Factors influencing treatment adherence in hypertension and HIV management in South Africa: A comparative literature review. South African Fam Pract. 2022;64. doi:10.4102/SAFP.V64I1.5434
26. Eshun-Wilson I, Awotiwon AA, Germann A, Amankwaa SA, Ford N, Schwartz S, et al. Effects of community-based antiretroviral therapy initiation models on HIV treatment outcomes: A systematic review and meta-analysis. PLoS Med. 2021;18: e1003646. doi:10.1371/journal.pmed.1003646

For peer review only

27. Finlay J, Lambert T, Krahn J, Meyer G, Singh AE, Caine V. Incentive-Based Human Immunodeficiency Virus Screening in Low- and Middle-Income Countries: A Systematic Review. Sex Transm Dis. 2022;49: 274–283. doi:10.1097/OLQ.0000000000001567
28. Fite RO. Association between adherence to Antiretroviral Therapy and place of residence among adult HIV infected patients in Ethiopia: A systematic review and meta-analysis. PLoS One. 2021;16: e0256948. doi:10.1371/journal.pone.0256948
29. Ford N, Migone C, Calmy A, Kerschberger B, Kanters S, Nsanzimana S, et al. Benefits and risks of rapid initiation of antiretroviral therapy. Aids. 2018;32: 17–23. doi:10.1097/qad.0000000000001671
30. Frijters EM, Hermans LE, Wensing AMJ, Devillé WLJM, Tempelman HA, De Wit JBF. Risk factors for loss to follow-up from antiretroviral therapy programmes in low-income and middle-income countries. AIDS. 2020;34: 1261–1288.
31. Fuseini H, Gyan BA, Kyei GB, Heimburger DC, Koethe JR. Undernutrition and HIV Infection in Sub-Saharan Africa: Health Outcomes and Therapeutic Interventions. Curr HIV/AIDS Rep. 2021;18: 87–97. doi:10.1007/s11904-021-00541-6
32. Geremew D, Melku M, Endalamaw A, Woldu B, Fasil A, Negash M, et al. Tuberculosis and its association with CD4(+) T cell count among adult HIV positive patients in Ethiopian settings: a systematic review and meta-analysis. BMC Infect Dis. 2020;20: 325.
33. Hagey JM, Li X, Barr-Walker J, Penner J, Kadima J, Oyaro P, et al. Differentiated HIV care in sub-Saharan Africa: a scoping review to inform antiretroviral therapy provision for stable HIV-infected individuals in Kenya. AIDS Care. 2018;30: 1477–1487. doi:10.1080/09540121.2018.1500995
34. Haghighat R, Steinert J, Cluver L. The effects of decentralising antiretroviral therapy care delivery on health outcomes for adolescents and young adults in low- and middle-income countries: a systematic review. Glob Health Action. 2019;12: 1668596. doi:10.1080/16549716.2019.1668596
35. Hargreaves J, Dalal S, Rice B, Anderegg N, Bhattacharjee P, Gafos M, et al. Repositioning Implementation Science in the HIV Response: Looking Ahead From AIDS 2018. J Acquir Immune Defic Syndr. 2019;82: S299–S304. doi:10.1097/QAI.0000000000002209
36. Hlongwa M, Mashamba-Thompson T, Makhunga S, Hlongwana K. Mapping evidence of intervention strategies to improving men's uptake to HIV testing services in sub-Saharan Africa: A systematic scoping review. BMC Infect Dis. 2019;19: 496. doi:10.1186/s12879-019-4124-y
37. Ibiloye O, Masquillier C, Jwanle P, S VB, J van O, Lynen L, et al. Community-Based ART Service Delivery for Key Populations in Sub-Saharan Africa: Scoping Review of Outcomes Along the Continuum of HIV Care. AIDS Behav. 2022;26: 2314–2337. doi:10.1007/s10461-021-03568-3
38. Jardim C, Zamani R, Akrami M. Evaluating the Impact of the COVID-19 Pandemic on Accessing HIV Services in South Africa: A Systematic Review. Int J Environ Res Public Health. 2022;19. doi:10.3390/ijerph191911899
39. Jopling R, Nyamayaro P, Andersen LS, Kagee A, Haberer JE, Abas MA. A Cascade of Interventions to Promote Adherence to Antiretroviral Therapy in African Countries. Curr HIV/AIDS Rep. 2020;17: 529–546. doi:10.1007/s11904-020-00511-4
40. Kadia BM, Dimala CA, Fongwen NT, Smith AD. Barriers to and enablers of uptake of

antiretroviral therapy in integrated HIV and tuberculosis treatment programmes in sub-Saharan Africa: a systematic review and meta-analysis. AIDS Res Ther. 2021;18. doi:10.1186/s12981-021-00395-3
41. Kebede HK, Mwanri L, Ward P, Gesesew HA. Predictors of lost to follow up from antiretroviral therapy among adults in sub-Saharan Africa: a systematic review and meta-analysis. Infect Dis POVERTY. 2021;10. doi:10.1186/s40249-021-00822.
42. Knight L, FC M, Schatz E. Behavioral and cognitive interventions to improve treatment adherence and access to HIV care among older adults in sub-Saharan Africa: an updated systematic review. Syst Rev. 2018;7: 114.
43. Kusemererwa S, Akena D, Nakanjako D, Kigozi J, Nanyunja R, Nanfuka M, et al. Strategies for retention of heterosexual men in HIV care in sub-Saharan Africa: A systematic review. PLoS One. 2021;16: e0246471. doi:10.1371/journal.pone.0246471
44. Laurenzi CA, du Toit S, Ameyan W, Melendez-Torres GJ, Kara T, Brand A, et al. Psychosocial interventions for improving engagement in care and health and behavioural outcomes for adolescents and young people living with HIV: a systematic review and meta-analysis. J Int AIDS Soc. 2021;24.
45. Le Tourneau N, Germann A, Thompson RR, Ford N, Schwartz S, Beres L, et al. Evaluation of HIV treatment outcomes with reduced frequency of clinical encounters and antiretroviral treatment refills: A systematic review and meta-analysis. PLoS Med. 2022;19: e1003959. doi:10.1371/journal.pmed.1003959
46. Limbada M, Zijlstra G, Macleod D, Ayles H, Fidler S. A systematic review of the effectiveness of non- health facility based care delivery of antiretroviral therapy for people living with HIV in sub-Saharan Africa measured by viral suppression, mortality and retention on ART. BMC Public Health. 2021;21: 1110. doi:10.1186/s12889-021-11053-8
47. Long L, Kuchukhidze S, Pascoe S, BE N, MP F, Cele R, et al. Retention in care and viral suppression in differentiated service delivery models for HIV treatment delivery in sub-Saharan Africa: a rapid systematic review. J Int AIDS Soc. 2020;23: e25640. doi:10.1002/jia2.25640
48. Makhado L, Mongale MP. Factors influencing non-adherence to antiretroviral therapy in South Africa: A systematic review. HIV AIDS Rev. 2019;18: 239–246.
49. Mateo-Urdiales A, Johnson S, Smith R, Nachega JB, Eshun-Wilson I. Rapid initiation of antiretroviral therapy for people living with HIV. Cochrane Database Syst Rev. 2019;2019. doi:10.1002/14651858.CD012962.pub2
50. Menon S, Benova L, Mabeya H. Epilepsy management in pregnant HIV plus women in sub-Saharan Africa, clinical aspects to consider: a scoping review. BMC Med. 2020;18.
51. Mugglin C. Gueler A., Vanobberghen F., Rice B., Egger M. KD. Apples and oranges: Assessment of the care cascade in sub-Saharan Africa. 22. doi:10.1002/jia2.25327
52. Muhula S, Gachohi J, Kombe Y, Karanja S. Interventions to improve early retention of patients in antiretroviral therapy programmes in sub-Saharan Africa: A systematic review. PLoS One. 2022;17: e0263663. doi:10.1371/journal.pone.0263663
53. Mukumbang FC, Orth Z, Van Wyk B. What do the implementation outcome variables tell us about the scaling-up of the antiretroviral treatment adherence clubs in South Africa? A document review. Heal Res Policy Syst. 2019;17.
54. Musumari PM, Techasrivichien T, Srithanaviboonchai K, Wanyenze RK, Matovu JKB,

For peer review only

Poudyal H, et al. HIV epidemic in fishing communities in Uganda: A scoping review. PLoS One. 2021;16.
55. Necho M, Belete A, Tsehay M. Depressive symptoms and their determinants in patients who are on antiretroviral therapy in the case of a low-income country, Ethiopia: a systematic review and meta-analysis. Int J Ment Health Syst. 2021;15. doi:10.1186/s13033-020-00430-2
56. Nyato D, Kuringe E, Drake M, Casalini C, Nnko S, Shao A, et al. Participants' accrual and delivery of HIV prevention interventions among men who have sex with men in sub-Saharan Africa: a systematic review. BMC Public Health. 2018;18: 370. doi:10.1186/s12889-018-5303-2
57. Nyoni T, Sallah YH, Okumu M, Byansi W, Lipsey K, Small E. The effectiveness of treatment supporter interventions in antiretroviral treatment adherence in sub-Saharan Africa: a systematic review and meta-Analysis. AIDS Care Psychol Socio-Med Asp AIDS HIV. 2020;32: 214–227.
58. Okonji EF, Mukumbang FC, Orth Z, Vickerman-Delport SA, Van Wyk B. Psychosocial support interventions for improved adherence and retention in ART care for young people living with HIV (10-24,Ääyears): a scoping review. BMC Public Health. 2020;20: 1841. doi:10.1186/s12889-020-09717-y
59. Omonaiye O, Kusljic S, Nicholson P, Manias E. Medication adherence in pregnant women with human immunodeficiency virus receiving antiretroviral therapy in sub-Saharan Africa: a systematic review. BMC Public Health. 2018;18: 805. doi:10.1186/s12889-018-5651-y
60. Omonaiye O, Nicholson P, Kusljic S, Manias E. A meta-analysis of effectiveness of interventions to improve adherence in pregnant women receiving antiretroviral therapy in sub-Saharan Africa. Int J Infect Dis IJID Off Publ Int Soc Infect Dis. 2018;74: 71–82.
61. Owusu K.K. Ahmed Z. A-GR. Strategies to improve linkage to hiv care in urban areas of Sub-Saharan Africa: A systematic review. 11. doi:10.2147/HIV.S216093
62. Passchier RV, Abas MA, Ebuenyi ID, Pariante CM. Effectiveness of depression interventions for people living with HIV in Sub-Saharan Africa: A systematic review & meta-analysis of psychological & immunological outcomes. Brain Behav Immun. 2018;73: 261–273. doi:10.1016/j.bbi.2018.05.010
63. Pellowski JA, Price DM, Harrison AD, Tuthill EL, Myer L, Operario D, et al. A Systematic Review and Meta-analysis of Antiretroviral Therapy (ART) Adherence Interventions for Women Living with HIV. AIDS Behav. 2019;23: 1998–2013. doi:10.1007/s10461-018-2341-9
64. Penn A, Azman H, Horvath H, KD T, MD H, Rajan J, et al. Supportive interventions to improve retention on ART in people with HIV in low- and middle-income countries: A systematic review. PloS one. 2018. p. e0208814. doi:10.1371/journal.pone.0208814
65. Rapaport SF, Peer AD, Viswasam N, Hahn E, Ryan S, Turpin G, et al. Implementing HIV Prevention in Sub-Saharan Africa: A Systematic Review of Interventions Targeting Systems, Communities, and Individuals. AIDS Behav. doi:10.1007/s10461-022-03751-0
66. Raphael M, Elizabeth S, Fiona V, Maja W. "Linkage to care" among people living with HIV in the era of "universal test and treat" in a sub-Sahara African setting. SWISS Med Wkly.

For peer review only

2021;151. doi:10.4414/smw.2021.20535
67. Roy M, Bolton Moore C, Sikazwe I, Holmes CB. A Review of Differentiated Service Delivery for HIV Treatment: Effectiveness, Mechanisms, Targeting, and Scale. Current HIV/AIDS Reports. Current Medicine Group LLC 1; 2019. pp. 324–334. doi:10.1007/s11904-019-00454-5
68. Sabapathy K, Hensen B, Varsaneux O, Floyd S, Fidler S, Hayes R. The cascade of care following community-based detection of HIV in sub-Saharan Africa – A systematic review with 90-90-90 targets in sight. PLoS One. 2018;13: e0200737. doi:10.1371/JOURNAL.PONE.0200737
69. Takarinda KC, Wallenta J, Scheve A, Mody A, Apollo T, Harries AD, et al. Measuring Retention in Antiretroviral Therapy Programs—a Synthetic Review of Different Approaches for Field Use in Low- and Middle-Income Settings. Curr Trop Med Reports. 2018;5: 179–185. doi:10.1007/S40475-018-0153-7
70. Too EK, Abubakar A, Nasambu C, Koot HM, Cuijpers P, Newton CRJC, et al. Prevalence and factors associated with common mental disorders in young people living with HIV in sub-Saharan Africa: a systematic review. J Int AIDS Soc. 2021;24.
71. Vrazo A, Firth J, Amzel A, Sedillo R, Ryan J, BR P. Interventions to significantly improve service uptake and retention of HIV-positive pregnant women and HIV-exposed infants along the prevention of mother-to-child transmission continuum of care: systematic review. Trop Med Int Health. 2018;23: 136–148. doi:10.1111/tmi.13014
72. Walsh F, Khan S, Bärnighausen T, Hettema A, Lejeune C, Mazibuko S, et al. Getting to 90-90-90: Experiences from the MaxART Early Access to ART for All (EAAA) Trial in Eswatini. Curr HIV/AIDS Rep. 2020;17: 324–332. doi:10.1007/s11904-020-00501-6
73. Yah CS, Tambo E. Why is mother to child transmission (MTCT) of HIV a continual threat to new-borns in sub-Saharan Africa (SSA). J Infect Public Health. 2019;12: 213–223.
74. Yendewa GA, Poveda E, Yendewa SA, Sahr F, Quiñones-Mateu ME, Salata RA. HIV/AIDS in Sierra Leone: Characterizing the Hidden Epidemic. AIDS Rev. 2018;20: 104–113. doi:10.24875/AIDSRev.M18000022
75. Yonga AM, Kiss L, Onarheim KH. A systematic review of the effects of intimate partner violence on HIV-positive pregnant women in sub-Saharan Africa. BMC Public Health. 2022;22: 220.
76. Zanoni B, Archary M, Sibaya T, Ramos T, Donenberg G, Shahmanesh M, et al. Interventions addressing the adolescent HIV continuum of care in South Africa: a systematic review and modified Delphi analysis. BMJ Open. 2022;12: e057797. doi:10.1136/bmjopen-2021-057797

For peer review only

Additional table S1. Inclusion/exclusion criteria

Parameter	Inclusion criteria	Exclusion criteria
Population	Ages 18+ years; confirmed HIV positive status; presenting for initiation of any regimen of lifelong antiretroviral treatment	Paediatric and adolescent populations; currently only receiving ART for HIV prevention (PEP or PrEP)
Geographic region	Sub-Saharan Africa	None
Intervention	None, observational descriptive outcome	None
Study design	Reports primary, patient-level data from retrospective or prospective cohorts collected under any study design (trial, observational) with or without a comparison group; systematic reviews, meta-analyses	Case series or reports, purely qualitative studies, treatment guidelines, mathematical models, editorials, commentaries, study or trial protocols
Required descriptive data	Describes all of patients, location, timing of ART initiation, facility type, service delivery models and services provided to the public sector through government-managed public health infrastructure or through NGO/private programs or facilities that serve the uninsured sector	Insufficient description of the characteristics needed to describe the study population and outcome
Comparator	Not required; single arm evaluations are eligible	None
Outcomes	Reports proportion of patients initiating ART that are ART naïve and proportions of patients previously experienced on ART for any duration after initiation.	Insufficient detail provided to estimate of outcome
Timing	A majority of data collected for ART initiation on or after January 1, 2016	A majority of data accrued before January 1, 2016

For peer review only

Additional table S2. Search strategy – conducted on 11 July 2023

**EMBASE**	
Population	('human immunodeficiency virus infection'/exp OR 'hiv infection')<br>**AND**
Intervention	('therapy'/exp OR ('anti human immunodeficiency virus agent'/exp OR 'antiretrovirus agent'/exp OR 'highly active antiretroviral therapy'/exp))<br>**AND**
Outcomes	('patient compliance'/exp OR 'treatment adherence' OR undisclosed OR 'retention'/exp)<br>**AND**
Context	(('africa south of the sahara'/exp OR 'africa south of the sahara' OR 'black africa' OR 'sub saharan africa' OR 'subsaharan africa') OR ('angola'/exp OR angola OR 'benin'/exp OR benin OR 'botswana'/exp OR botswana OR 'burkina faso'/exp OR 'burkina faso' OR 'burundi'/exp OR burundi OR 'cameroon'/exp OR cameroon OR 'cape verde'/exp OR 'cape verde' OR 'central african republic'/exp OR 'central african republic' OR 'chad'/exp OR chad OR 'comoros'/exp OR comoros OR 'congo'/exp OR congo OR brazzaville OR 'cote d ivoire' OR 'djibouti'/exp OR djibouti OR 'equatorial guinea'/exp OR 'equatorial guinea' OR 'eritrea'/exp OR eritrea OR 'ethiopia'/exp OR ethiopia OR 'gabon'/exp OR gabon OR 'gambia'/exp OR gambia OR 'ghana'/exp OR ghana OR 'guinea bissau'/exp OR 'guinea bissau' OR 'kenya'/exp OR kenya OR 'lesotho'/exp OR lesotho OR 'liberia'/exp OR liberia OR 'madagascar'/exp OR madagascar OR 'malawi'/exp OR malawi OR 'mali'/exp OR mali OR 'mauritania'/exp OR mauritania OR 'mauritius'/exp OR mauritius OR 'mozambique'/exp OR mozambique OR 'namibia'/exp OR namibia OR 'niger'/exp OR niger OR 'nigeria'/exp OR nigeria OR 'rwanda'/exp OR rwanda OR 'sao tome e principe' OR 'senegal'/exp OR senegal OR 'seychelles'/exp OR seychelles OR 'sierra leone'/exp OR 'sierra leone' OR 'somalia'/exp OR somalia OR 'south africa'/exp OR 'south africa' OR 'south sudan'/exp OR 'south sudan' OR 'sudan'/exp OR sudan OR 'swaziland'/exp OR swaziland OR 'tanzania'/exp OR tanzania OR 'togo'/exp OR togo OR 'uganda'/exp OR uganda OR 'western sahara'/exp OR 'western sahara' OR (western AND ('sahara'/exp OR sahara)) OR 'zaire'/exp OR zaire OR 'zambia'/exp OR zambia OR 'zimbabwe'/exp OR zimbabwe)) AND [2018-2023]/py
**PubMed**	
Population	("HIV Infections"[Mesh] OR HIV Infection)<br>**AND**
Intervention	((treatment OR "Anti-HIV Agents"[MESH] OR "Anti-Retroviral Agents"[MESH] OR "Antiretroviral Therapy, Highly Active"[MESH])<br>**AND**
Outcomes	(compliance OR "Treatment adherence" OR undisclosed OR retention)<br>**AND**
Context	("Africa South of the Sahara"[Mesh] OR Sub-Saharan Africa OR Subsaharan Africa OR Africa, Sub-Saharan OR Africa South of the Sahara OR Angola OR Benin OR Botswana OR "Burkina Faso" OR Burundi OR Cameroon OR "Cape Verde" OR "Central African Republic" OR Chad OR Comoros OR Congo OR Brazzaville OR "Cote d'Ivoire" OR Djibouti OR "Equatorial Guinea" OR Eritrea OR Ethiopia OR Gabon OR Gambia OR Ghana OR "Guinea Bissau" OR Kenya OR Lesotho OR Liberia OR Madagascar OR Malawi OR Mali OR Mauritania OR Mauritius OR Mozambique OR Namibia OR Niger OR Nigeria OR Rwanda OR "Sao Tome e Principe" OR Senegal OR Seychelles OR "Sierra Leone" OR Somalia OR "South

For peer review only - http://bmjopen.bmj.com/site/about/guidelines.xhtml

	Africa" OR "South Sudan" OR Sudan OR Swaziland OR Tanzania OR Togo OR Uganda OR Western Sahara OR Zaire OR Zambia OR Zimbabwe) AND (2018:2023[pdat])

**Web of Science Core Collection***	
Population	TS=("HIV Infections" OR HIV Infection)<br>**AND**
Intervention	TS=(treatment OR "Anti-HIV Agents" OR "Anti-Retroviral Agents" OR "Antiretroviral Therapy, Highly Active")<br>**AND**
Outcomes	TS= (compliance OR "Treatment adherence" OR undisclosed OR retention)<br>**AND**
Context	TS=("Africa South of the Sahara" OR Sub-Saharan Africa OR Subsaharan Africa OR Africa, Sub-Saharan OR Africa South of the Sahara OR Angola OR Benin OR Botswana OR "Burkina Faso" OR Burundi OR Cameroon OR "Cape Verde" OR "Central African Republic" OR Chad OR Comoros OR Congo OR Brazzaville OR "Cote d'Ivoire" OR Djibouti OR "Equatorial Guinea" OR Eritrea OR Ethiopia OR Gabon OR Gambia OR Ghana OR "Guinea Bissau" OR Kenya OR Lesotho OR Liberia OR Madagascar OR Malawi OR Mali OR Mauritania OR Mauritius OR Mozambique OR Namibia OR Niger OR Nigeria OR Rwanda OR "Sao Tome e Principe" OR Senegal OR Seychelles OR "Sierra Leone" OR Somalia OR "South Africa" OR "South Sudan" OR Sudan OR Swaziland OR Tanzania OR Togo OR Uganda OR Western Sahara OR Zaire OR Zambia OR Zimbabwe)

* Comprised of the Science Citation Index Expanded (1965 to the present), the Social Sciences Citation Index (1965 to the present), the Arts & Humanities Citation Index (1975 to the present), the Conference Proceedings Citation Index (both versions, Science and Social Sciences & the Humanities from 1990 to the present), the Book Citation Index (both versions, Science and Social Sciences & the Humanities from 2005 to the present) and the Emerging Sources Citation Index (2018 to the present)

Note: Results limited to 1 January 2018 – 11 July 2023

For peer review only - http://bmjopen.bmj.com/site/about/guidelines.xhtml

Additional table S3. Reasons for exclusion after full text review

Reason and number excluded	Explanation
Wrong time period (n=95)	All data or majority of data were gathered prior to 2016.
Wrong publication type (n=12)	Article was a protocol, review article or qualitative report without any quantitative descriptors.
Wrong population (n=129)	Population for main analysis included participants younger than 18yrs without stratification in a setting where adults are defined as older than 18yrs.<br>Population for main analysis excluded those with ART experience, unless other measures taken to confirm ART naivety. Population did not consists of participants presenting for ART initiation.
Not sufficient data for analysis (n=132)	No exclusion criteria met, but data reported was not sufficient for us to determine naïve vs non-naïve status.

For peer review only

For peer review only - http://bmjopen.bmj.com/site/about/guidelines.xhtml

BMJ Open

Prior exposure to antiretroviral therapy among adult patients presenting for HIV treatment initiation or re-initiation in sub-Saharan Africa: a systematic review

Journal:	*BMJ Open*
Manuscript ID	bmjopen-2022-071283.R2
Article Type:	Original research
Date Submitted by the Author:	12-Sep-2023
Complete List of Authors:	Benade, Mariet; Boston University School of Public Health, Department of Global Health; University of the Witwatersrand Johannesburg Faculty of Health Sciences, Health Economics and Epidemiology Research Office<br>Maskew, Mhairi; University of the Witwatersrand Johannesburg Faculty of Health Sciences, Health Economics and Epidemiology Research Office<br>Juntunen, Allison; Boston University School of Public Health, Department of Global Health<br>Flynn, David; Boston University School of Medicine<br>Rosen, Sydney; University of the Witwatersrand Johannesburg Faculty of Health Sciences, Health Economics and Epidemiology Research Office; Boston University School of Public Health, Department of Global Health
**Primary Subject Heading**:	HIV/AIDS
Secondary Subject Heading:	Global health, Health services research, Public health
Keywords:	EPIDEMIOLOGY, HIV & AIDS < INFECTIOUS DISEASES, PUBLIC HEALTH, Health Services Accessibility

SCHOLARONE™
Manuscripts

I, the Submitting Author has the right to grant and does grant on behalf of all authors of the Work (as defined in the below author licence), an exclusive licence and/or a non-exclusive licence for contributions from authors who are: i) UK Crown employees; ii) where BMJ has agreed a CC-BY licence shall apply, and/or iii) in accordance with the terms applicable for US Federal Government officers or employees acting as part of their official duties; on a worldwide, perpetual, irrevocable, royalty-free basis to BMJ Publishing Group Ltd ("BMJ") its licensees and where the relevant Journal is co-owned by BMJ to the co-owners of the Journal, to publish the Work in this journal and any other BMJ products and to exploit all rights, as set out in our licence.

The Submitting Author accepts and understands that any supply made under these terms is made by BMJ to the Submitting Author unless you are acting as an employee on behalf of your employer or a postgraduate student of an affiliated institution which is paying any applicable article publishing charge ("APC") for Open Access articles. Where the Submitting Author wishes to make the Work available on an Open Access basis (and intends to pay the relevant APC), the terms of reuse of such Open Access shall be governed by a Creative Commons licence – details of these licences and which Creative Commons licence will apply to this Work are set out in our licence referred to above.

Other than as permitted in any relevant BMJ Author's Self Archiving Policies, I confirm this Work has not been accepted for publication elsewhere, is not being considered for publication elsewhere and does not duplicate material already published. I confirm all authors consent to publication of this Work and authorise the granting of this licence.

For peer review only

Prior exposure to antiretroviral therapy among adult patients presenting for HIV treatment initiation or re-initiation in sub-Saharan Africa: a systematic review

Mariet Benade[a,b], Mhairi Maskew[b], Allison Juntunen[a], David Flynn[c], Sydney Rosen[a,b§]

[a]Department of Global Health, Boston University School of Public Health, Boston, MA, USA
[b]Health Economics and Epidemiology Research Office, Department of Internal Medicine, School of Clinical Medicine, Faculty of Health Sciences, University of the Witwatersrand, Johannesburg, South Africa
[c]Alumni Medical Library, Boston University, Boston, MA, USA

[§]Correspondence to:
Sydney Rosen
801 Massachusetts Ave Rm 390, Boston, MA 02118, USA
sbrosen@bu.edu

Email addresses:
MB: mbenade@bu.edu
MM: mmaskew@heroza.org
AJ: juntunen@bu.edu
DF: dflynn@bu.edu
SR: sbrosen@bu.edu

Running head: Proportion of non-naïve ART initiators in Africa

Word count: 4711 (main), 381 (abstract)

For peer review only

Abstract

Objectives

As countries have scaled up access to antiretroviral therapy (ART) for HIV, attrition rates of up to 30% annually have created a large pool of individuals who initiate treatment with prior ART experience. Little is known about the proportion of non-naïve re-initiators within the population presenting for treatment initiation.

Design

Systematic review of published articles and abstracts reporting proportions of non-naïve adult patients initiating ART in sub-Saharan Africa.

Data sources

PubMed, Embase Elsevier, Web of Science Core Collection, International AIDS Society conferences, CROI conferences.

Eligibility criteria

Clinical trials and observational studies; reporting on adults in sub-Saharan Africa who initiated lifelong ART; published in English between 1 January 2018 and 11 July 2023 and with data collected after January 2016. Self-report, laboratory discernment of antiretroviral metabolites, or viral suppression at initiation or in the medical record were accepted as evidence of prior exposure.

Data extraction and synthesis

We captured study and sample characteristics, proportions with previous ART exposure, and the indicator of previous exposure reported. We report results of each eligible study, estimate the risk of bias, and identify gaps in the literature.

Results

Of 2,740 articles, eleven, describing twelve cohorts contained sufficient information for the review. Proportions of initiators with evidence of prior ART use ranged from 5% (self-report only) to 53% (presence of ART metabolites in hair or blood sample). The vast majority of screened studies did not report naïve/non-naïve status. Metrics used to determine and report non-naïve proportions were inconsistent and difficult to interpret.

Conclusions

The proportion of patients initiating HIV treatment who are truly ART-naïve is not well documented. It is likely that 20% to 50% of ART patients who present for ART are re-initiators. Standard reporting metrics and diligence in reporting are needed, as is research to understand the reluctance of patients to report prior ART exposure.

Study registration
PROSPERO, CRD42022324136.

For peer review only

For peer review only - http://bmjopen.bmj.com/site/about/guidelines.xhtml

Keywords: Antiretroviral therapy, Treatment naïve, Africa, Systematic review, Initiation, Re-initiation

Strengths and limitations of this study

- Comprehensive review using multiple databases and conference abstract archives.
- Focused on recent data in the era since access to antiretroviral therapy for HIV became universal, increasing the potential for re-initiation of treatment.
- Utilized best practices to develop search string, with multiple reiterations based on initial findings.
- Recognized a wide range of reporting indicators for prior exposure to antiretrovirals medications.
- Limited by the lack of standardized reporting of prior antiretroviral exposure.

For peer review only

Introduction

The successful scale-up of access to antiretroviral therapy (ART) for HIV treatment in sub-Saharan Africa has produced a growing population of patients who have interrupted or stopped treatment sometime since they started, either permanently or temporarily. While very recent numbers on attrition from ART programs are scarce, retention in care rates for the region were reported to average 78% at 12 months after treatment initiation in a review published in 2015, suggesting that for a cohort of patients initiating in any given year, nearly a quarter have been lost from care one year later[1]. Many of these lost patients, however, proceed to "re-initiate" treatment in the months or years after dropping out of treatment programs[2]. Two estimates posit the extent of treatment re-initiation. The first, from the U.S. President's Emergency Plan for AIDS Relief (PEPFAR) reported that more than 580,000 patients returned to care after a treatment interruption in just the quarter from July to September 2020 in the countries that PEPFAR supports[3]. The second was from the Western Cape Province of South Africa, where among the subset of patients whose CD4 counts were less than 50 cells/mm^3, the proportion with prior treatment experience rose from 14% to 57% between 2008 and 2017[4].

Outside of indirect estimates such as those mentioned above, little is known about the actual proportions of non-naïve patients among all those presenting for ART initiation. Accurate data are difficult to obtain, largely because most HIV medical record systems neither distinguish between naïve and re-initiators nor allow tracking from one healthcare facility to another or over long intervals of inactivity. In most countries, a patient who originally initiated ART at one facility and then dropped out of care can easily present as a new patient at a nearby facility and be assumed to be ART-naïve. Self-reported information about a patient's naïve or non-naïve status may be unreliable, because patients who are known to have stopped treatment may be reprimanded, provided poorer service by healthcare facility staff, or required to participate in multiple adherence

For peer review only

For peer review only - http://bmjopen.bmj.com/site/about/guidelines.xhtml

training sessions, creating an incentive to present oneself as a new patient regardless of prior experience[5].

As the number of re-initiators continues to increase, understanding the proportion and characteristics of ART initiators who are not treatment-naïve is an important step in improving overall HIV treatment outcomes. By definition, most treatment re-initiators previously faced barriers to retention in care that they were unable to overcome in time or sufficiently to sustain continuity of treatment. Common barriers to retention include logistical challenges such as transport costs, psychosocial deterrents such as stigma, and personal preferences[6,7], and these barriers may become more prohibitive for patients who have already withdrawn from care once. Achieving long-term retention in care targets may thus require that healthcare systems differentiate interventions and services for re-initiators from those offered to naïve initiators. Re-initiators also comprise an increasing proportion of patients presenting with advanced HIV disease[4], who require additional care beyond simple ART initiation.

As national treatment programs mature, the proportion of ART initiators who are non-naïve will continue to grow, making the few available earlier estimates obsolete. To help fill the gap in empirical evidence on current proportions of naïve and non-naïve ART initiators, we conducted a systematic review of recently published or presented (2018 and later) peer-reviewed reports in sub-Saharan Africa that directly or indirectly presented data on re-initiation rates.

Methods

Guided by the Cochrane Handbook for Systematic Reviews of Interventions [8], we conducted a systematic review of peer-reviewed publications and published conference abstracts that reported on prior exposure to antiretroviral therapy among adult patients presenting for HIV treatment in sub-Saharan Africa. The review was registered on the International Prospective Register of

For peer review only

For peer review only - http://bmjopen.bmj.com/site/about/guidelines.xhtml

Systematic Reviews (PROSPERO; CRD42022324136) (Supplementary File S1). We report our findings in accordance to the Preferred Reporting Items for Systematic Reviews and Meta-Analyses (PRISMA) 2020 guidelines[9].

Search strategy, study selection, and data extraction

For this review, our primary outcome was the proportion of adults presenting for ART initiation (initially or after interruption) in public sector HIV treatment programs in sub-Saharan Africa who are not ART naïve. To indicate prior ART use, we accepted self-reported questionnaire responses, rates of viral load suppression at ART initiation (suggesting previous ART use), medical record evidence (e.g. a prior viral load test), and biological measurements of the presence of antiretroviral metabolites in blood, hair, or urine specimens at or prior to treatment initiation. Inclusion and exclusion criteria for the review are shown in Supplementary Table S1.

To identify potential sources, we developed a search string with the assistance of a medical librarian. The search syntax included variations of the terms HIV, treatment, antiretroviral therapy, retention, and adherence, limited to sub-Saharan Africa. During the course of the review, we made three revisions to the original protocol as submitted to PROSPERO, in order to maximize the potential for finding relevant sources. Specifically, we 1) included studies focusing on pregnant women starting ART in PMTCT programs; 2) added “undisclosed” and “retention” as search terms, as shown in Supplementary Table S2; and 3) included studies that required a minimum duration of ART for enrollment, as explained below. Search strings can be found in Supplementary Table S2.

We searched the PubMed, Embase (via Elsevier), and Web of Science Core Collection databases and published abstracts for the International AIDS Conference, International AIDS Society (IAS) Conference on HIV Science, and Conference on Retroviruses and Opportunistic Infections (CROI)

For peer review only - http://bmjopen.bmj.com/site/about/guidelines.xhtml

with a search string developed to identify English-language publications which reported on HIV treatment initiation in sub-Saharan Africa from 1 January 2018 until 11 July 2023. We further limited our search to articles from which the majority of the data were generated in 2016 or later, as this was when universal treatment access became common in the region and previous use became more likely. To capture the era of universal treatment access and allow time for guideline adoption, we considered studies published after 1 January 2018. Final searches conducted on 11 July 2023 updated our search phrases to include country names of countries in sub-Saharan Africa, using a pre-built search strategy created by the Canadian Health Libraries Association [10,11]. Searching for abstracts using Google Advanced search and limiting results to each conference's domain did not yield results, as such we scanned the abstract booklets for abstracts that reported on populations that were initiating ART treatment and searched conference archives with the key words "initiation," "naïve," "re-initiate," "newly," and "experience."

We included cohort, cross-sectional, case-control, and interventional studies that reported primary data on initiation of ART in the adult (≥18 years old) population, including for PMTCT. We included indexed pre-prints but excluded unpublished reports and publications in languages other than English. We also excluded commentaries, modeling studies, and other sources that did not report primary data. While we excluded systematic reviews and meta-analyses, we manually searched existing systematic reviews for additional, non-duplicate references to be included. Where more than one publication reported on the same patient cohort, we chose the one that was either most recent or provided the most relevant data.

Because we were interested specifically in reports of the proportion of patients presenting for ART initiation or re-initiation in routine care who were naïve and non-naïve, we excluded studies that stated that prior ART experience was an exclusion criterion for the study, with two exceptions. If a study included only participants who self-reported as naïve but were then found to have evidence of

For peer review only - http://bmjopen.bmj.com/site/about/guidelines.xhtml

previous antiretroviral (ARV) exposure, we included that proportion as a result, accepting that it explicitly omits patients who excluded themselves because of prior exposure and thus almost certainly reflects an underestimate of the true population prevalence of previous exposure. We also included studies that only enrolled patients who had achieved a specified duration of follow up on ART—whether one month or several years—despite the fact that these studies would have missed patients who were lost from care prior to reaching that specified duration.

All peer reviewed references identified using the respective search strings from PubMed, Embase and Web of Science were imported into Rayyan QCRI, where deduplication occurred. An initial, independent, blinded review (reviewers were not aware of each other's decisions) of the titles and abstracts was conducted by three study team members (MB, AJ, SR) using Rayyan QCRI. A full text review was then conducted for all publications remaining after the initial review by two study team members (MB, AJ or MB, SR), with conflicts resolved through discussion. Reasons for excluding publications were recorded during the full text review. As a quality check, one author (SR) also checked a sample (10%) of the excluded sources against exclusion criteria. At each stage of the review process any conflicts between reviewers were assessed and resolved though consensus of three authors (MB, AJ, SR). The results of the search were documented in accordance with the PRISMA-S reporting checklist (Supplementary File S2).

We created a data extraction tool to capture study and sample characteristics, proportions with previous ART exposure, and the type of indicator of previous exposure reported (e.g. self-report, laboratory results).

Outcomes and analysis

For peer review only

For peer review only - http://bmjopen.bmj.com/site/about/guidelines.xhtml

Our outcome of interest was the proportion of ART initiates who were treatment naïve at ART initiation, defined as a patient presenting for initiation of ART who has never previously taken antiretroviral therapy for treatment of HIV (“new initiator”), compared the proportion who were treatment-experienced at ART initiation, defined as a patient presenting for initiation of ART who had previously taken antiretroviral therapy for HIV treatment but had interrupted that therapy for a minimum of 3 months (“re-initiator”). We accepted each paper’s source of information about participants’ status: self-report, medical record review, viral suppression, or laboratory tests for ARV metabolites and report that source in our results.

To evaluate the data, we first report each paper’s outcome, with descriptive information regarding the population and setting to which the results apply. As described in the PROSPERO registration record, we had intended to estimate pooled results for individual countries and populations and to stratify by patient and facility characteristics, but we ultimately identified too few eligible sources to allow for any pooled or stratified analysis.

Quality of the eligible studies was assessed using the Joanna Briggs Institute Critical Appraisal Checklists[12].

Patient and public involvement

None.

Results

Sources identified

The results of the systematic search are shown in Figure 1. A total of 2,740 non-duplicate abstracts of peer reviewed journal articles and 9 abstracts from the selected conferences were screened. Systematic reviews that were screened are listed in Supplementary File S3. After the initial title and

For peer review only

abstract review, 2,361 articles and abstracts were excluded, leaving 379 documents for full-text review. During the full-text review, an additional 368 were excluded. Reasons for exclusions are reported in Supplementary Table S3. The primary reason for exclusion was lack of information on the naïve or non-naïve status of participants.

Eleven peer-reviewed articles were retained in the final data set for the full review, including one that reported data from two countries and will be included in our analysis as two studies, creating a total of twelve sets of results. The studies are described in Table 1.

Table 1. Studies included in the review

**Source (alphabetical by country)**	**Country and location**	**Study design and source of data**	**Study population**	**Dates of presentation for ART initiation**	**Sample size**	**Sex (% female)**	**Age (median, IQR)**
Lebelonyane 2020[13]	Botswana (national sample)	Subset of baseline data for intervention arm of cluster randomized trial of HIV prevention	Adults found to be HIV-positive during community HIV prevention campaign and linked to HIV care	June 2016-March 2018	800	55%	33 (26-41)
Buju 2022[14]	Democratic Republic of Congo (Bunia)	Observational prospective cohort of patients receiving dolutegravir	Adults presenting for ART initiation or ongoing ART treatment at all ART clinics in Busia	July 2019-July 2021 for those initiating during the study; earlier for those already on ART	177	69%	39 (12)*
Genet 2021 [15]	Ethiopia (Northwest Ethiopia)	Observational, cross sectional study of first line treatment failure	Adults† who had received at least 6 months of ART	May October 2017	430	58%	38 (12-67)**
Kunzweiler 2018 [16]	Kenya (Kisumu)	Observational prospective cohort	Adult men who have sex with men (MSM)	August 2015-September 2016	63	0%	27 (22-32)
Rosen 2019[17]	Kenya (Kericho, Kapsabet, and Kombewa counties)	Baseline data for intervention arm of clinical trial of same-day ART initiation	Adults presenting for ART initiation at 3 public sector hospitals	July 2017-April 2018	477	58%	36 (29-44)
Barnabas 2022 [18]	South Africa (KwaZulu Natal Province)	Baseline data from clinical trial of fee for ART home delivery	Adults living in study community and presenting for ART initiation	October 2019 – January 2020	6	46%[#]	36 (31-43)[#]

For peer review only

Source (alphabetical by country)	Country and location	Study design and source of data	Study population	Dates of presentation for ART initiation	Sample size	Sex (% female)	Age (median, IQR)
Dorward 2020[19]	South Africa (KwaZulu Natal Province)	Baseline data for randomized controlled trial for point-of-care HIV viral load testing	Adults clinically stable on ART and due for 6-month viral load testing	August 2016-February 2017 (est)	390	60%	32 (27-38)
Maskew 2020[20]	South Africa (Gauteng Province)	Baseline data for intervention arm in clinical trial of same-day ART initiation	Adults presenting for ART initiation at 3 public sector clinics	March-September 2018	296	64%	35 (30-44)
Mavhandu-Ramarumo 2019[21]	South Africa (Limpopo Province)	Baseline samples from clinical trial of drug resistance	Adults presenting for ART initiation at 3 public sector clinics	2017-2019	77	90%	35 (27-42)
Rosen 2019 [17]	South Africa (Gauteng Province)	Baseline data for intervention arm of clinical trial of same-day ART initiation	Adults presenting for ART initiation at 3 public sector clinics	March-July 2017	600	63%	34 (29-41)
Sithole 2021[22]	South Africa (KwaZulu Natal Province)	Subset of baseline data from clinical trial of home-based ART initiation	Non-pregnant adults presenting for ART initiation at 2 public sector clinics, with CD4 >100 and no active TB	February 2018-November 2018	193	60%	Not reported
Pry 2023[23]	Zambia (Lusaka)	Subset from implementation trial that utilized viral load testing at baseline	Adults diagnosed and initiating ART at 2 government facilities	May 2021 – March 2022	248	63%	30 (25-37)

*Mean (standard deviation)
†Study included children under 18, but they comprised <7% of the study sample
**Mean (minimum-maximum)
#Study enrolled larger sample of patients already on ART—only six were reported as initiating or re-initiating. Characteristics reported are for entire study population, not solely for subsample of six who initiated treatment.

Six of the studies in Table 1 were conducted in South Africa and one per country in Botswana, the Democratic Republic of Congo (DRC), Ethiopia, Kenya, and Zambia. Nine of the twelve reported baseline data from a clinical trial conducted for other purposes, while three were observational studies. All were very or somewhat small in size, enrolled adult patients presenting or having previously presented for routine ART initiation at public sector clinics, and, with the exception of Pry 2023, collected most or all data prior to the disruption in service delivery caused by COVID-19 in

For peer review only - http://bmjopen.bmj.com/site/about/guidelines.xhtml

early 2020[24]. Specific populations enrolled varied by study, though only one, Kunzweiler 2018, was limited to a non-general adult population.

Proportions of patients non-naïve

In Table 2, we report the proportion of patients in each study reported to be non-naïve when presenting for ART initiation.

Table 2. Proportions of cohorts reported to be non-naïve at ART initiation

Source	Source of data	Proportion non-naïve at initiation	Inclusion criteria related to prior ART exposure	Comments
Lebelonyane 2020*	Not stated; only indicated as participants who had "previous ART treatment"	54/800 (7%)	Sample included 16-17 year olds; minors may be less likely than adults to have had an opportunity for prior ART exposure**.	Participants were identified through community-based testing and referred for ART; sample does not represent routine walk-in ART initiation population.
Buju 2022	Viral load suppressed at ART initiation	93/177 (52%)	None.	Enrolled both initiators and patients already on ART; results presented are only for initiators.
Genet 2021	Self-report	90/430 (21%)	Sample included 12-17 year olds†; minors may be less likely than adults to have had an opportunity for prior ART exposure. Participants required to have completed 6 months on ART; patients lost to follow up before reaching 6 months were excluded.	Enrolled patients younger than 18. Those on second line ART were excluded; patients eligible for second line treatment may be more likely to be re-initiators.
Kunzweiler 2018	Viral load suppressed at ART initiation	19/63 (30%) (13 of 19 with suppressed viral load at ART initiation self-reported being ART-naïve)	Excluded 9 participants who were lost to follow up before reaching 6 months on ART.	Also excluded 3 patients who did not have viral load test results.
Rosen 2019	Self-report	18/240 (8%)	Included self-reported re-initiators who had interrupted ART for ≥ 3 months.	Patients who self-reported that they had previously initiated ART but had stopped for ≥3 months were eligible; those who self-reported that they had interrupted for < 3 months were excluded.
Barnabas 2022	Self-report and no existing record of ART at the study clinic	3/6 (50%)	Participants required to consent to a trial of payment for home ART; only clinically stable patients enrolled.	Enrolled both initiators and patients already on ART; results presented are only for initiators.

For peer review only

Source	Source of data	Proportion non-naïve at initiation	Inclusion criteria related to prior ART exposure	Comments
Dorward 2020	Self-report	18/390 (5%)	Participants required to have completed 6 months on ART; patients lost to follow up before reaching 6 months were excluded.	
Maskew 2020	Self-report	33/296 (11%)	Included self-reported re-initiators who had interrupted ART for ≥ 3 months	Patients who self-reported that they had previously initiated ART but had stopped for ≥3 months were eligible; those who self-reported that they had interrupted for < 3 months were excluded.
Mavhandu-Ramarumo 2019	Laboratory assay of blood and/or hair sample for presence of TDF, EFV, and/or FTC metabolites	41/77 (53%)	All participants self-reported as ART naïve	Stated enrollment criteria included naivete; those who had been on ART previously may have self-screened out of this study.
Rosen 2019	Self-report	7/298 (2%)	Included self-reported re-initiators who had interrupted ART for ≥ 3 months	Patients who self-reported that they had previously initiated ART but had stopped for ≥3 months were eligible; those who self-reported that they had interrupted for < 3 months were excluded.
Sithole 2021††	Undetectable viral load at ART initiation	62/193 (32%) (Other outcomes reported: 42/193 (22%) had medical record evidence of prior ART use; 37/193 (19%) had detectable ARV metabolites in blood.)	All participants self-reported as ART naïve	Stated enrollment criteria included naivete; those who had been on ART previously may have self-screened out of this study. Participants were identified through community-based testing; sample does not represent routine walk-in ART initiation population. Primary DO-ART trial excluded 588 participants out of 2,479 who were virally suppressed at time of initiation and 66 who were found to already be on ART[25]
Pry 2023	Undetectable viral load at ART initiation	66/248 (27%) (Other outcomes reported: among 57 participants with suppressed VL who completed survey on prior ART use, 14% reported previous ART)	None	Survey on silent transfers (self-reported prior ART use) was conducted at a follow-up visit after it was determined that participants had a suppressed viral load at initiation.

281 TDF, Tenofovir; EFV, Efavirenz; FTC, Emtricitabine
282 *A separate publication by the same author team, reporting data that overlapped with those reported in this study, stated that "22% of
283 advanced HIV patients had previously been on ART." [26]
284 **16-17 year olds receive treatment as adults in this setting.
285 †Minors under age 18 comprised <7% of the study sample.
286 ††The parent study on which this study was based.
287
288 The proportion of patients presenting for ART initiation who were reported to be non-naïve ranged
289 from 2 to 53%. It is important to note that the results shown in Table 2 are not strictly comparable to

For peer review only - http://bmjopen.bmj.com/site/about/guidelines.xhtml

one another, however, due to the different data sources, populations, and relevant exclusions listed in the right-hand column of the table. In Maskew 2021 and Rosen 2020, non-naïve patients were allowed to enroll as long as they had been off ART for at least three months. Nevertheless, these studies, which are the only ones that relied entirely on participant self-report, reported the lowest proportion of non-naïve participants. Barnabas 2023, which used both self-report and the facility's medical records, reported a much higher proportion with prior ART experience (50%) in the study's very small sample of initiators; rates varied between 33% and 66% previous exposure in the control and intervention arms, but that difference comprised only one individual in absolute terms. Two other studies in Table 2, Mavhandu-Ramarumo 2019 and Sithole 2021, explicitly excluded self-reported non-naïve patients; all patients enrolled in these studies claimed not to have been on ART previously. The relatively high proportions of non-naïve patients in these studies are thus still minimum estimates of the true proportion of ART initiators with prior treatment exposure at the study clinics, as anyone who admitted to prior use of ARVs will have been excluded from study enrolment. Similarly, Kunzweiler 2018 and Dorward 2020 excluded patients lost to follow up before 6 months after ART initiation, and thus may have underestimated the proportion re-initiating if non-naïve patients are more likely than naïve patients to drop out of care. Study participants were also drawn from diverse populations of individuals with HIV, including those identified in a community campaign (Lebelonyane 2020), in a household survey as part of a randomized trial (Sithole 2021), or as routine, walk-in presenters at clinics (Buju 2022, Maskew 2020, Rosen 2019).

Because all the eligible studies were small and/or had primary outcomes other than the proportion naive, little stratification of results by facility or patient characteristics was included. Mavhandu-Ramarumo 2019 reported that 7 of the 8 (88%) males in the sample population had evidence of prior ART exposure, compared to only 49% of the 34 females. In contrast, Sithole 2021 found that women (37%) were more likely to have evidence of undisclosed ART use than men (25%). Other characteristics that were associated with undisclosed ART use by undetectable viral load were

For peer review only - http://bmjopen.bmj.com/site/about/guidelines.xhtml

younger age (35% in 18-29-year-olds, 30% in 30-49 year olds, and 23% among those >50 years) and living with a partner who was HIV positive (44% compared to 37%; adj OR 1.94 (95% CI 0.95-3.96)). Kunzweiler 2018's results apply specifically to MSM. Pry 2023 found that women ≥40 years had the highest probability of being non-naïve (42% 95%CI: 39.3-44.3%), while men aged 18-24 years had a baseline probability of just 12% (95% CI: 4.6-19.0%). Age was associated with higher rates of viral suppression, even when adjusting for sex, marital status, education, or facility of initiation. Being married and being female were both also associated with significantly increased adjusted prevalence ratios. Other studies did not provide information for stratification by facility or patient characteristics.

Quality of evidence

Each of the studies included in the review either presented baseline enrollment data from a randomized controlled trial or observational data. Outcomes after ART initiation were not relevant for our review, which looked only at the status of patients at baseline (ART initiation). For Maskew 2020 and Rosen 2019, data on self-reported naïve status were limited to the intervention arm. The setting, inclusion criteria, and baseline characteristics for these studies were clearly defined. Mavhandu-Ramarumo 2019, Lebelonyane 2020, Dorward 2020, Barnabas 2022, and Sithole 2021 also sufficiently defined inclusion criteria, study setting, and baseline characteristics.

We assessed Kunzweiler 2018, Genet 2021, Buju 2022, and Pry 2023 as observational studies. Kunzweiler 2018 utilised snowball sampling, as is frequently done among key populations at high risk of stigma. All four papers described the study setting and sample population of interest clearly, including age, sex and clinical characteristics of HIV presentation.

For peer review only - http://bmjopen.bmj.com/site/about/guidelines.xhtml

As is indicated in Table 2 and discussed further below, each of the studies included in this review used a different indicator of prior ART exposure (non-naivete), and most had limitations as to their accuracy and/or the representativeness of their populations.

Discussion

We systematically reviewed peer-reviewed evidence on the proportions of patients presenting for treatment initiation in sub-Saharan Africa who are or are not ART-naïve. The proportions non-naïve in the sources we found ranged from a low of 2% using self-report only to a high of 53% based on a laboratory analysis of ARV metabolites in blood and hair samples of patients who self-reported to be naïve.

Perhaps the most striking finding of this review is the sheer lack of published evidence to answer our research question. Despite a comprehensive search of the literature published between 2018 and 2022, and including data since 2016, we identified only eleven sources and twelve cohorts that reported this information, and most included it in only in passing. Half the studies were conducted in South Africa and were relatively small in size; most of those from other countries provided very little detail. Based on the published and presented research alone, it is fair to say that very little is known about the true proportion of ART initiators who are not treatment-naïve in South Africa, and almost nothing is known about the rest of the region or about specific sub-populations or risk groups. While it is possible that more information is available to program managers who have access to routinely collected medical record data, nothing in the literature suggests that such information is being generated on a large scale or, more important, utilized for program improvement.

During our search, we made a concerted effort to find additional eligible sources, in the hope that there would be more data to review and analyze. This included adding additional search terms,

For peer review only

including data on pregnant and post-partum women, and reviewing reference lists from relevant systematic reviews. We reviewed an unusually large number of full-text manuscripts in the hope that they would include proportions naïve and non-naïve in their cohort descriptions (typically "Table 1") even though there was no indication of this in the abstract.

Unfortunately, most of the sources that originally appeared promising were found to be ineligible, for various reasons. Most simply did not report on baseline naïve/non-naïve status at ART initiation, using any indicator. Some intentionally excluded non-naïve patients prior to enrolment and then reported all participants as naïve without further investigation. For studies that explicitly screened out non-naïve participants, we considered calculating proportions non-naive based on reported numbers of potential participants included and excluded, but we realized that many studies only applied the non-naïve criterion after screening potential participants out for other reasons. We thus could not safely rely on numbers screened out due to non-naïve status for our numerator and had to exclude those papers. Even some of the papers we did include either provided only a passing reference about prior ART exposure, making us uncertain that we interpreted them correctly (e.g. Lebelonyane 2020), or explicitly excluded patients lost to follow up in the early treatment period (e.g. Kunzweiler 2018 and Dorward 2020). Finally, several studies were excluded because we could not interpret the data reported or had doubts about the accuracy of prior exposure data based on the data sources used, even beyond the limitations discussed below.

For peer review only

The small number of eligible papers we did find offer some useful information. They used several different indicators for identifying non-naïve patients, and the indicators produced results that are consistent with their expected accuracy. Maskew 2020 and Rosen 2019, which relied solely on self-report, and Lebelonyane 2020, for with the source of data is unknown, found the lowest proportions non-naïve. Sithole 2021, which excluded a priori anyone self-reporting prior utilization, reported that 32% of patients had evidence of prior use based on being virally suppressed. Buju 2022 and

For peer review only - http://bmjopen.bmj.com/site/about/guidelines.xhtml

Kunzweiler 2018 reported that 52% and 30%, respectively, of patients presenting for initiation already had suppressed viral loads, suggesting prior ARV use. Mavhandu-Ramarumo 2019, which also excluded a priori anyone admitting prior utilization, using the most rigorous methodology with both blood and hair samples, estimated 53% of patients had prior ART exposure. The large observed difference between males and females and the small number of males in this study, however, suggest caution in applying the results to male patients. The expectation of viral rebound within 4 weeks of treatment interruption[27] suggests that viral suppression underestimates the true proportion non-naïve, as suppression as an indicator only captures recent treatment interrupters. Similarly, metabolite tests capture a maximum of about 90 days' prior exposure; anyone who interrupted treatment more than three months prior to study enrollment would be missed in the count of non-naïve initiators [27].

Based on results of the studies that used laboratory tests, we assume that those that relied on self-report alone—e.g. Maskew 2020, Rosen 2019, and Dorward 2020—underestimated the true proportion of participants who were non-naïve at initiation. Barnabas 2023, which combined self-report and same-facility record review, is an outlier among the studies using self-report, but the very small sample size of initiators (n=6) suggests caution in drawing conclusions from it. While study inclusion criteria may have biased the samples in Sithole 2021 and Mavhandu-Ramarumo 2019, these, together with Buju 2022, Kunzweiler 2018, Genet 2021, and Pry 2023 suggest that it is reasonable (and conservative) to conclude that between 20% and 50% of ART patients—and likely at least 30%--who present for ART are re-initiators. This proportion can be expected to increase with each passing year, as the number of truly naïve HIV-positive individuals declines. If this is so, then re-initiators comprise an important sub-population whose needs are likely to differ from those of naïve initiators and to whom service delivery should be tailored. This is especially important as recent evidence shows that mortality is significantly higher among PLHIV who interrupt and then reinitiate treatment, especially if the interruption occurred within 6 months of ART initiation.[28]

For peer review only

For peer review only - http://bmjopen.bmj.com/site/about/guidelines.xhtml

As is evident from the discussion above, this review had several limitations. First, while we believe that our search of the peer-reviewed, published literature and abstracts was thorough, the lack of standard terminology for describing prior ART exposure hampered the creation of precise search strings, and it is possible that some sources were missed. Second, we found information from only six of sub-Saharan Africa's 46 countries, and six of our twelve observations were from a single country, South Africa. Since each country in sub-Saharan Africa has a different experience with attrition from ART and approach to re-initiation, results may not be generalizable. Third, as explained in the introduction, even such data as are available tend to be incomplete, due to the limitations of self-reporting and of existing medical record systems.

Fourth, the wide range of results identified may reflect study methodologies, but it may also indicate substantial geographic diversity in outcomes that we cannot address with the data available. We speculate that additional relevant data are collected by program managers, ministries of health, and others but are either not analyzed to answer our research question or simply not published and therefore not accessible. Fifth, the small number of eligible sources, small sample sizes, and heterogeneity of research methods made it impossible to aggregate the results or produce meaningful summary statistics, beyond the range discussed above. Sixth, recall bias may be present in the studies that asked participants after six months or more on ART to self-report their naïve/non-naïve status at the time they initiated treatment. Seventh, none of the papers included reported the duration of the interval between a participant's prior ART experience and re-initiation. Some apparent re-initiators may in fact reflect unrecorded ("silent") transfers from one facility to another, without a medication interruption in between. This phenomenon may help explain the high proportion of "initiating" patients who already have viral suppression in Buju (2022), for example.

For peer review only - http://bmjopen.bmj.com/site/about/guidelines.xhtml

Finally, the fact that nine of 12 cohorts included were from clinical trials that provided patient compensation may have biased enrollments, though we cannot know how this may have affected patient enrolment or self-report of prior ART usage and the subsequent direction of the bias.

In addition to the sheer dearth of information available to answer our question, the search reported here revealed three important research priorities. First, there is a need for a standard terminology to describe patients with prior ART exposure and prior ART initiation experience. Even the binary terms "naïve" and "non-naïve" can be unclear if patients have previously used antiretroviral medications for PrEP or prevention of vertical transmission. Terms such as "ART experienced" and "ART exposed" are often substituted for non-naïve, without specification of what they refer to. Prior ART use may be "disclosed" or "self-reported." Similarly, the duration of treatment interruption that leads to "re-initiation" is rarely specified, and "re-initiation" and "re-engagement" are used interchangeably. We can assume that patient returning to care after an interruption of less than one month is not likely to be regarded as a re-initiator, and a patient returning after an interruption of more than one year will likely be defined as a re-initiator. But what of patients with interruptions of 6 or 8 months? A common terminology for describing the phenomenon addressed here would be of great assistance in understanding its magnitude.

Second, in view of the potentially very high proportion of re-initiators among "new" ART patients, it is critical that researchers begin to report proportions naïve and non-naïve as a standard variable when describing patient cohorts, even if data come solely from self-report. We cannot determine from the literature whether many studies do collect this information but omit it from their reports or if it has simply not been collected. We identified several papers that came close to indicating a proportion non-naïve but did not explain their findings clearly enough to include in this review, suggesting that many studies do indeed have access to the relevant information. The study mentioned in our introduction from South Africa's Western Cape Province, for example, provided

For peer review only

For peer review only - http://bmjopen.bmj.com/site/about/guidelines.xhtml

469 detailed information about patients with advanced HIV disease and suggested that more than a third
470 of all patients with very low CD4 cell counts had previously been on ART but were now off, but we
471 could not calculate the overall proportion of non-naïve initiators from the data reported [4]. In
472 earlier years, when the proportion of non-naïve patients was low because treatment programs were
473 still rapidly expanding, the question of prior ART experience may not have been a priority. In view of
474 the results of the few studies available, it is clearly a priority now, for several reasons. First,
475 retention in care will remain a challenge if those who disengaged from care previously remain at
476 higher risk of disengaging from care again[29], unless the obstacles to continuity in care have been
477 identified and addressed. Second, if service delivery needs do differ between ART naïve and ART
478 experienced clients, treatment outcomes may also be affected by the large number of non-naive
479 ART clients accessing services. Third, to the extent that resistance to first-line antiretroviral
480 medications remains a concern, non-naïve patients may face higher risks of poorer responses to
481 these medications after re-initiation.
482
483 Finally, the phenomenon of large numbers of patients who decline to reveal prior ART use, even
484 when asked directly, is concerning. For studies that intend to limit participation to naïve patients,
485 stated exclusion of those who are non-naïve may encourage non-disclosure, to avoid being denied
486 study enrolment on this basis. Even so, it appears likely that many patients opt to lie about prior
487 exposure. Other research suggests that they have good reason for doing so, as clinics may refuse to
488 re-initiate those who admit to prior default and/or may provide poorer service to them[30–32].
489 Creating a clinic and community atmosphere that promotes honesty about prior exposure should
490 also be a priority.
491
492 In conclusion, while we recognize that simply knowing the proportion of non-naïve patients in a
493 given population will not in itself improve the quality of service delivery, measuring the size of the
494 problem is a critical step in creating momentum to development and implement interventions

For peer review only - http://bmjopen.bmj.com/site/about/guidelines.xhtml

targeted specifically at re-initiators. Since these are patients who have already demonstrated that they face obstacles to remaining in care, identifying and targeting them for appropriate services is a vital step in improving the outcomes of treatment programs.

Supplemental files

Supplementary file S1. Search protocol (PROSPERO)

Supplementary file S2. PRISMA checklists for publications and abstracts

Supplementary file S3. Systematic reviews screened for potential articles

Supplementary Table S1. Inclusion/exclusion criteria for publications and abstracts

Supplementary Table S2. Search strategy

Supplementary Table S3. Reasons for exclusion after full-text review

Ethics approval and consent to participate

Not applicable; this is a systematic review of published information and has no human participants.

Data availability statement

All data relevant to the study are included in the article or uploaded as supplementary information.

Competing interests

The authors declare that they have no competing interests.

Funding

Funding for the study was provided the Bill & Melinda Gates Foundation through INV-031690 to Boston University. The funder had no role in study design, data collection and analysis, decision to publish, or preparation of the manuscript.

Contributors

MM, SR, and MB conceived of and designed the study. DF, MB and SR created the search strategy. DF conducted the searches. DF conducted the searches. MB, SR, and AJ identified and reviewed sources and extracted data. MB and SR analyzed the data and drafted the manuscript. All authors reviewed and edited the manuscript.

References

For peer review only

For peer review only - http://bmjopen.bmj.com/site/about/guidelines.xhtml

1. Fox MP, Rosen S. Retention of adult patients on antiretroviral therapy in low- and middle-income countries: systematic review and meta-analysis 2008–2013. J Acquir Immune Defic Syndr 2015;69: 98. doi:10.1097/QAI.0000000000000553
2. Ehrenkranz PD, Rosen S, Boulle A, Eaton J, Ford N, Fox M, et al. The revolving door of HIV care : Revising the service delivery cascade to achieve the UNAIDS 95-95-95 goals. PLoS Med 2021;18: e1003651. doi:10.1371/journal.pmed.1003651
3. Godfrey K. Supporting reengagement: What's new in DSD for HIV treatment: from WHO recommendations to reality. IAS 2021, July 18-21, 2021. Available from: https://differentiatedservicedelivery.org/Portals/0/adam/Content/TlGQJAsI5UqL5SHACJkSPg/File/IAS2021_treatmentcontinuity_IAS%20inputs%2030June.pdf. Accessed 19 Oct 2022.
4. Osler M, Hilderbrand K, Goemaere E, Ford N, Smith M, Meintjes G, et al. The continuing burden of advanced HIV disease over 10 years of increasing antiretroviral therapy coverage in South Africa. Clin Infect Dis 2018;66: S118–S125. doi:10.1093/cid/cix1140
5. Bisnauth MA, Davies N, Monareng S, Buthelezi F, Struthers H, McIntyre J, et al. Why do patients interrupt and return to antiretroviral therapy? Retention in HIV care from the patient's perspective in Johannesburg, South Africa. PLoS One 2021;16: 1–15. doi:10.1371/journal.pone.0256540
6. Mody A, Sikombe K, Beres LK, Simbeza S, Mukamba N, Eshun-Wilson I, et al. Profiles of HIV care disruptions among adult patients lost to follow-up in Zambia. J Acq Immun Defic Syndr 2021; 86: 62–72. doi:10.1097/qai.0000000000002530
7. Sikazwe I, Eshun-Wilson I, Sikombe K, Beres LK, Somwe P, Mody A, et al. Patient-reported reasons for stopping care or switching clinics in Zambia: A multisite, regionally representative estimate using a multistage sampling-based approach in Zambia. Clin Infect Dis 2021;73: e2294–e2302. doi:10.1093/cid/ciaa1501
8. Higgins JPT, Thomas J, Chandler J, Cumpston M, Li T, Page MJ, Welch VA (editors). *Cochrane Handbook for Systematic Reviews of Interventions* version 6.3 (updated March 2022). Cochrane, 2022. Available from www.training.cochrane.org/handbook.
9. Page MJ, McKenzie JE, Bossuyt PM, Boutron I, Hoffmann TC, Mulrow CD, et al. The PRISMA 2020 statement: An updated guideline for reporting systematic reviews. BMJ 2021;372.
10. ISSG Search Filters Resource [Internet]. 2023. Available from: https://sites.google.com/a/york.ac.uk/issg-search-filters-resource/home. Accessed 7 August 2023.
11. Joanna Briggs Institute. Critical Appraisal Tools. Available from : https://jbi.global/critical-appraisal-tools. Accessed 6 Sep 2022.
12. Lebelonyane R, Bachanas P, Block L, Ussery F, Abrams W, Roland M, et al. Rapid antiretroviral therapy initiation in the Botswana Combination Prevention Project: a quasi-experimental before and after study. Lancet HIV 2020;7: e545–e553. doi:10.1016/S2352-3018(20)30187-9
13. Buju RT, Akilimali PZ, Tran N-T, Kamangu EN, Mesia GK, Kayembe JMN, et al. Determinants of survival of HIV patients receiving dolutegravir: a prospective cohort study in conflict-affected Bunia, Democratic Republic of Congo. Int J Environ Res Public Health 2022;19. doi:10.3390/ijerph191610220
14. Genet A, Mekonnen Z, Yizengaw E, Mekonnen D. First line antiretroviral treatment failure and associated factors among people living with HIV in northwest Ethiopia. Afr Health Sci 2021;21: 263–272. Available: https://pubmed.ncbi.nlm.nih.gov/34394306/
15. Kunzweiler CP, Bailey RC, Mehta SD, Okall DO, Obondi E, Djomand G, et al. Factors associated with viral suppression among HIV-positive Kenyan gay and bisexual men who have sex with men. AIDS Care 2018;30: S76–S88. doi:10.1080/09540121.2018.1510109
16. Rosen S, Maskew M, Larson BA, Brennan AT, Tsikhutsu I, Fox MP, et al. Simplified clinical algorithm for identifying patients eligible for same-day HIV treatment initiation (SLATE): Results from an individually randomized trial in South Africa and Kenya. PLoS Med 2019;16: e1002912. doi:10.1371/journal.pmed.1002912

For peer review only - http://bmjopen.bmj.com/site/about/guidelines.xhtml

17. Barnabas RV, Szpiro AA, Ntinga X, Mugambi ML, van Rooyen H, Bruce A, et al. Fee for home delivery and monitoring of antiretroviral therapy for HIV infection compared with standard clinic-based services in South Africa: a randomised controlled trial. Lancet HIV 2022;9(12):e848–56.
18. Dorward J, PK D, Osman F, Sookrajh Y, Pillay M, Moodley P, et al. Short communication: early antiretroviral therapy is associated with better viral suppression and less HIV drug resistance after implementation of universal treatment in South Africa. AIDS Res Hum Retroviruses 2020;36: 297–299. Available: https://pubmed.ncbi.nlm.nih.gov/31663368/
19. Maskew M, Brennan AT, Fox MP, Vezi L, Venter WDF, Ehrenkranz P, et al. A clinical algorithm for same-day HIV treatment initiation in settings with high TB symptom prevalence in South Africa: The SLATE II individually randomized clinical trial. PLoS Med 17(8): e1003226. https://doi.org/10.1371/journal.pmed.1003226
20. Mavhandu-Ramarumo LG, Tambe LAM, Matume ND, Katerere D, Bessong PO. Undisclosed exposure to antiretrovirals prior to treatment initiation: An exploratory analysis. South Afr J HIV Med 2021;22: 1–10. doi:10.4102/sajhivmed.v22i1.1200
21. Sithole N, Gunda R, Koole O, Krows M, Schaafsma T, Moshabela M, et al. Undisclosed antiretroviral therapy use at primary health care clinics in rural KwaZulu Natal South Africa: A DO-ART trial sub-study. AIDS Behav 2021. doi:10.1007/s10461-021-03319-4
22. Pry JM, Mwila C, Kapesa H, Mulabe M, Frimpong C, Moono M, et al. Estimating potential silent transfer using baseline viral load measures among people presenting as new to HIV care in Lusaka, Zambia: A cross-sectional study BMJ Open. 2023;13(5):1–8.
23. Benade M, Long L, Rosen S, Tucker J. Reduction in initiations of HIV treatment in South Africa during the COVID pandemic. BMC Health Serv Res 2022;22: 428. doi:10.1186/s12913-022-07714-y
24. Barnabas R, AA S, H van R, Asiimwe S, Pillay D, NC W, et al. Community-based antiretroviral therapy versus standard clinic-based services for HIV in South Africa and Uganda (DO ART): a randomised trial. Lancet Glob Health 2020;8: e1305–e1315. doi:10.1016/S2214-109X(20)30313-2
25. Lebelonyane R, Mills LA, Mogorosi C, Ussery F, Marukutira T, Theu J, et al. Advanced HIV disease in the Botswana combination prevention project: prevalence, risk factors, and outcomes. AIDS 2020;34:2223–30.
26. Mdluli T, Li Y, Pinyakorn S, Reeves DB, Cardozo-Ojeda EF, Yates A, et al. Acute HIV-1 infection viremia associate with rebound upon treatment interruption. Med. 2022;3(9):622-635.e3.
27. Anderson PL, Liu AY, Castillo-Mancilla JR, Gardner EM, Seifert SM, Mchugh C, et al. Intracellular Tenofovir-Diphosphate and Emtricitabine-Triphosphate in Dried Blood Spots following Directly Observed Therapy. Antimicrob Agents Chemother 2018;62:1–13.
28. Moolla H, Davies M, Davies C, Euvrard J, Prozeskry H, Fox M, et al. The effect of unplanned care interruptions on the mortality of adults resuming antiretroviral therapy in South Africa: a survival analysis. 12th International AIDS Society Conference on HIV Science, Brisbane, abstract OAC0104, 2023.
29. Inzaule SC, Kroeze S, Kityo CM, Siwale M, Akanmu S, Wellington M, et al. Long-term HIV treatment outcomes and associated factors in sub-Saharan Africa: multicountry longitudinal cohort analysis. AIDS 2022; 36:1437–47.
30. Bisnauth MA, Davies N, Monareng S, Buthelezi F, Struthers H, McIntyre J, et al. Why do patients interrupt and return to antiretroviral therapy? Retention in HIV care from the patient's perspective in Johannesburg, South Africa. PLoS ONE 2021; 16:e0256540. doi:10.1371/journal.pone.0256540
31. Rees K, Bisnauth M, O'Connor C, Ramvhulela T, Vali N. Understanding patients reinitiating antiretroviral therapy in two South African districts. South Afr J HIV Med 2022;23. doi:10.4102/sajhivmed.v23i1.1380

For peer review only

For peer review only - http://bmjopen.bmj.com/site/about/guidelines.xhtml

630 32. Bisnauth MA, Davies N, Monareng S, Struthers H, McIntyre JA, Rees K. Exploring healthcare workers' experiences of managing patients returning to HIV care in Johannesburg, South Africa. Glob Health Action 2022;15. doi:10.1080/16549716.2021.2012019.

Figure 1. PRISMA flowchart

For peer review only

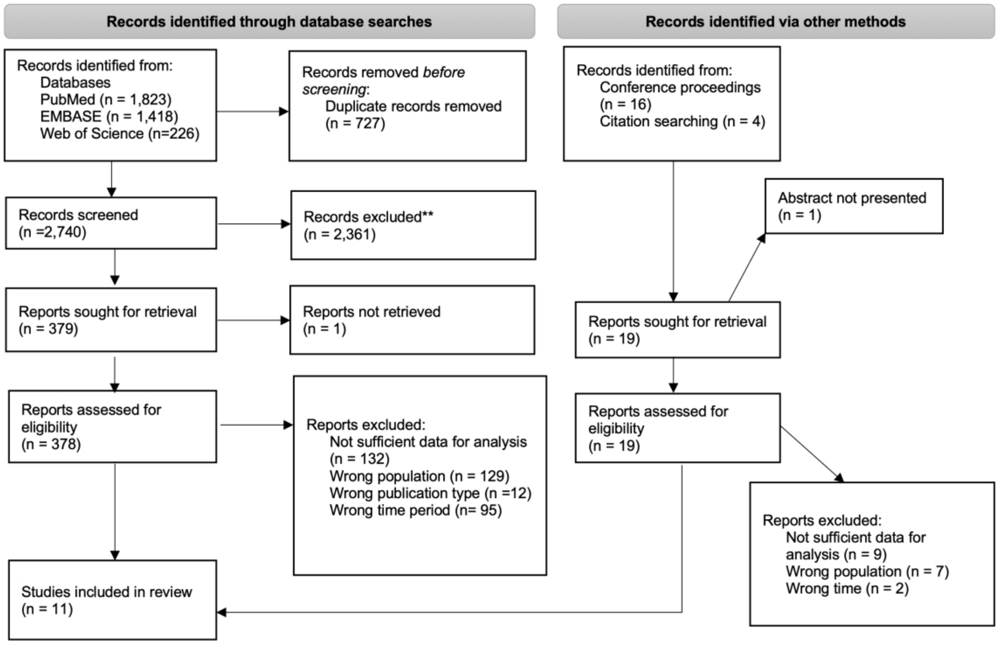

Prisma flow chart

893x573mm (72 x 72 DPI)

NIHR | National Institute for Health Research

PROSPERO
International prospective register of systematic reviews

To enable PROSPERO to focus on COVID-19 submissions, this registration record has undergone basic automated checks for eligibility and is published exactly as submitted. PROSPERO has never provided peer review, and usual checking by the PROSPERO team does not endorse content. Therefore, automatically published records should be treated as any other PROSPERO registration. Further detail is provided here.

Citation

Mariet Benade, Mhairi Maskew, Linda Sande, Nancy Scott, Allison Juntunen, Sydney Rosen. Prior exposure to antiretroviral therapy among adult patients presenting for HIV treatment initiation or re-initiation in sub-Saharan Africa: a systematic review. PROSPERO 2022 CRD42022324136 Available from: https://www.crd.york.ac.uk/prospero/display_record.php?ID=CRD42022324136

Review question

What proportions of adult patients presenting for ART initiation or re-initiation in public sector HIV treatment programs in Southern Africa are (a) ART naïve (new initiators) and (b) treatment experienced (re-initiators)?

Searches

We will search PubMed, Embase, and Web of Science and abstracts from the International AIDS Conference, International AIDS Society (IAS) Conference on HIV Science, and Conference on Retroviruses and Opportunistic Infections (CROI). We will manually search reference lists from sources identified in the search. Unpublished studies (e.g. preprints) will be included. Searches will be limited to English language publications.

The search period will be from January 1, 2018 to March 31, 2022. Inclusion in the review will be limited to sources for which a majority of individual-level data pertain to 2016 or later, as 2016 was the year when universal treatment access became common in sub-Saharan Africa. If sources report data from both before and after January 1, 2016, only results pertaining to the data from after that date will be used.

The search will be re-run prior to final analysis to identify very recent reports.

Types of study to be included

Studies that report initiation of ART for adult patients. These can be cohort, case control, cross-sectional, or interventional studies. White papers, commentaries, modeling studies, cost-effectiveness studies, and case studies will be excluded. Qualitative studies will be excluded unless there is specific data on treatment experience at ART initiation. Existing systematic reviews will be evaluated for additional, non-duplicate references. Published protocols with no data will be excluded, but if the study meets the inclusion criteria, we will attempt to find study results to evaluate for inclusion.

Condition or domain being studied

The successful scale-up of access to ART for HIV in sub-Saharan Africa has produced a growing population of patients who have interrupted or stopped treatment, and are now “re-initiating” therapy. While a diminishing number utilized ART temporarily for prevention of mother-to-child transmission prior to universal eligibility for ART, most are patients who stopped treatment, despite guidelines recommending lifelong use. Estimates of the proportion of patients presenting for ART re-initiation range from 10% to 50%.

Treatment re-initiators previously faced barriers to retention in care that they were unable to overcome. Achieving long-term retention in care targets may require that healthcare systems differentiate interventions and services for re-initiators from those offered to naïve initiators.

A first step in improving retention in care for ART re-initiators is to estimate how many individuals there are in any population of patients presenting for ART initiation. Few such estimates are available, largely because most healthcare data systems do not distinguish between naïve and re-initiators, allow tracking from one

For peer review only - http://bmjopen.bmj.com/site/about/guidelines.xhtml

healthcare facility to another or over long intervals of inactivity. To provide a baseline for considering this challenge, we'll search the recent literature for estimates of the proportion of patients who are re-initiators in sub-Saharan Africa.

Participants/population

Inclusion: Adults (?18 years of age) living with HIV presenting for initiation of any regimen of lifelong antiretroviral treatment in any sub-Saharan African country.
Exclusion: Individuals presenting for initiation of antiretroviral medications for any reason other than therapeutic (e.g. PrEP, PMTCT).

Intervention(s), exposure(s)

Initiation of ART is the intervention of interest. This will be an observational study of the characteristics of individuals initiating ART for HIV treatment (specifically, whether or not they have prior exposure to ART at the time of initiation).

Comparator(s)/control

Not applicable. Eligible studies need only report the proportions of patients who are treatment naïve and those who are treatment experienced.

Context

Initiation of ART in routine care settings in sub-Saharan Africa, including any type of facility or provider.

Main outcome(s)

Proportion of patients who are:
a. Treatment naïve at ART initiation, defined as a patient presenting for initiation of ART who has never previously taken antiretroviral therapy for treatment of HIV (new initiator); or
b. Treatment experienced at ART initiation, defined as a patient presenting for initiation of ART who has previously taken antiretroviral therapy for HIV treatment but has interrupted that therapy for a minimum of 3 months (re-initiator).

Measures of effect

Proportion, odds ratio

Additional outcome(s)

If data allow, we will also estimate the primary outcomes stratified by patient and/or facility characteristics, such as patient age and sex and facility country and setting.

Measures of effect

Proportion

Data extraction (selection and coding)

Study selection:

Rayyan will be used to support screening of studies and track inclusion or exclusion of studies. References will be managed in Mendeley, and duplicates will be removed. Two independent reviewers will be involved in each phase of review, including title and abstract screening, full-text screening, and data extraction. Title and abstract screening will include a pilot test to assess if the reviewers are able to achieve over 90% agreement on inclusion based on the eligibility criteria listed in this protocol. Disagreements will be resolved through discussion to achieve consensus. After full text screening, relevant non-duplicate citations from previous systematic reviews and other studies labelled for snowball in the initial search will be added for inclusion/exclusion consideration in a snowball phase.

For peer review only

Data extraction:

Data extraction will use a pre-determined template that captures all relevant variables. Data extraction will be done independently by two reviewers and discrepancies will be resolved by consensus. Data items to be extracted from studies include:

- Country
- District or locality
- Facility type
- Facility setting (urban, rural)
- Study year(s) or period(s) cohort observed
- Length of patient follow-up
- ART guidelines or protocol(s) in use during study period
- Study/cohort sample size
- Patient characteristics, including:
o Age distribution
o Gender distribution
o Clinical indicators of disease stage such as presenting CD4 count or WHO stage
o Other co-morbidities
- Study design (e.g., cohort, case control, cross-sectional, interventional)
- Descriptions of routine care, including whether the study was designed to influence retention
- Prior treatment exposure
o Proportion of adult patients ART treatment-naïve at initiation of ART
o Proportion of adult patients ART treatment-experienced at initiation of ART
o Basis for report of prior exposure (patient self-report, review of medical or laboratory records, tests of biological samples, pre-existing viral suppression, other)
- Authors' observations or comments on proportion of patients naïve or non-naïve, if any

Risk of bias (quality) assessment

Risk of bias and quality of individual studies that are included in this systematic review will be evaluated with the Joanna Briggs Institute critical appraisal checklist most relevant to the study type, as we expect that most included studies will be observational. The most important characteristics to be assessed will be recruitment biases (whether the study enrolled a representative sample of the specified population) and accuracy of naïve/non-naïve status reporting. Two reviewers will be involved in quality assessment, with disagreements resolved by a third reviewer on the study team.
We note that we expect that most studies meeting inclusion criteria for this review will report our outcomes (proportions naïve and non-naïve) as secondary outcomes or in their descriptions of their study cohorts, rather than as primary outcomes. (For example, a clinical trial with a primary outcome of viral suppression at 12 months after treatment initiation may report the proportion of participants who were naïve at initiation; our review would include the proportion naïve but not the primary outcome of viral suppression.)

Strategy for data synthesis

We will first present raw results from all included studies to demonstrate the breadth of outcomes, stratified by country. We will then estimate mean (95% confidence interval) and median (IQR) outcomes for each country and, to the extent that data allow, each population group or other stratification, such as setting. Pooled analysis of studies will only be conducted if ?3 studies are identified that report outcomes for the same populations (e.g., "adults initiating therapy at rural primary health clinics in Zambia").

Analysis of subgroups or subsets

If data allow, we plan to stratify our results by population (age, sex, condition, other characteristics) and facility characteristics (setting, level, size). We will not be able to specify all potential sub-group analyses until we determine which sub-groups are included in the publications reviewed.

Contact details for further information

Sydney Rosen

sbrosen@bu.edu

For peer review only

Organisational affiliation of the review
Boston University School of Public Health
https://www.bu.edu/sph/about/departments/global-health/

Review team members and their organisational affiliations
Dr Mariet Benade. Department of Global Health, Boston University School of Public Health, Boston, Massachusetts, USA
Dr Mhairi Maskew. Health Economics and Epidemiology Research Office, Faculty of Health Sciences, University of the Witwatersrand, Johannesburg, South Africa
Ms Linda Sande. Health Economics and Epidemiology Research Office, Faculty of Health Sciences, University of the Witwatersrand, Johannesburg, South Africa
Professor Nancy Scott. Department of Global Health, Boston University School of Public Health, Boston, Massachusetts, USA
Ms Allison Juntunen. Department of Global Health, Boston University School of Public Health, Boston, Massachusetts, USA
Professor Sydney Rosen. Department of Global Health, Boston University School of Public Health, Boston, Massachusetts, USA

Type and method of review
Epidemiologic, Systematic review

Anticipated or actual start date
10 April 2022

Anticipated completion date
10 July 2022

Funding sources/sponsors
Research reported in this publication was supported by the Bill & Melinda Gates Foundation Investment Number INV-031690, Retain6: Models of care for the first six months of HIV treatment.

Grant number(s)

State the funder, grant or award number and the date of award

Bill & Melinda Gates Foundation Investment Number INV-031690. October 2021.

Conflicts of interest

Language
English

Country
Malawi, South Africa, United States of America, Zambia

Stage of review
Review Ongoing

Subject index terms status
Subject indexing assigned by CRD

Subject index terms
MeSH headings have not been applied to this record

Date of registration in PROSPERO
08 May 2022

Date of first submission

For peer review only

07 April 2022

Stage of review at time of this submission

Stage	Started	Completed
Preliminary searches	Yes	No
Piloting of the study selection process	No	No
Formal screening of search results against eligibility criteria	No	No
Data extraction	No	No
Risk of bias (quality) assessment	No	No
Data analysis	No	No

The record owner confirms that the information they have supplied for this submission is accurate and complete and they understand that deliberate provision of inaccurate information or omission of data may be construed as scientific misconduct.
The record owner confirms that they will update the status of the review when it is completed and will add publication details in due course.

Versions

08 May 2022
08 May 2022

For peer review only

File S2. PRISMA checklists for manuscripts and abstracts

Topic	No.	Item	Location where item is reported
**TITLE**			
**Title**	1	Identify the report as a systematic review.	Title
**ABSTRACT**			
**Abstract**	2	See the PRISMA 2020 for Abstracts checklist	
**INTRODUCTION**			
**Rationale**	3	Describe the rationale for the review in the context of existing knowledge.	Page 2 (Objectives)
**Objectives**	4	Provide an explicit statement of the objective(s) or question(s) the review addresses.	Page 2 (Conclusions)
**METHODS**			
**Eligibility criteria**	5	Specify the inclusion and exclusion criteria for the review and how studies were grouped for the syntheses.	Page 6 (Search strategy, study selection and data extraction)
**Information sources**	6	Specify all databases, registers, websites, organisations, reference lists and other sources searched or consulted to identify studies. Specify the date when each source was last searched or consulted.	Page 6 & 7 (Search strategy, study selection and data extraction)
**Search strategy**	7	Present the full search strategies for all databases, registers and websites, including any filters and limits used.	Page 6, Supplementary Table 2 & 3
**Selection process**	8	Specify the methods used to decide whether a study met the inclusion criteria of the review, including how many reviewers screened each record and each report retrieved, whether they worked independently, and if applicable, details of automation tools used in the process.	Page 8 (Search strategy, study selection and data extraction)
**Data collection process**	9	Specify the methods used to collect data from reports, including how many reviewers collected data from each report, whether they worked independently, any processes for obtaining or confirming data from study investigators, and if applicable, details of automation tools used in the process.	Page 8 (Search strategy, study selection and data extraction)
**Data items**	10a	List and define all outcomes for which data were sought. Specify whether all results that were compatible with each outcome domain in each study were sought (e.g. for all measures, time points, analyses), and if not, the methods used to decide which results to collect.	Page 9 (Outcomes and analysis)
	10b	List and define all other variables for which data were sought (e.g. participant and intervention characteristics, funding sources). Describe any assumptions made about any missing or unclear information.	Page 9 (Outcomes and analysis)

For peer review only - http://bmjopen.bmj.com/site/about/guidelines.xhtml
For peer review only

Topic	No.	Item	Location where item is reported
**Study risk of bias assessment**	11	Specify the methods used to assess risk of bias in the included studies, including details of the tool(s) used, how many reviewers assessed each study and whether they worked independently, and if applicable, details of automation tools used in the process.	Page 9 (Outcomes and analysis)
**Effect measures**	12	Specify for each outcome the effect measure(s) (e.g. risk ratio, mean difference) used in the synthesis or presentation of results.	Page 9 (Outcomes and analysis)
**Synthesis methods**	13a	Describe the processes used to decide which studies were eligible for each synthesis (e.g. tabulating the study intervention characteristics and comparing against the planned groups for each synthesis (item 5)).	N/A
	13b	Describe any methods required to prepare the data for presentation or synthesis, such as handling of missing summary statistics, or data conversions.	N/A
	13c	Describe any methods used to tabulate or visually display results of individual studies and syntheses.	Page 9 (Outcomes and analysis)
	13d	Describe any methods used to synthesize results and provide a rationale for the choice(s). If meta-analysis was performed, describe the model(s), method(s) to identify the presence and extent of statistical heterogeneity, and software package(s) used.	N/A
	13e	Describe any methods used to explore possible causes of heterogeneity among study results (e.g. subgroup analysis, meta-regression).	Page 9 (Outcomes and analysis)
	13f	Describe any sensitivity analyses conducted to assess robustness of the synthesized results.	N/A
**Reporting bias assessment**	14	Describe any methods used to assess risk of bias due to missing results in a synthesis (arising from reporting biases).	Page 9 (Outcomes and analysis)
**Certainty assessment**	15	Describe any methods used to assess certainty (or confidence) in the body of evidence for an outcome.	N/A
**RESULTS**			
**Study selection**	16a	Describe the results of the search and selection process, from the number of records identified in the search to the number of studies included in the review, ideally using a flow diagram.	Page 9-10 (Results: Sources identified)
	16b	Cite studies that might appear to meet the inclusion criteria, but which were excluded, and explain why they were excluded.	Page 17 (Discussion)
**Study characteristics**	17	Cite each included study and present its characteristics.	Page 10-11 (Sources identified, Table 1)
**Risk of bias in studies**	18	Present assessments of risk of bias for each included study.	Page 15 (Quality of evidence)

For peer review only

Topic	No.	Item	Location where item is reported
**Results of individual studies**	19	For all outcomes, present, for each study: (a) summary statistics for each group (where appropriate) and (b) an effect estimate and its precision (e.g. confidence/credible interval), ideally using structured tables or plots.	Page 12-13 (Table 2)
**Results of syntheses**	20a	For each synthesis, briefly summarise the characteristics and risk of bias among contributing studies.	N/A
	20b	Present results of all statistical syntheses conducted. If meta-analysis was done, present for each the summary estimate and its precision (e.g. confidence/credible interval) and measures of statistical heterogeneity. If comparing groups, describe the direction of the effect.	N/A
	20c	Present results of all investigations of possible causes of heterogeneity among study results.	Page 14 (Proportions of patients non-naïve)
	20d	Present results of all sensitivity analyses conducted to assess the robustness of the synthesized results.	N/A
**Reporting biases**	21	Present assessments of risk of bias due to missing results (arising from reporting biases) for each synthesis assessed.	Page 15 (Quality of evidence)
**Certainty of evidence**	22	Present assessments of certainty (or confidence) in the body of evidence for each outcome assessed.	N/A
**DISCUSSION**			
**Discussion**	23a	Provide a general interpretation of the results in the context of other evidence.	Page 16 (Discussion)
	23b	Discuss any limitations of the evidence included in the review.	Page 19
	23c	Discuss any limitations of the review processes used.	Page 19
	23d	Discuss implications of the results for practice, policy, and future research.	Page 20-22
**OTHER INFORMATION**			
**Registration and protocol**	24a	Provide registration information for the review, including register name and registration number, or state that the review was not registered.	Page 2 (Abstract)
	24b	Indicate where the review protocol can be accessed, or state that a protocol was not prepared.	Page 2 (Abstract)
	24c	Describe and explain any amendments to information provided at registration or in the protocol.	Page 6 (Methods, File S1)
**Support**	25	Describe sources of financial or non-financial support for the review, and the role of the funders or sponsors in the review.	Page 22 (Funding)

For peer review only

Topic	No.	Item	Location where item is reported
**Competing interests**	26	Declare any competing interests of review authors.	Page 2 (Competing interests)
**Availability of data, code and other materials**	27	Report which of the following are publicly available and where they can be found: template data collection forms; data extracted from included studies; data used for all analyses; analytic code; any other materials used in the review.	Page 22 (Data availability statement)

For peer review only

ABSTRACT	No.	Item	Reported?
**TITLE**			
**Title**	1	Identify the report as a systematic review.	Yes
**BACKGROUND**			
**Objectives**	2	Provide an explicit statement of the main objective(s) or question(s) the review addresses.	Yes
**METHODS**			
**Eligibility criteria**	3	Specify the inclusion and exclusion criteria for the review.	Yes
**Information sources**	4	Specify the information sources (e.g. databases, registers) used to identify studies and the date when each was last searched.	Yes
**Risk of bias**	5	Specify the methods used to assess risk of bias in the included studies.	Yes
**Synthesis of results**	6	Specify the methods used to present and synthesize results.	Yes
**RESULTS**			
**Included studies**	7	Give the total number of included studies and participants and summarise relevant characteristics of studies.	Yes
**Synthesis of results**	8	Present results for main outcomes, preferably indicating the number of included studies and participants for each. If meta-analysis was done, report the summary estimate and confidence/credible interval. If comparing groups, indicate the direction of the effect (i.e. which group is favoured).	Yes
**DISCUSSION**			
**Limitations of evidence**	9	Provide a brief summary of the limitations of the evidence included in the review (e.g. study risk of bias, inconsistency and imprecision).	Yes
**Interpretation**	10	Provide a general interpretation of the results and important implications.	Yes
**OTHER**			
**Funding**	11	Specify the primary source of funding for the review.	Yes
**Registration**	12	Provide the register name and registration number.	Yes

For peer review only - http://bmjopen.bmj.com/site/about/guidelines.xhtml

Supplemental file 3:
Systematic reviews screened for potential articles

1. Abdulrahman SA, Ganasegeran K, Rampal L, Martins OF. HIV Treatment Adherence - A Shared Burden for Patients, Health-Care Providers, and Other Stakeholders. AIDS Rev. 2019;21: 28–39. doi:10.24875/AIDSRev.19000037
2. Abebe Moges N, Olubukola A, Micheal O, Berhane Y. HIV patients retention and attrition in care and their determinants in Ethiopia: a systematic review and meta-analysis. BMC Infect Dis. 2020;20: 439. doi:10.1186/s12879-020-05168-3
3. Adugna Wubneh C, Dessalegn Mekonnen B, Wesenyeleh Delelegn M, Asmare Atalell K. Adherence to option B+ and its association with disclosure status and counseling among HIV-positive pregnant and lactating women in Ethiopia: systematic review and meta-analysis. Public Health. 2022;211: 105–113. doi:10.1016/J.PUHE.2022.07.016
4. Agutu CA, Ngetsa CJ, Price MA, Rinke de Wit TF, Omosa-Manyonyi G, Sanders EJ, et al. Systematic review of the performance and clinical utility of point of care HIV-1 RNA testing for diagnosis and care. PLoS One. 2019;14: e0218369. doi:10.1371/journal.pone.0218369
5. Ale BM, Amahowe F, Nganda MM, Danwang C, Wakaba NN, Almuwallad A, et al. Global burden of active smoking among people living with HIV on antiretroviral therapy: a systematic review and meta-analysis. Infect Dis Poverty. 2021;10: 12. doi:10.1186/s40249-021-00799-3
6. Belayneh Z, Mekuriaw B, Mehare T, Shumye S, Tsehay M. Magnitude and predictors of common mental disorder among people with HIV/AIDS in Ethiopia: a systematic review and meta-analysis. BMC Public Health. 2020;20: 689.
7. Brault MA, Spiegelman D, Abdool Karim SS, Vermund SH. Integrating and Interpreting Findings from the Latest Treatment as Prevention Trials. Curr HIV/AIDS Rep. 2020;17: 249–258. doi:10.1007/s11904-020-00492-4
8. Bulstra C, Hontelez J, Otto M, Stepanova A, Lamontagne E, Yakusik A, et al. Integrating HIV services and other health services: A systematic review and meta-analysis. Nosyk B, editor. PLoS Med. 2021;18: e1003836. doi:10.1371/journal.pmed.1003836
9. Casale M, Carlqvist A, Cluver L. Recent Interventions to Improve Retention in HIV Care and Adherence to Antiretroviral Treatment Among Adolescents and Youth: A Systematic Review. AIDS Patient Care STDS. 2019;33: 237–252.
10. Chammartin F, Zürcher K, Keiser O, Weigel R, Chu K, AN K, et al. Outcomes of Patients Lost to Follow-up in African Antiretroviral Therapy Programs: Individual Patient Data Meta-analysis. Clin Infect Dis an Off Publ Infect Dis Soc Am. 2018;67: 1643–1652. doi:10.1093/cid/ciy347
11. Chem ED, Van Hout MC, Hope V. Treatment outcomes and antiretroviral uptake in multidrug-resistant tuberculosis and HIV co-infected patients in Sub Saharan Africa: a systematic review and meta-analysis. BMC Infect Dis. 2019;19.
12. Chem E, Ferry A, Seeley J, HA W, Simms V. Health-related needs reported by adolescents living with HIV and receiving antiretroviral therapy in sub-Saharan Africa: a systematic literature review. J Int AIDS Soc. 2022;25: e25921. doi:10.1002/jia2.25921
13. Chimatira R, Ross A. A rapid review and synthesis of the effectiveness of programmes

For peer review only

initiating community-based antiretroviral therapy in sub-Saharan Africa. South Afr J HIV Med. 2020;21. doi:10.4102/SAJHIVMED.V21I1.1153
14. Cohn J, Ake J, Moorhouse M, Godfrey C. Sex Differences in the Treatment of HIV. Curr HIV/AIDS Rep. 2020;17: 373–384. doi:10.1007/s11904-020-00499-x
15. Damtie Y, Kefale B, Yalew M, Arefaynie M, Adane B, Muche A, et al. HIV risk behavior and associated factors among people living with HIV/AIDS in Ethiopia: A systematic review and meta-analysis. PLoS One. 2022;17: e0269304. doi:10.1371/journal.pone.0269304
16. Damulak PP, Ismail S, Abdul Manaf R, Mohd Said S, Agbaji O. Interventions to Improve Adherence to Antiretroviral Therapy (ART) in Sub-Saharan Africa: An Updated Systematic Review. Int J Env Res Public Heal. 2021;18. doi:10.3390/ijerph18052477
17. De Tomasi K, Mouala C. Good practices for retention in the circuit of prevention of Mother-Child Transmission of HIV in Sub-Saharan Africa: a systematic review of the literature. Med Sante Trop. 2019;29: 279–286. doi:10.1684/mst.2019.0911
18. Demena BA, Artavia-Mora L, Ouedraogo D, Thiombiano BA, Wagner N. A Systematic Review of Mobile Phone Interventions (SMS/IVR/Calls) to Improve Adherence and Retention to Antiretroviral Treatment in Low-and Middle-Income Countries. AIDS Patient Care STDS. 2020;34: 59–71. doi:10.1089/apc.2019.0181
19. Dessie G, Wagnew F, Mulugeta H, Amare D, Jara D, CT L, et al. The effect of disclosure on adherence to antiretroviral therapy among adults living with HIV in Ethiopia: a systematic review and meta-analysis. BMC Infect Dis. 2019;19: 528.
20. Diallo M, Adekpedjou R, Ahouada C, Ngangue P, Ly BA. Impact of pre-antiretroviral therapy cd4 counts on drug resistance and treatment failure: A systematic review. AIDS Rev. 2020;22: 78–92. doi:10.24875/AIDSRev.20000012
21. Dovel KL, Hariprasad S, Hubbard J, Cornell M, Phiri K, Choko A, et al. Strategies to improve antiretroviral therapy (ART) initiation and early engagement among men in sub-Saharan Africa: A scoping review of interventions in the era of universal treatment. Trop Med Int Heal. 2023;28: 454–465. doi:10.1111/tmi.13880
22. Duffy M, Madevu-Matson C, JE P, Zwick H, Sharer M, AM P. Systematic review: Development of a person-centered care framework within the context of HIV treatment settings in sub-Saharan Africa. Trop Med Int Health. 2022;27: 479–493. doi:10.1111/tmi.13746
23. Edessa D, Sisay M, Asefa F. Second-line HIV treatment failure in sub-Saharan Africa: A systematic review and meta-analysis. PLoS One. 2019;14: e0220159. doi:10.1371/journal.pone.0220159
24. Endalamaw A, Mekonnen M, Geremew D, Yehualashet FA, Tesera H, Habtewold TD. HIV/AIDS treatment failure and associated factors in Ethiopia: meta-analysis. BMC Public Health. 2020;20: 82. doi:10.1186/s12889-020-8160-8
25. Enslin D, Mallya P. Factors influencing treatment adherence in hypertension and HIV management in South Africa: A comparative literature review. South African Fam Pract. 2022;64. doi:10.4102/SAFP.V64I1.5434
26. Eshun-Wilson I, Awotiwon AA, Germann A, Amankwaa SA, Ford N, Schwartz S, et al. Effects of community-based antiretroviral therapy initiation models on HIV treatment outcomes: A systematic review and meta-analysis. PLoS Med. 2021;18: e1003646. doi:10.1371/journal.pmed.1003646

For peer review only

27. Finlay J, Lambert T, Krahn J, Meyer G, Singh AE, Caine V. Incentive-Based Human Immunodeficiency Virus Screening in Low- and Middle-Income Countries: A Systematic Review. Sex Transm Dis. 2022;49: 274–283. doi:10.1097/OLQ.0000000000001567
28. Fite RO. Association between adherence to Antiretroviral Therapy and place of residence among adult HIV infected patients in Ethiopia: A systematic review and meta-analysis. PLoS One. 2021;16: e0256948. doi:10.1371/journal.pone.0256948
29. Ford N, Migone C, Calmy A, Kerschberger B, Kanters S, Nsanzimana S, et al. Benefits and risks of rapid initiation of antiretroviral therapy. Aids. 2018;32: 17–23. doi:10.1097/qad.0000000000001671
30. Frijters EM, Hermans LE, Wensing AMJ, Devillé WLJM, Tempelman HA, De Wit JBF. Risk factors for loss to follow-up from antiretroviral therapy programmes in low-income and middle-income countries. AIDS. 2020;34: 1261–1288.
31. Fuseini H, Gyan BA, Kyei GB, Heimburger DC, Koethe JR. Undernutrition and HIV Infection in Sub-Saharan Africa: Health Outcomes and Therapeutic Interventions. Curr HIV/AIDS Rep. 2021;18: 87–97. doi:10.1007/s11904-021-00541-6
32. Geremew D, Melku M, Endalamaw A, Woldu B, Fasil A, Negash M, et al. Tuberculosis and its association with CD4(+) T cell count among adult HIV positive patients in Ethiopian settings: a systematic review and meta-analysis. BMC Infect Dis. 2020;20: 325.
33. Hagey JM, Li X, Barr-Walker J, Penner J, Kadima J, Oyaro P, et al. Differentiated HIV care in sub-Saharan Africa: a scoping review to inform antiretroviral therapy provision for stable HIV-infected individuals in Kenya. AIDS Care. 2018;30: 1477–1487. doi:10.1080/09540121.2018.1500995
34. Haghighat R, Steinert J, Cluver L. The effects of decentralising antiretroviral therapy care delivery on health outcomes for adolescents and young adults in low- and middle-income countries: a systematic review. Glob Health Action. 2019;12: 1668596. doi:10.1080/16549716.2019.1668596
35. Hargreaves J, Dalal S, Rice B, Anderegg N, Bhattacharjee P, Gafos M, et al. Repositioning Implementation Science in the HIV Response: Looking Ahead From AIDS 2018. J Acquir Immune Defic Syndr. 2019;82: S299–S304. doi:10.1097/QAI.0000000000002209
36. Hlongwa M, Mashamba-Thompson T, Makhunga S, Hlongwana K. Mapping evidence of intervention strategies to improving men's uptake to HIV testing services in sub-Saharan Africa: A systematic scoping review. BMC Infect Dis. 2019;19: 496. doi:10.1186/s12879-019-4124-y
37. Ibiloye O, Masquillier C, Jwanle P, S VB, J van O, Lynen L, et al. Community-Based ART Service Delivery for Key Populations in Sub-Saharan Africa: Scoping Review of Outcomes Along the Continuum of HIV Care. AIDS Behav. 2022;26: 2314–2337. doi:10.1007/s10461-021-03568-3
38. Jardim C, Zamani R, Akrami M. Evaluating the Impact of the COVID-19 Pandemic on Accessing HIV Services in South Africa: A Systematic Review. Int J Environ Res Public Health. 2022;19. doi:10.3390/ijerph191911899
39. Jopling R, Nyamayaro P, Andersen LS, Kagee A, Haberer JE, Abas MA. A Cascade of Interventions to Promote Adherence to Antiretroviral Therapy in African Countries. Curr HIV/AIDS Rep. 2020;17: 529–546. doi:10.1007/s11904-020-00511-4
40. Kadia BM, Dimala CA, Fongwen NT, Smith AD. Barriers to and enablers of uptake of

For peer review only

antiretroviral therapy in integrated HIV and tuberculosis treatment programmes in sub-Saharan Africa: a systematic review and meta-analysis. AIDS Res Ther. 2021;18. doi:10.1186/s12981-021-00395-3
41. Kebede HK, Mwanri L, Ward P, Gesesew HA. Predictors of lost to follow up from antiretroviral therapy among adults in sub-Saharan Africa: a systematic review and meta-analysis. Infect Dis POVERTY. 2021;10. doi:10.1186/s40249-021-00822.
42. Knight L, FC M, Schatz E. Behavioral and cognitive interventions to improve treatment adherence and access to HIV care among older adults in sub-Saharan Africa: an updated systematic review. Syst Rev. 2018;7: 114.
43. Kusemererwa S, Akena D, Nakanjako D, Kigozi J, Nanyunja R, Nanfuka M, et al. Strategies for retention of heterosexual men in HIV care in sub-Saharan Africa: A systematic review. PLoS One. 2021;16: e0246471. doi:10.1371/journal.pone.0246471
44. Laurenzi CA, du Toit S, Ameyan W, Melendez-Torres GJ, Kara T, Brand A, et al. Psychosocial interventions for improving engagement in care and health and behavioural outcomes for adolescents and young people living with HIV: a systematic review and meta-analysis. J Int AIDS Soc. 2021;24.
45. Le Tourneau N, Germann A, Thompson RR, Ford N, Schwartz S, Beres L, et al. Evaluation of HIV treatment outcomes with reduced frequency of clinical encounters and antiretroviral treatment refills: A systematic review and meta-analysis. PLoS Med. 2022;19: e1003959. doi:10.1371/journal.pmed.1003959
46. Limbada M, Zijlstra G, Macleod D, Ayles H, Fidler S. A systematic review of the effectiveness of non- health facility based care delivery of antiretroviral therapy for people living with HIV in sub-Saharan Africa measured by viral suppression, mortality and retention on ART. BMC Public Health. 2021;21: 1110. doi:10.1186/s12889-021-11053-8
47. Long L, Kuchukhidze S, Pascoe S, BE N, MP F, Cele R, et al. Retention in care and viral suppression in differentiated service delivery models for HIV treatment delivery in sub-Saharan Africa: a rapid systematic review. J Int AIDS Soc. 2020;23: e25640. doi:10.1002/jia2.25640
48. Makhado L, Mongale MP. Factors influencing non-adherence to antiretroviral therapy in South Africa: A systematic review. HIV AIDS Rev. 2019;18: 239–246.
49. Mateo-Urdiales A, Johnson S, Smith R, Nachega JB, Eshun-Wilson I. Rapid initiation of antiretroviral therapy for people living with HIV. Cochrane Database Syst Rev. 2019;2019. doi:10.1002/14651858.CD012962.pub2
50. Menon S, Benova L, Mabeya H. Epilepsy management in pregnant HIV plus women in sub-Saharan Africa, clinical aspects to consider: a scoping review. BMC Med. 2020;18.
51. Mugglin C. Gueler A., Vanobberghen F., Rice B., Egger M. KD. Apples and oranges: Assessment of the care cascade in sub-Saharan Africa. 22. doi:10.1002/jia2.25327
52. Muhula S, Gachohi J, Kombe Y, Karanja S. Interventions to improve early retention of patients in antiretroviral therapy programmes in sub-Saharan Africa: A systematic review. PLoS One. 2022;17: e0263663. doi:10.1371/journal.pone.0263663
53. Mukumbang FC, Orth Z, Van Wyk B. What do the implementation outcome variables tell us about the scaling-up of the antiretroviral treatment adherence clubs in South Africa? A document review. Heal Res Policy Syst. 2019;17.
54. Musumari PM, Techasrivichien T, Srithanaviboonchai K, Wanyenze RK, Matovu JKB,

For peer review only

Poudyal H, et al. HIV epidemic in fishing communities in Uganda: A scoping review. PLoS One. 2021;16.
55. Necho M, Belete A, Tsehay M. Depressive symptoms and their determinants in patients who are on antiretroviral therapy in the case of a low-income country, Ethiopia: a systematic review and meta-analysis. Int J Ment Health Syst. 2021;15. doi:10.1186/s13033-020-00430-2
56. Nyato D, Kuringe E, Drake M, Casalini C, Nnko S, Shao A, et al. Participants' accrual and delivery of HIV prevention interventions among men who have sex with men in sub-Saharan Africa: a systematic review. BMC Public Health. 2018;18: 370. doi:10.1186/s12889-018-5303-2
57. Nyoni T, Sallah YH, Okumu M, Byansi W, Lipsey K, Small E. The effectiveness of treatment supporter interventions in antiretroviral treatment adherence in sub-Saharan Africa: a systematic review and meta-Analysis. AIDS Care Psychol Socio-Med Asp AIDS HIV. 2020;32: 214–227.
58. Okonji EF, Mukumbang FC, Orth Z, Vickerman-Delport SA, Van Wyk B. Psychosocial support interventions for improved adherence and retention in ART care for young people living with HIV (10-24,Ââyears): a scoping review. BMC Public Health. 2020;20: 1841. doi:10.1186/s12889-020-09717-y
59. Omonaiye O, Kusljic S, Nicholson P, Manias E. Medication adherence in pregnant women with human immunodeficiency virus receiving antiretroviral therapy in sub-Saharan Africa: a systematic review. BMC Public Health. 2018;18: 805. doi:10.1186/s12889-018-5651-y
60. Omonaiye O, Nicholson P, Kusljic S, Manias E. A meta-analysis of effectiveness of interventions to improve adherence in pregnant women receiving antiretroviral therapy in sub-Saharan Africa. Int J Infect Dis IJID Off Publ Int Soc Infect Dis. 2018;74: 71–82.
61. Owusu K.K. Ahmed Z. A-GR. Strategies to improve linkage to hiv care in urban areas of Sub-Saharan Africa: A systematic review. 11. doi:10.2147/HIV.S216093
62. Passchier RV, Abas MA, Ebuenyi ID, Pariante CM. Effectiveness of depression interventions for people living with HIV in Sub-Saharan Africa: A systematic review & meta-analysis of psychological & immunological outcomes. Brain Behav Immun. 2018;73: 261–273. doi:10.1016/j.bbi.2018.05.010
63. Pellowski JA, Price DM, Harrison AD, Tuthill EL, Myer L, Operario D, et al. A Systematic Review and Meta-analysis of Antiretroviral Therapy (ART) Adherence Interventions for Women Living with HIV. AIDS Behav. 2019;23: 1998–2013. doi:10.1007/s10461-018-2341-9
64. Penn A, Azman H, Horvath H, KD T, MD H, Rajan J, et al. Supportive interventions to improve retention on ART in people with HIV in low- and middle-income countries: A systematic review. PloS one. 2018. p. e0208814. doi:10.1371/journal.pone.0208814
65. Rapaport SF, Peer AD, Viswasam N, Hahn E, Ryan S, Turpin G, et al. Implementing HIV Prevention in Sub-Saharan Africa: A Systematic Review of Interventions Targeting Systems, Communities, and Individuals. AIDS Behav. doi:10.1007/s10461-022-03751-0
66. Raphael M, Elizabeth S, Fiona V, Maja W. "Linkage to care" among people living with HIV in the era of "universal test and treat" in a sub-Sahara African setting. SWISS Med Wkly.

For peer review only

2021;151. doi:10.4414/smw.2021.20535

67. Roy M, Bolton Moore C, Sikazwe I, Holmes CB. A Review of Differentiated Service Delivery for HIV Treatment: Effectiveness, Mechanisms, Targeting, and Scale. Current HIV/AIDS Reports. Current Medicine Group LLC 1; 2019. pp. 324–334. doi:10.1007/s11904-019-00454-5
68. Sabapathy K, Hensen B, Varsaneux O, Floyd S, Fidler S, Hayes R. The cascade of care following community-based detection of HIV in sub-Saharan Africa – A systematic review with 90-90-90 targets in sight. PLoS One. 2018;13: e0200737. doi:10.1371/JOURNAL.PONE.0200737
69. Takarinda KC, Wallenta J, Scheve A, Mody A, Apollo T, Harries AD, et al. Measuring Retention in Antiretroviral Therapy Programs—a Synthetic Review of Different Approaches for Field Use in Low- and Middle-Income Settings. Curr Trop Med Reports. 2018;5: 179–185. doi:10.1007/S40475-018-0153-7
70. Too EK, Abubakar A, Nasambu C, Koot HM, Cuijpers P, Newton CRJC, et al. Prevalence and factors associated with common mental disorders in young people living with HIV in sub-Saharan Africa: a systematic review. J Int AIDS Soc. 2021;24.
71. Vrazo A, Firth J, Amzel A, Sedillo R, Ryan J, BR P. Interventions to significantly improve service uptake and retention of HIV-positive pregnant women and HIV-exposed infants along the prevention of mother-to-child transmission continuum of care: systematic review. Trop Med Int Health. 2018;23: 136–148. doi:10.1111/tmi.13014
72. Walsh F, Khan S, Bärnighausen T, Hettema A, Lejeune C, Mazibuko S, et al. Getting to 90-90-90: Experiences from the MaxART Early Access to ART for All (EAAA) Trial in Eswatini. Curr HIV/AIDS Rep. 2020;17: 324–332. doi:10.1007/s11904-020-00501-6
73. Yah CS, Tambo E. Why is mother to child transmission (MTCT) of HIV a continual threat to new-borns in sub-Saharan Africa (SSA). J Infect Public Health. 2019;12: 213–223.
74. Yendewa GA, Poveda E, Yendewa SA, Sahr F, Quiñones-Mateu ME, Salata RA. HIV/AIDS in Sierra Leone: Characterizing the Hidden Epidemic. AIDS Rev. 2018;20: 104–113. doi:10.24875/AIDSRev.M18000022
75. Yonga AM, Kiss L, Onarheim KH. A systematic review of the effects of intimate partner violence on HIV-positive pregnant women in sub-Saharan Africa. BMC Public Health. 2022;22: 220.
76. Zanoni B, Archary M, Sibaya T, Ramos T, Donenberg G, Shahmanesh M, et al. Interventions addressing the adolescent HIV continuum of care in South Africa: a systematic review and modified Delphi analysis. BMJ Open. 2022;12: e057797. doi:10.1136/bmjopen-2021-057797

For peer review only

Additional table S1. Inclusion/exclusion criteria

Parameter	Inclusion criteria	Exclusion criteria
Population	Ages 18+ years; confirmed HIV positive status; presenting for initiation of any regimen of lifelong antiretroviral treatment	Paediatric and adolescent populations; currently only receiving ART for HIV prevention (PEP or PrEP)
Geographic region	Sub-Saharan Africa	None
Intervention	None, observational descriptive outcome	None
Study design	Reports primary, patient-level data from retrospective or prospective cohorts collected under any study design (trial, observational) with or without a comparison group; systematic reviews, meta-analyses	Case series or reports, purely qualitative studies, treatment guidelines, mathematical models, editorials, commentaries, study or trial protocols
Required descriptive data	Describes all of patients, location, timing of ART initiation, facility type, service delivery models and services provided to the public sector through government-managed public health infrastructure or through NGO/private programs or facilities that serve the uninsured sector	Insufficient description of the characteristics needed to describe the study population and outcome
Comparator	Not required; single arm evaluations are eligible	None
Outcomes	Reports proportion of patients initiating ART that are ART naïve and proportions of patients previously experienced on ART for any duration after initiation.	Insufficient detail provided to estimate of outcome
Timing	A majority of data collected for ART initiation on or after January 1, 2016	A majority of data accrued before January 1, 2016

For peer review only

For peer review only - http://bmjopen.bmj.com/site/about/guidelines.xhtml

Additional table S2. Search strategy – conducted on 11 July 2023

**EMBASE**	
Population	('human immunodeficiency virus infection'/exp OR 'hiv infection')<br>**AND**
Intervention	('therapy'/exp OR ('anti human immunodeficiency virus agent'/exp OR 'antiretrovirus agent'/exp OR 'highly active antiretroviral therapy'/exp))<br>**AND**
Outcomes	('patient compliance'/exp OR 'treatment adherence' OR undisclosed OR 'retention'/exp)<br>**AND**
Context	(('africa south of the sahara'/exp OR 'africa south of the sahara' OR 'black africa' OR 'sub saharan africa' OR 'subsaharan africa') OR ('angola'/exp OR angola OR 'benin'/exp OR benin OR 'botswana'/exp OR botswana OR 'burkina faso'/exp OR 'burkina faso' OR 'burundi'/exp OR burundi OR 'cameroon'/exp OR cameroon OR 'cape verde'/exp OR 'cape verde' OR 'central african republic'/exp OR 'central african republic' OR 'chad'/exp OR chad OR 'comoros'/exp OR comoros OR 'congo'/exp OR congo OR brazzaville OR 'cote d ivoire' OR 'djibouti'/exp OR djibouti OR 'equatorial guinea'/exp OR 'equatorial guinea' OR 'eritrea'/exp OR eritrea OR 'ethiopia'/exp OR ethiopia OR 'gabon'/exp OR gabon OR 'gambia'/exp OR gambia OR 'ghana'/exp OR ghana OR 'guinea bissau'/exp OR 'guinea bissau' OR 'kenya'/exp OR kenya OR 'lesotho'/exp OR lesotho OR 'liberia'/exp OR liberia OR 'madagascar'/exp OR madagascar OR 'malawi'/exp OR malawi OR 'mali'/exp OR mali OR 'mauritania'/exp OR mauritania OR 'mauritius'/exp OR mauritius OR 'mozambique'/exp OR mozambique OR 'namibia'/exp OR namibia OR 'niger'/exp OR niger OR 'nigeria'/exp OR nigeria OR 'rwanda'/exp OR rwanda OR 'sao tome e principe' OR 'senegal'/exp OR senegal OR 'seychelles'/exp OR seychelles OR 'sierra leone'/exp OR 'sierra leone' OR 'somalia'/exp OR somalia OR 'south africa'/exp OR 'south africa' OR 'south sudan'/exp OR 'south sudan' OR 'sudan'/exp OR sudan OR 'swaziland'/exp OR swaziland OR 'tanzania'/exp OR tanzania OR 'togo'/exp OR togo OR 'uganda'/exp OR uganda OR 'western sahara'/exp OR 'western sahara' OR (western AND ('sahara'/exp OR sahara)) OR 'zaire'/exp OR zaire OR 'zambia'/exp OR zambia OR 'zimbabwe'/exp OR zimbabwe)) AND [2018-2023]/py
**PubMed**	
Population	("HIV Infections"[Mesh] OR HIV Infection)<br>**AND**
Intervention	((treatment OR "Anti-HIV Agents"[MESH] OR "Anti-Retroviral Agents"[MESH] OR "Antiretroviral Therapy, Highly Active"[MESH])<br>**AND**
Outcomes	(compliance OR "Treatment adherence" OR undisclosed OR retention)<br>**AND**
Context	("Africa South of the Sahara"[Mesh] OR Sub-Saharan Africa OR Subsaharan Africa OR Africa, Sub-Saharan OR Africa South of the Sahara OR Angola OR Benin OR Botswana OR "Burkina Faso" OR Burundi OR Cameroon OR "Cape Verde" OR "Central African Republic" OR Chad OR Comoros OR Congo OR Brazzaville OR "Cote d'Ivoire" OR Djibouti OR "Equatorial Guinea" OR Eritrea OR Ethiopia OR Gabon OR Gambia OR Ghana OR "Guinea Bissau" OR Kenya OR Lesotho OR Liberia OR Madagascar OR Malawi OR Mali OR Mauritania OR Mauritius OR Mozambique OR Namibia OR Niger OR Nigeria OR Rwanda OR "Sao Tome e Principe" OR Senegal OR Seychelles OR "Sierra Leone" OR Somalia OR "South

For peer review only

	Africa" OR "South Sudan" OR Sudan OR Swaziland OR Tanzania OR Togo OR Uganda OR Western Sahara OR Zaire OR Zambia OR Zimbabwe) AND (2018:2023[pdat])
**Web of Science Core Collection***	
Population	TS=("HIV Infections" OR HIV Infection)<br>**AND**
Intervention	TS=(treatment OR "Anti-HIV Agents" OR "Anti-Retroviral Agents" OR "Antiretroviral Therapy, Highly Active")<br>**AND**
Outcomes	TS= (compliance OR "Treatment adherence" OR undisclosed OR retention)<br>**AND**
Context	TS=("Africa South of the Sahara" OR Sub-Saharan Africa OR Subsaharan Africa OR Africa, Sub-Saharan OR Africa South of the Sahara OR Angola OR Benin OR Botswana OR "Burkina Faso" OR Burundi OR Cameroon OR "Cape Verde" OR "Central African Republic" OR Chad OR Comoros OR Congo OR Brazzaville OR "Cote d'Ivoire" OR Djibouti OR "Equatorial Guinea" OR Eritrea OR Ethiopia OR Gabon OR Gambia OR Ghana OR "Guinea Bissau" OR Kenya OR Lesotho OR Liberia OR Madagascar OR Malawi OR Mali OR Mauritania OR Mauritius OR Mozambique OR Namibia OR Niger OR Nigeria OR Rwanda OR "Sao Tome e Principe" OR Senegal OR Seychelles OR "Sierra Leone" OR Somalia OR "South Africa" OR "South Sudan" OR Sudan OR Swaziland OR Tanzania OR Togo OR Uganda OR Western Sahara OR Zaire OR Zambia OR Zimbabwe)
**Conference searches**	
Search terms	“initiation”, “naïve”, “re-initiate”, “newly”, “experience”
Web URLs for conferences searched	International AIDS Conference<br>2018 - https://www.aids2018.org<br>2020 - https://www.aids2020.org<br>2022 - https://www.aids2022.org<br><br>International AIDS Society (IAS) Conference on HIV Science<br>2019 - https://programme.ias2019.org<br>2021 - https://ias2021.org<br><br>Conference on Retroviruses and Opportunistic Infections (CROI)<br>2018 - https://www.croiconference.org/croi-2018/<br>2019 - https://www.croiconference.org/croi-2019/<br>2020 - https://www.croiconference.org/croi-2020/<br>2021 - https://www.croiconference.org/croi-2021/<br>2022 - https://www.croiconference.org/croi-2022/

* Comprised of the Science Citation Index Expanded (1965 to the present), the Social Sciences Citation Index (1965 to the present), the Arts & Humanities Citation Index (1975 to the present), the Conference Proceedings Citation Index (both versions, Science and Social Sciences & the Humanities from 1990 to the present), the Book Citation Index (both versions, Science and Social Sciences & the Humanities from 2005 to the present) and the Emerging Sources Citation Index (2018 to the present)

Note: Results limited to 1 January 2018 – 11 July 2023

For peer review only - http://bmjopen.bmj.com/site/about/guidelines.xhtml

Additional table S3. Reasons for exclusion after full text review

Reason and number excluded	Explanation
Wrong time period (n=95)	All data or majority of data were gathered prior to 2016.
Wrong publication type (n=12)	Article was a protocol, review article or qualitative report without any quantitative descriptors.
Wrong population (n=129)	Population for main analysis included participants younger than 18yrs without stratification in a setting where adults are defined as older than 18yrs.<br>Population for main analysis excluded those with ART experience, unless other measures taken to confirm ART naivety. Population did not consists of participants presenting for ART initiation.
Not sufficient data for analysis (n=132)	No exclusion criteria met, but data reported was not sufficient for us to determine naïve vs non-naïve status.

For peer review only
